# Selective Chemical Manipulation of Mannosyl‐ and Galactosyl‐Queuosine in tRNAs of Living Cells

**DOI:** 10.1002/anie.202508499

**Published:** 2025-09-03

**Authors:** Alexander Pichler, Elsa Peev, Qingyi Ge, Ghofrane Ben Helal, Matthias Heiss, Stylianos Xefteris, Markus Müller, Thomas Carell

**Affiliations:** ^1^ Center for Nucleic Acid Therapeutics at the Department of Chemistry, Institute for Chemical Epigenetics Ludwig‐Maximilians‐Universität München Butenandtstr. 5‐13 81377 München Germany

**Keywords:** Epitranscriptomics, Galactosylation, Mannosylation, Queuine, Queuosine

## Abstract

Queuosine is a hypermodified nucleoside found in the anticodon loop of tRNAs decoding the amino acids His, Asn, Tyr, and Asp. In tRNA^Tyr^, the homoallylic 2‐hydroxyl group of the cyclopentene ring carries an additional β‐galactose, while in tRNA^Asp^, an α‐mannose is connected to the allylic 3‐hydroxyl group. Although these additional sugar modifications were found in the tRNAs of many animals, including humans, their purpose remains unknown. Recently, the Q‐tRNA galatosyl‐ and mannosyltransferase (QTGAL and QTMAN) that attach the galactose and mannose moieties to the different OH groups of the Q‐base have been identified, but the function of this transfer reaction remains unclear. Here, we performed feeding experiments with synthetic deoxyqueuine derivatives that lack either the allylic, the homoallylic, or both hydroxyl groups. Analysis of the queuosine derivatives that are found in the corresponding tRNAs shows that the glycosylases attach the sugar with precise specificity, suggesting decisive functions of the galactose and mannose sugar moieties in the decoding process.

## Graphical abstract

The Q‐base in the anticodon loop of certain eukaryotic tRNA is further mannosylated and galactosylated. Using feeding of chemically manipulated queuine derivatives, we show that the sugar‐modifying enzymes perform the modification chemistry with high regio‐ and chemoselectivity.

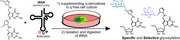

Decoding of the genetic information with tRNA carrying a defined amino acid requires precise control of the codon–anticodon interaction. Based on the three‐letter code, 61 different base triplets for 20 amino acids exist, which in humans are decoded by only 46 different tRNAs.^[^ ^1^ ^]^ As a consequence, some of these 46 tRNAs recognize multiple triplets. These synonymous triplets in the anticodon, encoding the same amino acid, vary in the first position (34, Figure 1), the so‐called wobble position. Queuosine (Q) is a hypermodified nucleoside found exclusively in this position 34, where it helps to decode the codons for the amino acids His (5′‐C‐A‐U/C‐3′), Asn (5′‐A‐A‐U/C‐3′), Tyr (5′‐U‐A‐U/C‐3′), and Asp (5′‐G‐A‐U/C‐3′). The exact role of the Q‐base in translation is controversially discussed,^[^ ^2^ ^]^ but it is agreed to facilitate the base pairing with both a C or U in the mRNA by establishing either a canonical G:C or a wobble G:U base pair (Figure 1a,b).^[^ ^3^ ^]^

**Figure 1:**
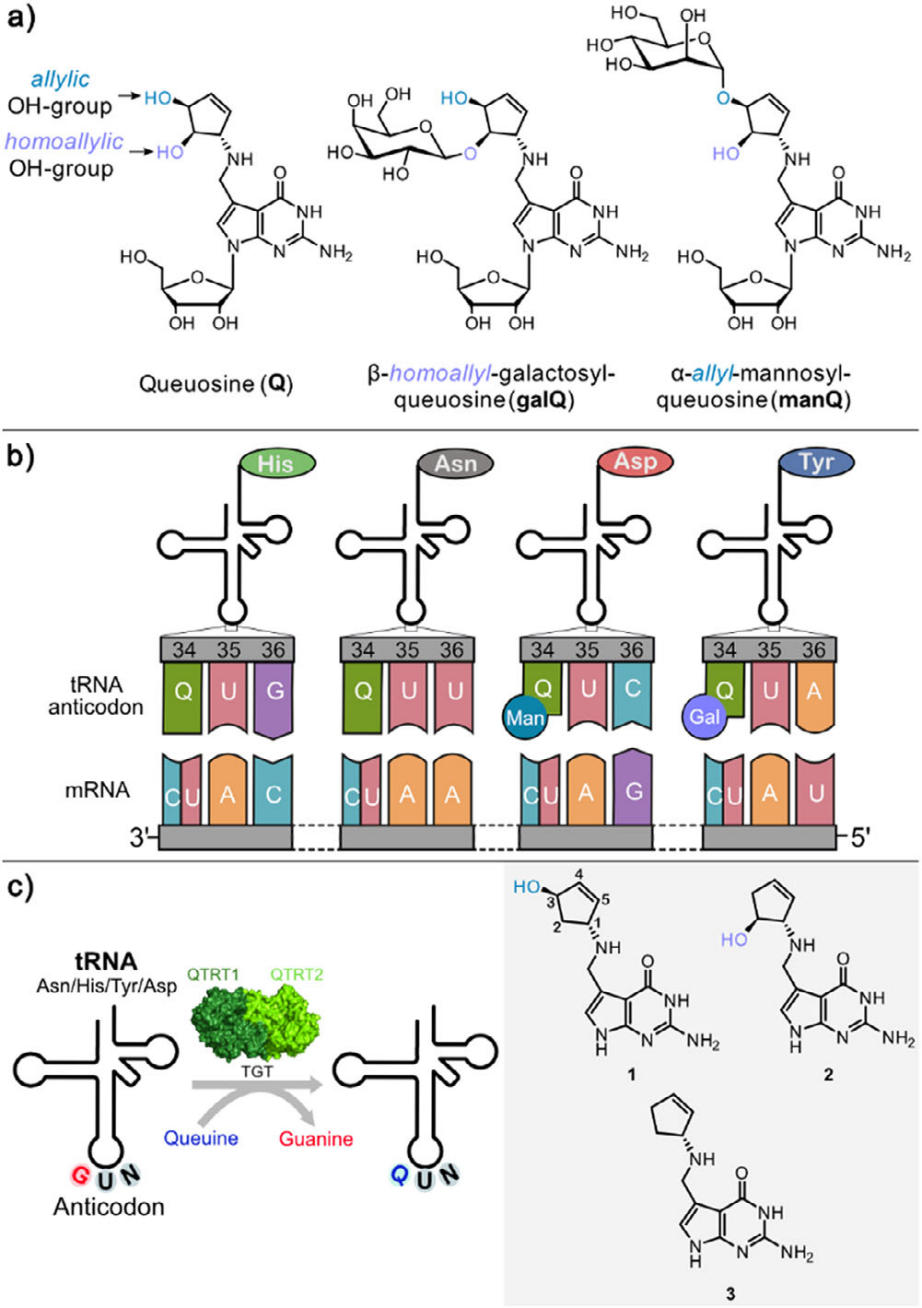
a) Depiction of the hypermodified nucleoside queuosine and its galactosylated **galQ** and mannosylated **manQ** versions. b) Illustration of the codon–anticodon base pairs in Q‐containing tRNAs. c) Transglycosylase ([TGT] consisting of the QTRT1 and QTRT2 subunits) catalyzed biosynthesis of modified queuosine by exchange of guanine at position 34 with deoxyqueuine derivatives 2‐deoxy‐queuine (2‐dq, **1**), 3‐deoxy‐queuine (3‐dq, **2**), and 2,3‐deoxy‐queuine (2,3‐dq, **3**) synthesized in this study.

In this context it is noteworthy that instead of Q, tRNA^Tyr^ (Q^34^‐U^35^‐A^36^) and tRNA^Asp^ (Q^34^‐U^35^‐C^36^) contain a galactosylated or mannosylated version of this base,^[^ ^4^ ^]^ respectively. Enigmatic was the observation that, despite the seemingly similar function, the homoallylic 2‐hydroxyl group of the cyclopentene ring system is β‐glycosylated with galactose,^[^ ^4^, ^5^ ^]^ while the mannosyl sugar is α‐glycosylated to the allylic 3‐hydroxy group (Figure 1a).^[^ ^6^ ^]^ Although the two different RNA glycosyltransferases (QTMAN and QTGAL) attaching the sugars to the Q‐base were recently identified and serve as an explanation for this inverted configuration, the functional role of the interesting regio‐selectivity remains unclear. In addition, the Suzuki group demonstrated via a knockout experiment of Q‐tRNA mannosyltransferase (QTMAN) and Q‐tRNA galactosyltransferase (QTGAL) that no glycosylation occurred, indicating that solely these enzymes are responsible for the sugar modification.^[^ ^7^ ^]^ Here, we wanted to investigate the structural determinants by which the different sugars are attached to the different OH‐groups of the Q‐base.

It is well established that in eukaryotes, the Q‐nucleosides in mature tRNAs are biosynthesized from the corresponding queuine precursor with the help of the eukaryotic TGT,^[^ ^8^ ^]^ which exchanges specifically the G residue at position 34 in the tRNA anticodon for the corresponding queuine (q) base, as illustrated in Figure 1c. The TGT enzymes are known to accept a wide range of queuine derivatives, allowing, in principle, the feeding of dehydroxylated queuine versions to study the regioselectivities of the glycosylases.^[^ ^9^ ^]^

We therefore planned feeding studies with the deoxy‐queuine derivatives 2‐deoxy‐queuine (2‐dq, **1**), 3‐deoxy‐queuine (3‐dq, **2**), and 2,3‐dideoxy‐queuine (2,3‐dq, **3**), which we hypothesized would get incorporated into the corresponding tRNAs.

The synthesis of 3‐dq **2** (Scheme 1‐i) started with the epoxidation of cyclopentadiene (**CpH**) to **4,** followed by the opening of the epoxide with methanolic ammonia to give (±)‐**5** and Boc‐protection of the amine to generate (±)‐**6**.^[^ ^10^, ^11^ ^]^ A kinetic racemate resolution with *Candida antarctica* Lipase B^[^ ^12^ ^]^ and isopropenyl acetate generated the *t*Bu‐(5*S*‐hydroxycyclopent‐2‐en‐1*S*‐yl)carbamate (+)‐**7** in 98% ee and 43% yield.^[^ ^13^ ^]^ TIPS‐protection to **8** and subsequent Boc‐deprotection furnished the building block **9** needed for the synthesis of 3‐dq **2**. For determining the enantiomeric purity via ^19^F NMR, we deprotected the Boc‐group in compound **8** and converted the amine into the respective Mosher Amide **10**.

**Scheme 1:**
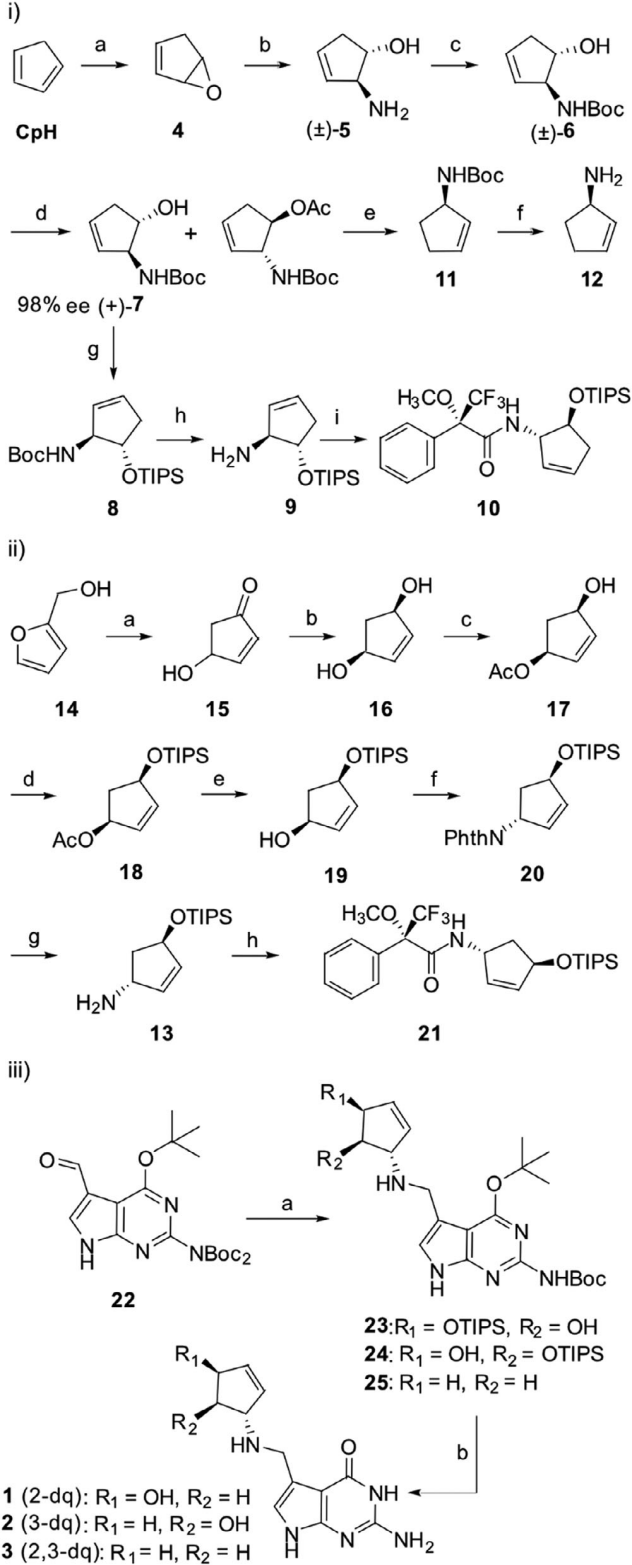
i) Synthesis of TIPS‐protected *S*‐amino‐2*R*‐hydroxy‐cyclopent‐4,5‐ene **9** and of 1‐*S*‐amino‐cyclopten‐4,5‐ene **12**. ii) Synthesis of TIPS‐protected 1*S*‐amino‐4*R*‐hydroxyl‐cyclopent‐2,3‐ene **13** as the key building block for the synthesis of iii) 4‐ and 5‐deoxyqueuine **1**, **2** as well as 4,5‐dideoxyqueuine **3**. Reaction conditions: (i) a) AcOOH, Na_2_CO_3_, NaOAc, DCM, 0 °C→r.t., 3 h, 63% b) NH_3_/MeOH, MeOH, 0 °C→r.t., 30 min, 52% c) Boc_2_O, EtOAc, NaHCO_3_, r.t., 5 h, 41% d) C*andida antarctica* Lipase‐B (10% wt), H_2_O, (pH 8), r.t., 24 h, 43%, 98%ee. e) 1) TCDI, imidazole, DCM, 24 °C, 24 h, 82%; 2) TTMS, AIBN, tol, 90 °C, 2.5 h 96%. f) TFA, DCM, 0 °C, 30 min, 99%. g) TIPS‐Cl, TEA, DMAP, DMF, r.t., 18 h, 88%. h) TFA, DCM, 0 °C, 20 min, 93% i) (*S*)‐(+)‐MTPA‐Cl, DIPEA, DCM, r.t., 18 h, 69%.  (ii) a) KH_2_PO_4_, H_2_O, 125 °C, 48 h, 40%. b) CeCl_3_, NaBH_4_, MeOH, −20 °C, 30 min, 74%. c) Pancreatin USP4, TEA, vinyl acetate, THF, r.t. 24 h. 53%, 99,9% ee. d) TIPS‐Cl, TEA, DMAP, DCM, r.t., 24 h, 65%. e) K_2_CO_3_, MeOH, r.t., 1.5 h, 90%. f) PPh_3_, DEAD, Phth, THF, r.t., 24 h, 68%. g) N_2_H_4_, EtOH, reflux, 5 h, 75%. h) (*S*)‐(+)‐MTPA‐Cl, DIPEA, DCM, r.t., 18 h, 69%. (iii) a1) **9**/**12/13,** EtOAc/DCM, r.t., 5 h. a2) NaBH(OAc)_3_, EtOAc/DCM, 0 °C→rt, 1 h. b) TFA, 2M HCl, 0 °C, 18 h. rp‐HPLC (H_2_O/MeCN, 0 → 15%, 45 min).

For the synthesis of the 2,3‐dideoxyqueuine **3**, we performed a Barton–McCombie deoxygenation with compound (+)‐**7**, which provided **11**, and after Boc‐deprotection, the 1*S*‐cyclopent‐2‐en‐1‐amine (**12**).^[^ ^14^ ^]^

A different route was taken for the synthesis of the TIPS‐protected 4*R*‐hydroxyl‐cyclopent‐2‐en‐1*R*‐amine **13**, as required for the synthesis of 2‐dq **1** (Scheme 1‐ii). Here, we started with the hydroxymethyl‐furane **14**, which was rearranged into 4‐hydroxy‐cyclopent‐2,3‐en (**15**). Luche reduction furnished the *syn*‐diole **16**. Kinetic racemate resolution with the enzyme pancreatin and vinylacetate furnished the 1*S*‐acetoxy‐4*R*‐hydroxycyclopent‐2‐enyl acetate (**17**) in 53% yield and 99.9% ee. TIPS‐protection to **18**,^[^ ^15^ ^]^ acetate cleavage to **19**, and conversion of the hydroxyl group into an amine using Mitsunobu conditions to **20**, followed by cleavage of the phthalimide with hydrazine, furnished the 1*S*‐amino‐4*R*‐TIPS‐protected‐hydroxyl‐cyclopent‐2‐ene **13** as the key building block for the synthesis of 2‐dq **1**. The enantiomeric purity of **13** was again verified via ^19^F NMR of the Mosher amide **21**.^[^ ^14^ ^]^

For the synthesis of the queuine derivatives **1–3** (Scheme 1‐iii), we utilized the Boc‐*tert*‐butyl protected 7‐formyldeazaguanine **22** ^[^ ^16^, ^17^ ^]^ and performed reductive amination reactions with the corresponding cyclopentene derivatives **9**,**12**, and **13**, followed by full deprotection of the coupled products **23–25** with trifluoroacetic acid and TBAF to obtain the deoxy‐queuine compounds **1–3**.

In order to examine whether the synthetic queuine derivatives would be incorporated into cellular tRNA^His^, tRNA^Asn^, tRNA^Tyr^, and tRNA^Asp^, we grew Human Embryonic Kidney cells (HEK293T) in minimal medium lacking queuine (Figures 2a and S2a). In order to observe the depletion of queuosine (**Q**) and in particular to determine the total lack of any Q‐bases in the HEK293T cells, the cells were cultivated for a few days and sampled daily. As described in the Supporting Information, we isolated and enriched the small RNAs from these cells and performed a total digest of the RNA down to the nucleoside level with a mixture of Benzonase, Calf Intestinal Alkaline Phosphatase (CIP), snake venom phosphodiesterase 1 (PDE1), as well as the protective agents pentostatine, tetrahydrouridine (THU), and butylated hydroxytoluene (BHT). This mixture was shown to be perfect for the full digestion of stably folded tRNAs.^[^ ^18^ ^]^

**Figure 2:**
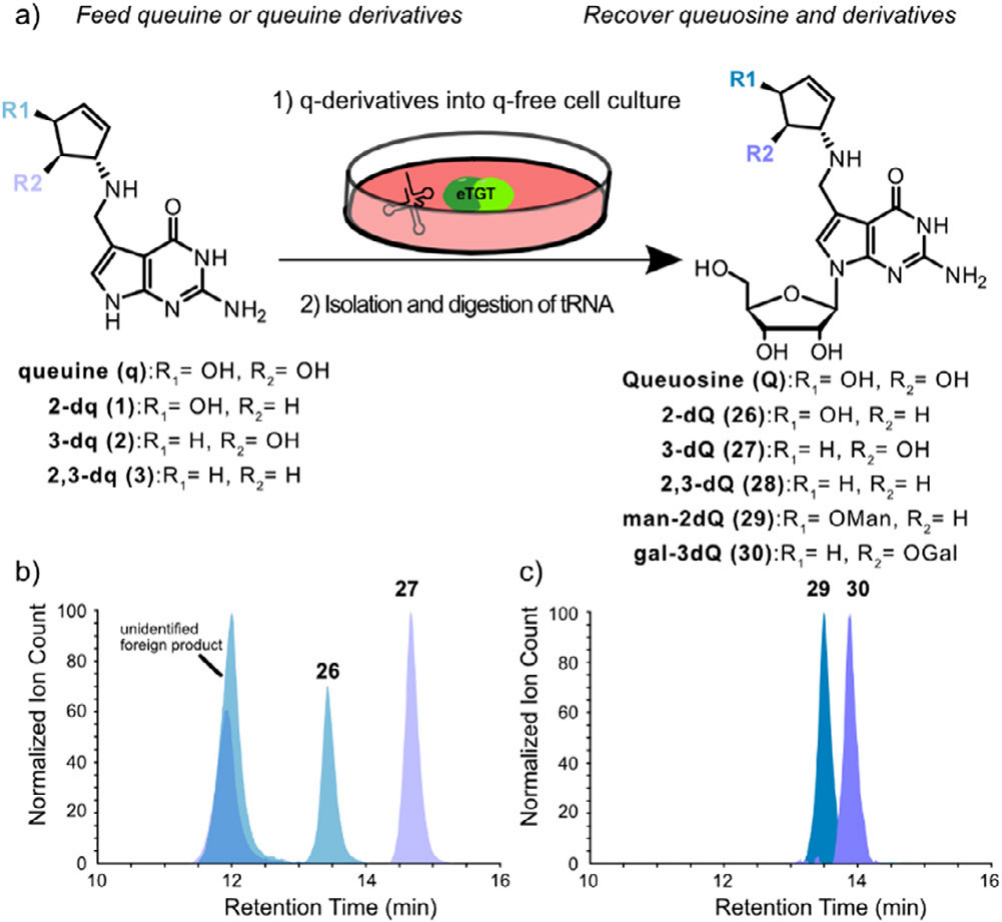
a) Scheme of the cell‐feeding strategy. Queuosine‐starved cells are fed with queuine (q), 2‐deoxy‐queuine (2‐dq, **1**), 3‐deoxy‐queuine (3‐dq, **2**), 2,3‐dideoxy‐queuine (2,3‐dq, **3**) or DMSO as a control. RNAs are isolated and digested to the nucleoside level, and the presence of queuosine and 2‐deoxy‐Q (2‐dQ, **26**), 3‐deoxy‐Q (3‐dQ, **27**), 2,3‐dideoxy‐Q (2,3‐dQ, **28**) as well as man‐2‐deoxy‐Q (man‐2dQ, **29**) and gal‐3‐deoxy‐Q (gal‐3dQ, **30**) was determined. b) UHPLC‐HRMS curve of 2‐dQ (**26**, 13,4 min, blue) combined with the 3‐dQ chromatogram (**27**, 14,7 min, purple). An unidentified foreign product at RT 11.6–12 min was found in all samples, including the DMSO control (Figure S4b). c) UHPLC‐HRMS profile of man‐2dQ (**29**, 13.5 min, dark blue) combined with gal‐3dQ (**30**, 14.7 min, dark purple) chromatogram. All data above are from cell samples harvested after 24 h with 20 µM treatment for each molecule and analyzed by HRMS. The chromatograms are shown together as a compiled plot profile.

UHPLC coupled to triple quadrupole mass spectrometry was subsequently employed to measure the content of Q, manQ, and galQ using protocols that we reported earlier.^[^ ^5^, ^18^ ^]^ As depicted in Figure S2a, the Q‐level indeed dropped continuously, and after about day 7, no further Q‐nucleosides could be detected. We therefore started the preparation of feeding experiments on day 9. Cells were split into five groups, and after an overnight rest, on day 10, we added to each portion medium supplemented with either queuine, 2‐deoxy‐queuine **1**, 3‐deoxy‐queuine **2**, 2,3‐dideoxy‐queuine **3**, or DMSO as a control. To ensure saturation of the Q‐tRNAs, each q‐compound was fed at a high concentration of 20 µM (Figure S2b–d). After 24 h, we isolated the small RNAs, digested them to the nucleoside level, using the method described above, and analyzed the obtained mixture by UHPLC‐HRMS and QQQ. The availability of some heavy‐labeled internal standards (ISTD) for queuosine allowed us to perform exact quantification using our established UHPLC‐QQQ method.^[^ ^6^ ^]^ Under the assumption that there are no severe ionization differences between the natural Q‐derivatives and the deoxy‐compounds (ionization normally occurs at the core nucleobase, N‐1), we were therefore able to semi‐quantitatively determine the amount of each modification. To scan for unexpected side‐products deriving from the different deoxy‐q, we performed in addition HRMS (Orbitrap Q Exactive). Taken together, with the exact mass and retention time, we can track any potential unnatural isomer,^[^ ^17^ ^]^ as for example, a possible product of non‐specific interaction of the enzymes QTMAN or QTGAL with our compounds.

To our delight we found that all three synthetic queuine derivatives **1–3** were indeed detected as the corresponding ribosylate forms **26–28**, showing that the queuine derivatives were processed by the TGT enzyme and properly incorporated into tRNA and hence reacted with a ribose (Figures 2 and S3). The cells treated with 2,3‐dideoxy‐q **3** converted the molecule into 2,3‐dideoxy‐Q **28** (Figures S3e and S4e). However, when we fed the mono‐deoxy‐queuine derivatives **1** and **2,** we also saw the signals for the man‐deoxy‐Q **29** (Figure 2c) and gal‐deoxy‐Q **30** (Figure 2c) derivatives. The analysis of the digested pool of small RNA after feeding with 3‐deoxy‐queuine **2** gave only signals for 3‐deoxy‐Q **27** and gal‐3‐deoxy‐Q **30** (Figure S4d), while feeding with the 2‐deoxy‐queuine **1** furnished signals for 2‐deoxy‐Q **26** and man‐2‐deoxy‐Q **29** only (Figure S4c), already indicating that the glycosylation reactions must be highly regiospecific. The 3‐OH group of Q is specifically loaded with only mannose, while the 2‐OH group of Q is exclusively loaded with only galactose; no other isomer was ever detected (Figure S4a,d,e). Because we observe no unexpected products by HRMS, we conclude that feeding, tRNA incorporation, and sugar loading proceed robustly, allowing us to next investigate the regioselectivity. For the next experiments, we isolated the individual Q‐containing tRNA with biotinylated oligodeoxynucleotide probes (bODN) complementary to tRNA^His^, tRNA^Asn^, tRNA^Tyr^, and tRNA^Asp^. Next, the Q content of these isolated tRNAs was analyzed using UHPLC‐HRMS. The experimental layout and the data are depicted in Figures 3 and S3.

**Figure 3:**
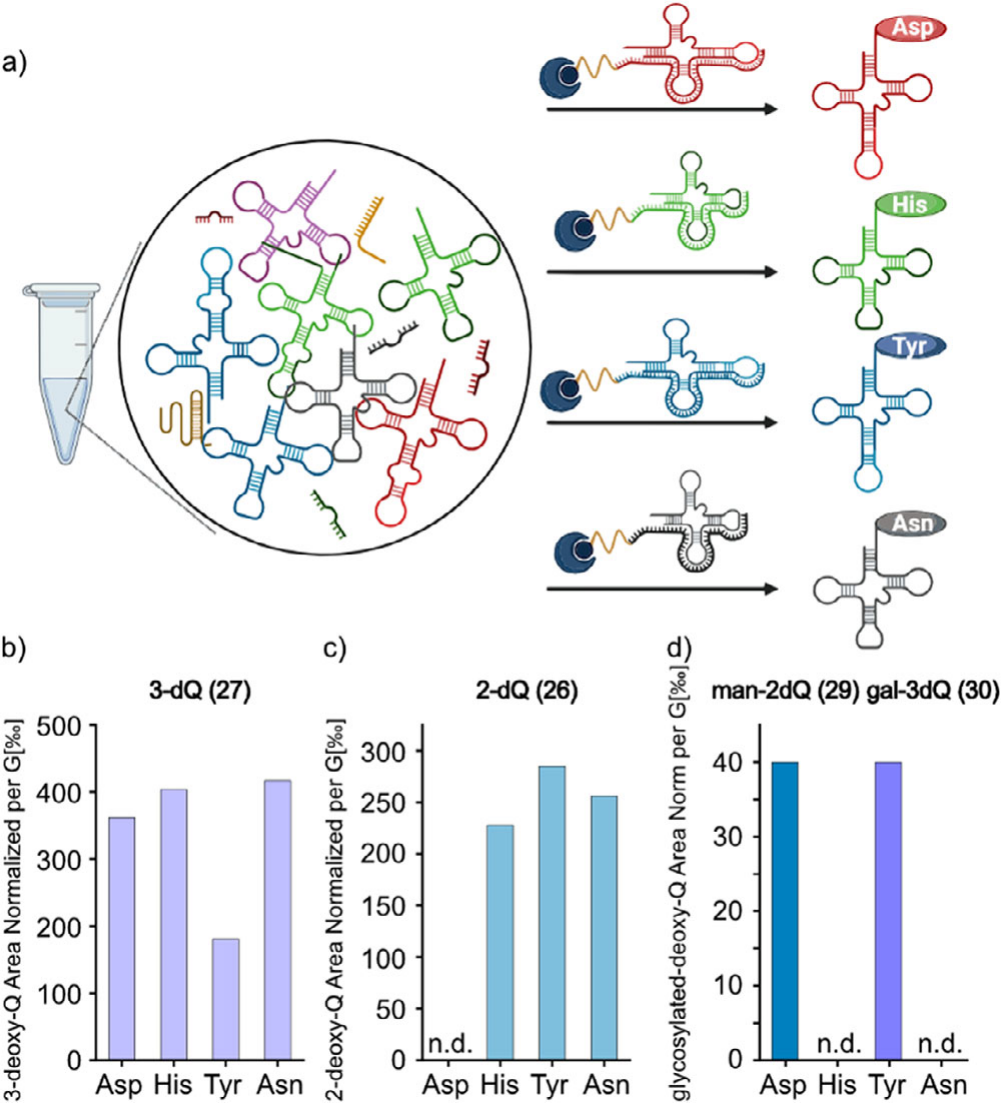
a) Scheme of the fishing strategy to isolate specific tRNA in a small RNA pool using a complementary biotinylated oligomer. In a small RNA pool, one sequence‐specific complementary biotinylated oligonucleotide is added, and products are hybridized and fished on magnetic beads. After washing, a purified single tRNA is eluted. b) Distribution of 3‐deoxy‐Q in the Q‐tRNAs. Similar 3‐deoxy‐Q levels are present on all Q‐tRNAs except tRNA^Tyr^ due to partial glycosylation shown in d). c) Distribution of 2‐deoxy‐Q in the Q‐tRNA. Similar 2‐deoxyQ levels are present on three of the Q‐tRNA and are non‐existent on tRNA^Asp^ due to complete glycosylation shown in d). d) Selective glycosylation of the corresponding Q‐tRNA. man‐2‐deoxy‐Q on tRNA^Asp^ and gal‐3‐deoxy‐Q on tRNA^Tyr^. No glycosylation was found in the tRNAs His and Asn. No mannose was found on tRNA^Tyr^ in the form of man‐3‐deoxy‐Q, and no galactose was found on tRNA^Asp^ in the form of gal‐2‐deoxyQ. All the data hereabove are from cell samples collected after 24 h of treatment with 20 µM for each molecule. The result of HRMS analysis and represented as their area under the curve, normalized per the ion count of G [‰].

After feeding 2,3‐dideoxy‐q **3**, we were able to detect the appearance of 2,3‐dideoxy‐Q **28** in all four Q‐tRNA, showing no clear substrate discrimination by the TGT enzyme when hydroxyl‐groups are missing (Figure S3f). The feeding of 3‐deoxy‐q **2** and 2‐deoxy‐q **1** confirmed the tolerance of the TGT enzyme. Additionally, the proportion of the deoxy‐queuosines **1–2** converted into gal‐3‐dQ **30** and man‐2‐dQ **29** (Figure S2b) are following the trend of the queuosine convertion into the glycosylated version. Indeed, non‐glycosylated Q is the most abundant product, followed by manQ. Finally, galQ is found only in small amounts (Figure S2c,d). More importantly, the Q in tRNA^Asp^ that contains 2‐deoxy‐Q (**Q** and **1**) is fully converted into the corresponding mannosylated version. This is different compared to the galactosylated Q, which is galactosylated in tRNA^Tyr^ with 3‐deoxy **(Q** and **2**) only partially. We observed only partial modification of tRNA^Tyr^ also previously in cultures grown in full medium^[^ ^5^ ^]^ and thereby believe that the observed low modification yield is not caused by a reduced substrate affinity of QTGAL toward the deoxy‐compound, but rather a natural yet unknown phenomenon. Importantly, we did not detect any mannosylated 3‐dQ or galactosylated 2‐dQ. From this data we can conclude that both enzymes, QTMAN and QTGAL, are remarkably selective for their respective substrate hydroxyl group. Thus, they are basically ignoring the “wrong” hydroxyl function.

In summary, we synthesized a number of deoxy‐queuine derivatives and showed that these synthetic building blocks are incorporated into the anticodon loops of tRNA^His^, tRNA^Asn^, tRNA^Tyr^, and tRNA^Asp^ at the typical wobble Q‐positions. We show that the galactosylating and mannosylating enzymes, which are responsible for the formation of manQ in tRNA^Asp^ and galQ in tRNA^Tyr^, are highly specific and selective. They accept either only the allyl‐ or only the homoallyl‐hydroxyl group for the corresponding reaction. Moreover, the absence of wrongly glycosylated Q indicates that glycosylated queuines, potentially released from the queuosine salvage pathway,^[^ ^19^ ^]^ are not unspecifically reincorporated into tRNA. Another interesting observation is that while the mannosylating enzyme fully converts all 2‐deoxy‐Qs into the man‐2‐deoxy‐Q, the galactosylating enzyme creates a mixture of 3‐deoxy‐Q and gal‐3‐deoxy‐Q containing tRNA^Tyr^. This could indicate the presence of a regulatory system in which the galactosylated and non‐galactosylated tRNA^Tyr^ are used for specific biological purposes. Given the involvement of queuosine in the biosynthesis of tyrosine,^[^ ^20^ ^]^ one may speculate about a regulatory function of the galactosylation. Further investigation is clearly needed. This finding, together with the high specificity and selectivity by which galQ and manQ are manufactured, suggests that they have distinct biological functions beyond just wobble‐decoding.

## Conflict of Interests

The authors declare no conflict of interest.

## Supporting information



Supporting Information

## Data Availability

The data that support the findings of this study are available in the Supporting Information of this article.
