# Supplementary material for: Selective Chemical Manipulation of Mannosyl‐ and Galactosyl‐Queuosine in tRNAs of Living Cells
Source: Angew Chem Int Ed Engl. 2025 Sep 3;64(42):e202508499. doi: 10.1002/anie.202508499 (PMC12518689; doi:10.1002/anie.202508499)
Supplement: Supplementary file 1 — Supporting Information [file ANIE-64-e202508499-s001.pdf]

Supporting Information

Selective Chemical Manipulation of Mannosyl- and Galactosyl-Queuosine in tRNAs of Living Cells

Alexander Pichler#, Elsa Peev#, Qingyi Ge, Ghofrane Ben Helal, Matthias Heiss, Stylianos Xefteris, Markus Müller and Thomas Carell*

[a] M.Sc. A. Pichler, M.Sc. E. Peev, M.Sc. Q. Ge, M.Sc. G. Ben Helal, Dr. Matthias Heiss, B. Sc. S. Xefteris, Dr. M. Müller, Prof. T. Carell
Ludwig Maximilians Universität München
Center for Nucleic Acid Therapies at the Department of Chemistry
Institute for Chemical Epigenetics
Butenandtstr. 5-13, 81377 München, Germany
Email: Thomas.carell@lmu.de

# These authors contributed equally

Table of Contents

1. Supplementary Figures 3

2. Material and Methods 9

3. Cell Culture and Biological Assays 10

3.1 Cell culture 10

3.2 RNAs extraction and preparation 10

3.3 Analytical Methods 12

4. Experimental procedures and characterization 14

4.1 Synthesis of the amino-cyclopentenol rings 14

(±)-Cyclopentadiene monoepoxide ((±)-4) 14

(1*S*,2*S*)- 2-aminocyclopent-3-en-1-ol ((±)-5) 14

t-Butyl ((1*S*,5*S*)-5-hydroxycyclopent-2-en-1-yl)carbamate ((±)-6) 15

t-Butyl ((1*S*,5*S*)-5-hydroxycyclopent-2-en-1-yl)carbamate ((+)-7) 15

t-butyl ((1S,5S)-5-((triisopropylsilyl)oxy)cyclopent-2-en-1-yl)carbamate (8) 16

(1S,5S)-5-((triisopropylsilyl)oxy)cyclopent-2-en-1-amine (9) 16

(R)-3,3,3-trifluoro-2-methoxy-2-phenyl-N-((1S,5S)-5-((triisopropylsilyl)oxy)cyclopent-2-en-1-yl)propenamide (10) 17

4-hydroxycyclopent-2-en-1-one (15) 17

(1R,3S)-cyclopent-4-ene-1,3-diol (16) 18

(1S,4R)-4-hydroxycyclopent-2-en-1-yl acetate (17) 18

(1S,4R)-4-((triisopropylsilyl)oxy)cyclopent-2-en-1-yl acetate (18) 19

(1S,4R)-4-((triisopropylsilyl)oxy)cyclopent-2-en-1-ol (19) 19

2-((1R,4R)-4-((triisopropylsilyl)oxy)cyclopent-2-en-1-yl)isoindoline-1,3-dione (20) 20

(1R,4R)-4-((triisopropylsilyl)oxy)cyclopent-2-en-1-amine (13) 20

(R)-3,3,3-trifluoro-2-methoxy-2-phenyl-N-((1S,5S)-5-((tri-tert-butylsilyl)oxy)cyclopent-2-en-1-yl)propenamide (21) 21

Barton-McCombie deoxygenation: O-((1S,2S)-2-((tert-butoxycarbonyl)amino)cyclopent-3-en-1-yl) 1H-imidazole-1-carbothioate intermediate and tert-butyl (R)-cyclopent-2-en-1-ylcarbamate (11) 21

(R)-cyclopent-2-en-1-amine (12) 22

4.2 Synthesis of deoxyQ (1, 2 and 3) 23

t-Butyl 2-(bis(*t*-butoxycarbonyl)amino)-4-(*t*-butoxy)-5-formyl-7H-pyrrolo[2,3-d]pyrimidine-7-carboxylate (22) 23

General procedure for the synthesis of deoxy-queuine via reductive amination and subsequent deprotection of the molecule (23, 24, 25) 24

2-amino-5-((((1R,4R)-4-hydroxycyclopent-2-en-1-yl)amino)methyl)-3,7-dihydro-4H-pyrrolo[2,3-d]pyrimidin-4-one (1) 25

2-amino-5-((((1*S*,5*S*)-5-hydroxycyclopent-2-en-1-yl)amino)methyl)-3,7-dihydro-4H-pyrrolo[2,3-d]pyrimidin-4-one (2) 25

(R)-2-amino-5-((cyclopent-2-en-1-ylamino)methyl)-3,7-dihydro-4H-pyrrolo[2,3-d]pyrimidin-4-one (3) 26

5. NMR-spectra of important compounds 27

6. References 49

1. Supplementary Figures

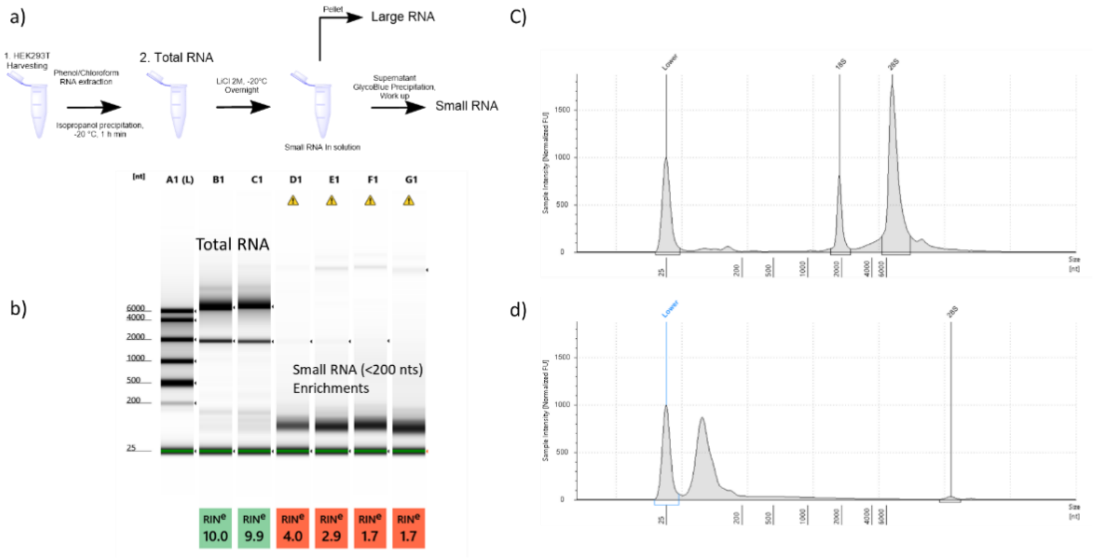

Figure S1. Small RNA Enrichment. a) General Scheme of enrichment of small RNA using a 2M LiCl precipitation method. b) Gel image of the of the total RNA pool (columns B1, C1) and the enriched small RNA fraction (columns D1, E1, F1, G1). The small RNA fractions were further used for tRNA enrichment or small RNA pool analysis. RNA-ladder size is displayed next to the corresponding column (A1). c) Representative electropherogram of the total RNA pool. d) Representative electropherogram of the enriched small RNA pool (<200 nts).

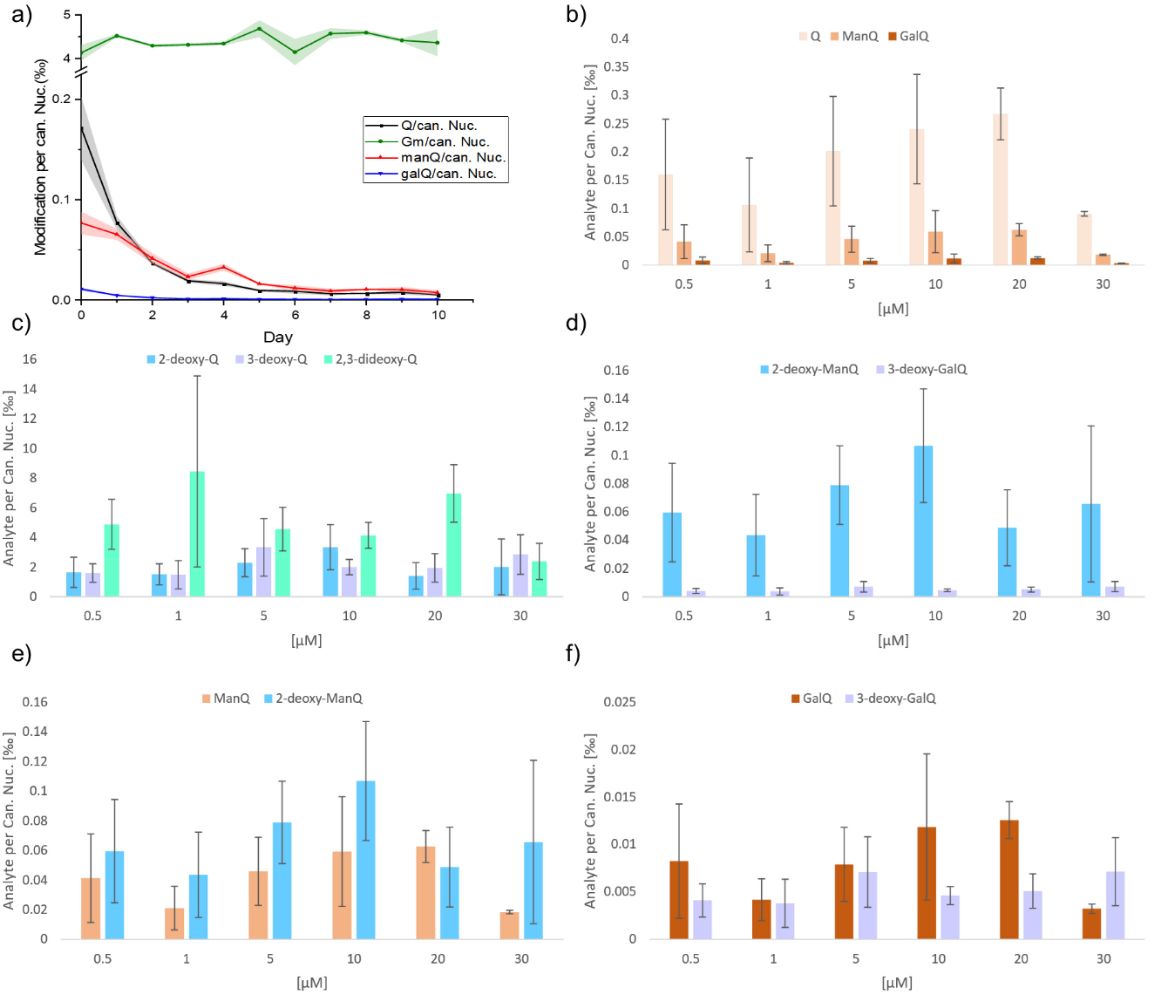

Figure S2 Q-starvation and replenishment. **a)** Modification content in small RNA after Q-starving of HEK293T in Dia-FBS over 10 days. Cells were considered Q-free at day 7. Any further treatment was started after day 10. As reference, 2'-O-methyl-G (Gm) is represented in green, Q is black and manQ and galQ are red and blue, respectively. **b-d)** Levels of Q derivatives in small RNA measured 24 h after replenishing the medium with indicated concentrations of q. **b)** Feeding of natural q (µM): Q (light orange), manQ (orange) and galQ (dark orange). **c)** Feeding of deoxy-q: 2-deoxy-Q (blue), 3-deoxy-Q (purple) and 2,3-dideoxy-Q (green) **d)** Appearance of the glycosylation products: gal-3-deoxy-Q (purple) and man-2-deoxy-Q (blue). **e-f)** Comparison of the Q glycosylation levels between natural q and deoxy-q feedings displayed in part b) and d) of the same figure. All the data in this panel are UHPLC-QQQ results of the average modification number in digested small RNA. Quantification is based on using a heavy labelled internal standard (ISTD) of Queuosine. Therefore, absolute values might be distorted, the relative comparison between samples is however accurate. Values are given as average number of Q or modified-Q molecules amount normalized by the average sum of canonical nucleoside. Error bars represent the standard deviation of three biological replicates.

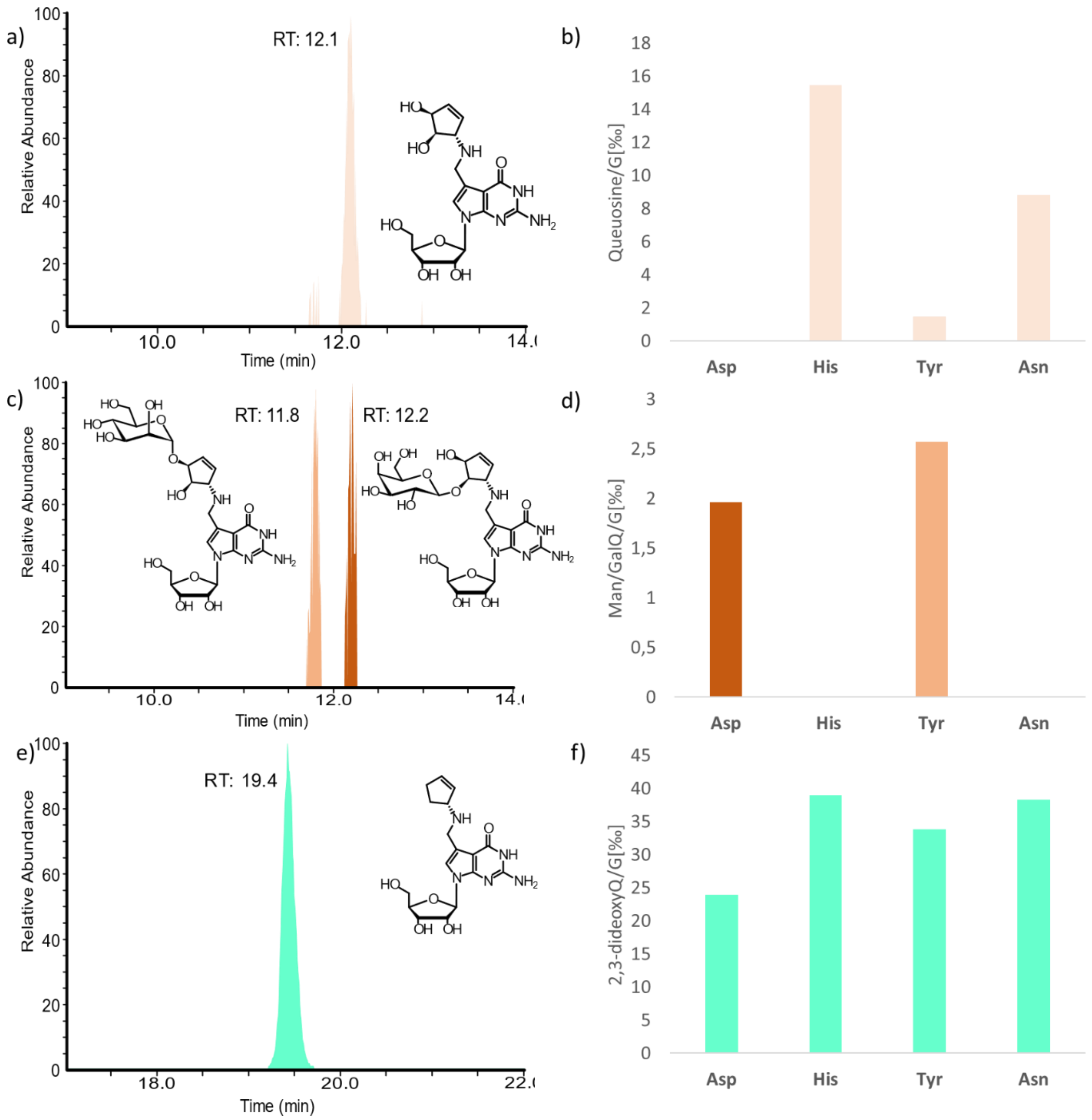

Figure S3. Q content in purified tRNA a) UHPLC-HRMS extracted signal of Queuosine, RT 12.1, [M+1] = 410.1670 b) Relative queuosine amount in the four Q-tRNA. c) UHPLC-HRMS extracted signal of manQ (orange, RT 11.8 minutes) and galQ (dark orange, RT 12.2 minutes), [M+1] = 572.2198. d) Relative glyco-deoxy-Qs amount in the four Q-tRNA. e) UHPLC-HRMS extracted signal of 2,3-dideoxy-Q (green, RT 19.4 minutes), [M+1] =378.1772. d) Relative 2,3-dideoxy-Q amount in the four Q-tRNA. All the data in this figure are from UHPLC-HRMS analysis. The results are qualitative and the bar graphs (b, d, and f) represent the area under the curve (AUC) of each analyte normalized by the ion count of G. Therefore, absolute values are meaningless, only the general trend of each unique molecule in different samples matter.

a)

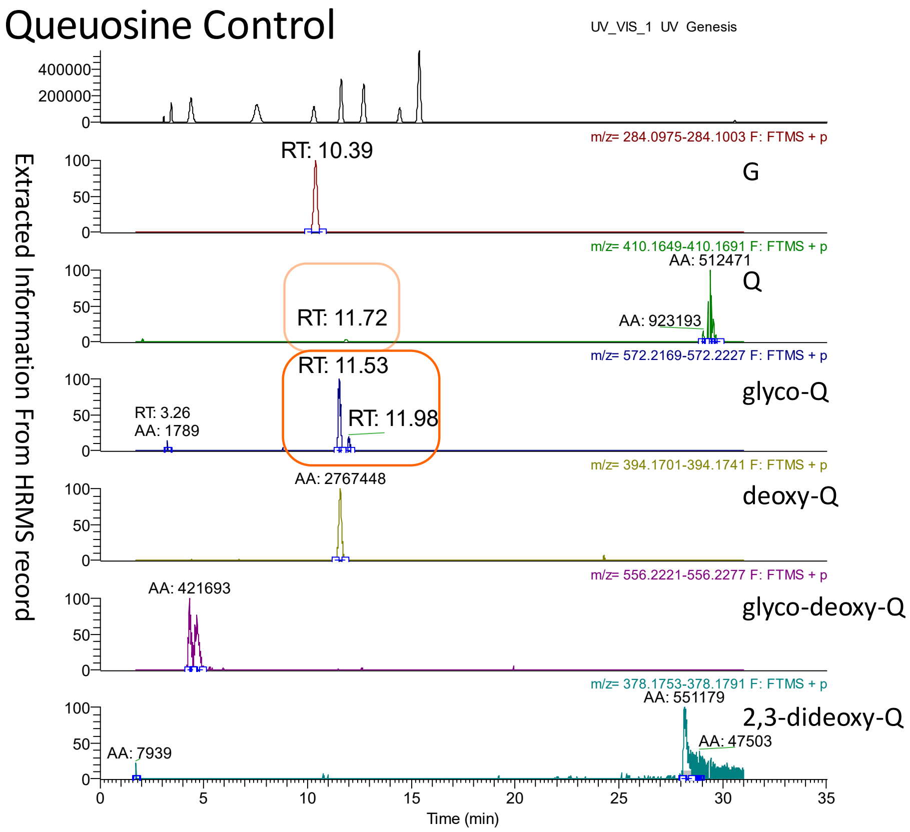
Queuosine Control
UV_VIS_1 UV Genesis
400000
200000
0
m/z= 284.0975-284.1003 F: FTMS + p
RT: 10.39
G
m/z= 410.1649-410.1691 F: FTMS + p
AA: 512471
Q
RT: 11.72
AA: 923193
m/z= 572.2169-572.2227 F: FTMS + p
RT: 11.53
glyco-Q
RT: 3.26
AA: 1789
RT: 11.98
m/z= 394.1701-394.1741 F: FTMS + p
AA: 2767448
deoxy-Q
m/z= 556.2221-556.2277 F: FTMS + p
AA: 421693
glyco-deoxy-Q
m/z= 378.1753-378.1791 F: FTMS + p
AA: 551179
2,3-dideoxy-Q
AA: 7939
AA: 47503
Extracted Information From HRMS record
Time (min)

b)

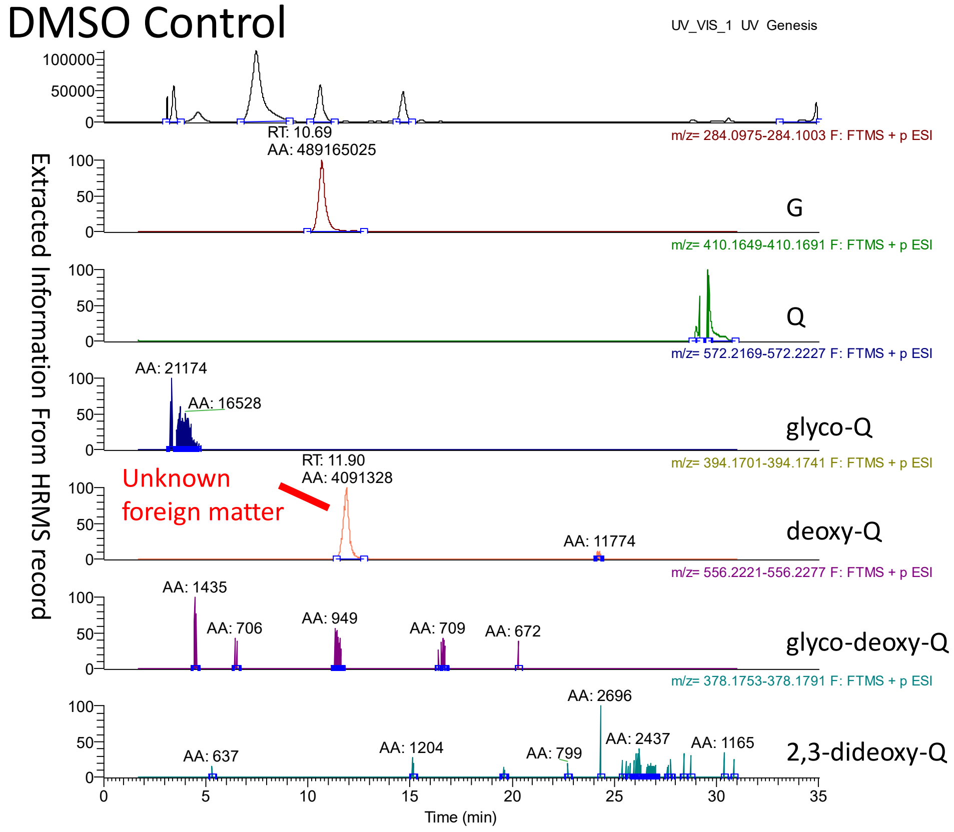
DMSO Control
UV_VIS_1 UV Genesis
100000
50000
0
RT: 10.69
AA: 489165025
m/z= 284.0975-284.1003 F: FTMS + p ESI
G
m/z= 410.1649-410.1691 F: FTMS + p ESI
Q
m/z= 572.2169-572.2227 F: FTMS + p ESI
AA: 21174
AA: 16528
glyco-Q
RT: 11.90
AA: 4091328
m/z= 394.1701-394.1741 F: FTMS + p ESI
Unknown foreign matter
AA: 11774
deoxy-Q
m/z= 556.2221-556.2277 F: FTMS + p ESI
AA: 1435
AA: 706
AA: 949
AA: 709
AA: 672
glyco-deoxy-Q
m/z= 378.1753-378.1791 F: FTMS + p ESI
AA: 2696
AA: 637
AA: 1204
AA: 799
AA: 2437
AA: 1165
2,3-dideoxy-Q
Extracted Information From HRMS record
Time (min)

c)

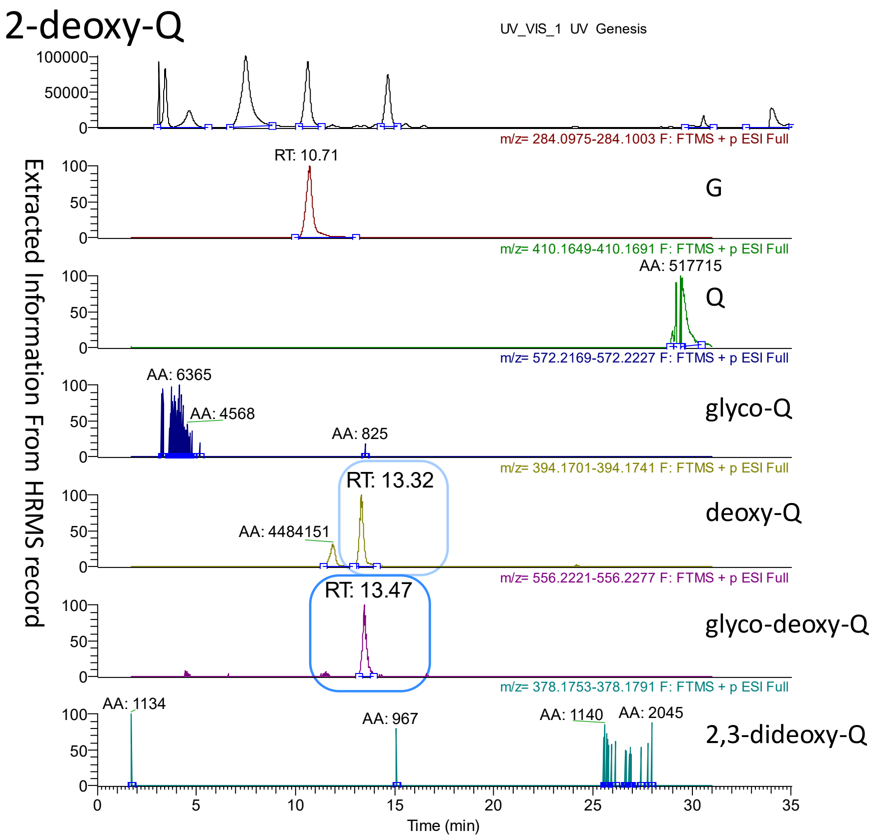

2-deoxy-Q
UV_VIS_1 UV Genesis
m/z= 284.0975-284.1003 F: FTMS + p ESI Full
RT: 10.71
G
m/z= 410.1649-410.1691 F: FTMS + p ESI Full
AA: 517715
Q
m/z= 572.2169-572.2227 F: FTMS + p ESI Full
AA: 6365
AA: 4568
AA: 825
glyco-Q
m/z= 394.1701-394.1741 F: FTMS + p ESI Full
RT: 13.32
AA: 4484151
deoxy-Q
m/z= 556.2221-556.2277 F: FTMS + p ESI Full
RT: 13.47
glyco-deoxy-Q
m/z= 378.1753-378.1791 F: FTMS + p ESI Full
AA: 1134
AA: 967
AA: 1140
AA: 2045
2,3-dideoxy-Q
Extracted Information From HRMS record
Time (min)

d)

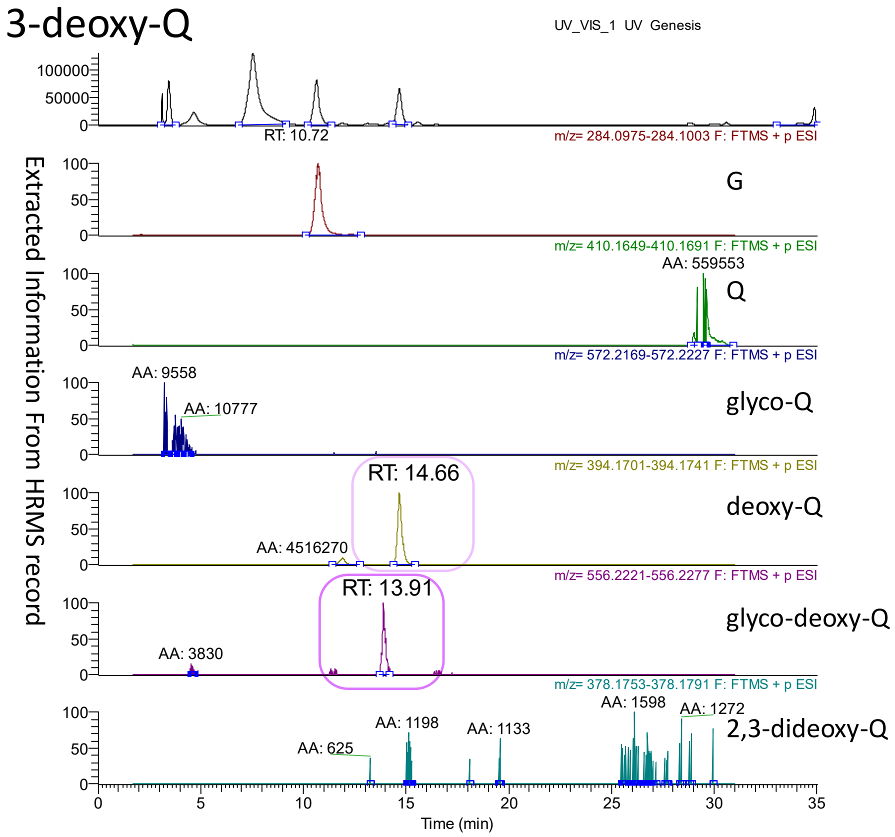

3-deoxy-Q
UV_VIS_1 UV Genesis
RT: 10.72
m/z= 284.0975-284.1003 F: FTMS + p ESI
G
m/z= 410.1649-410.1691 F: FTMS + p ESI
AA: 559553
Q
m/z= 572.2169-572.2227 F: FTMS + p ESI
AA: 9558
AA: 10777
glyco-Q
m/z= 394.1701-394.1741 F: FTMS + p ESI
RT: 14.66
AA: 4516270
deoxy-Q
m/z= 556.2221-556.2277 F: FTMS + p ESI
RT: 13.91
AA: 3830
glyco-deoxy-Q
m/z= 378.1753-378.1791 F: FTMS + p ESI
AA: 1598
AA: 1272
AA: 1198
AA: 1133
AA: 625
2,3-dideoxy-Q
Extracted Information From HRMS record
Time (min)

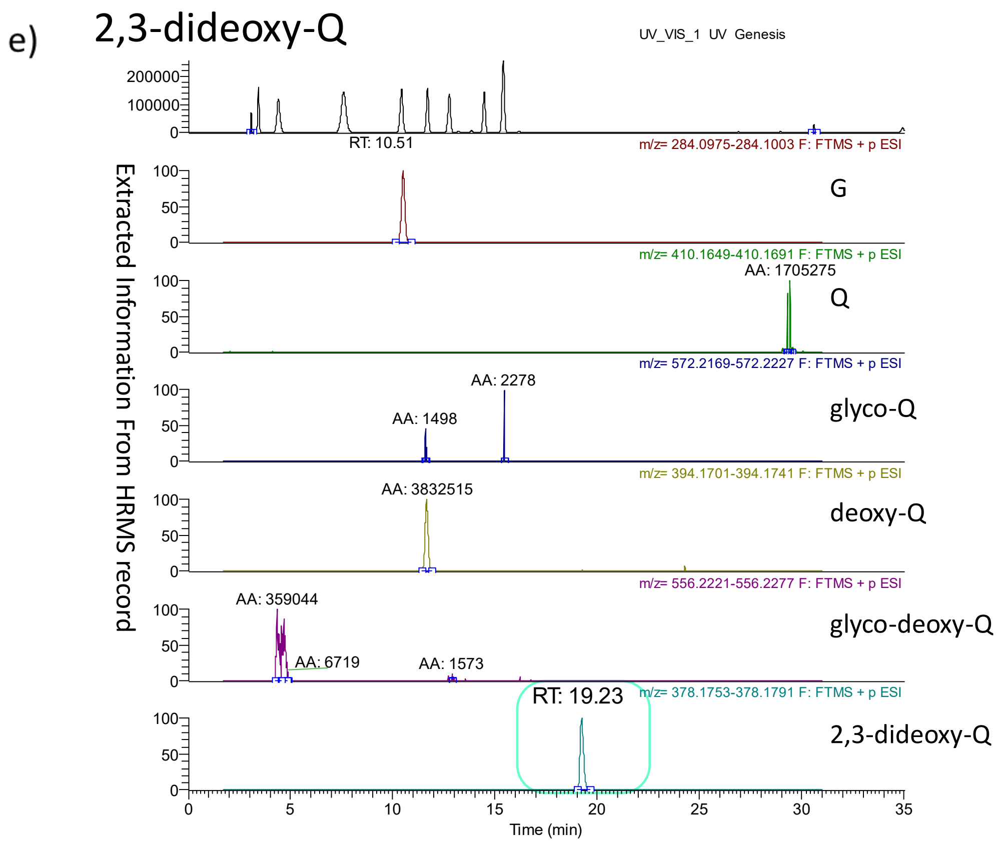

Figure S4. **a)** UHPLC-HRMS result of Q, the positive control. From top to bottom, the UV profile (260nm), the extracted G signal (elution at 10.4 min), the extracted Q signal (elution at 12.1 min), the extracted glyco-Q signals (positive at 11.5 and 12 min), the extracted deoxy-Q signal (negative at 13.3 min and 14.7), the extracted glyco-deoxy-Q signal (negative at 13.5 min and 13.9) and the extracted 2,3-dideoxy-Q signal (negative at 19.2 min). **b)** UHPLC-HRMS result of the negative control DMSO. From top to bottom, the UV_{260nm} profile, the extracted G signal (positive at 10.7 min), the extracted Q signal (negative at 12.1 min), the extracted glyco-Q signals (negative at 11.5 and 12 min), the extracted deoxy-Q signal (negative at 13.3 min and 14.7), the extracted glyco-deoxy-Q signal (negative at 13.5 min and 13.9) and the extracted 2,3-dideoxy-Q signal (negative at 19.2 min). **c)** UHPLC-HRMS result of the sample 2-deoxy-Q. From top to bottom, the UV_{260nm} profile, the extracted G signal (positive at 10.7 min), the extracted Q signal (negative at 12.1 min), the extracted glyco-Q signals (negative at 11.5 and 12 min), the extracted deoxy-Q signal (positive at 13.3 min and negative at 14.7), the extracted glyco-deoxy-Q signal (positive at 13.5 min and negative at 13.9) and the extracted 2,3-dideoxy-Q signal (negative at 19.2 min). **d)** UHPLC-HRMS result of the sample 3-deoxy-Q. From top to Bottom, the UV_{260nm} profile, the extracted G signal (positive at 10.4 min), the extracted Q signal (negative at 12.1 min), the extracted glyco-Q signals (negative at 11.5 and 12 min), the extracted deoxy-Q signal (negative at 13.3 min and positive at 14.7), the extracted glyco-deoxy-Q signal (negative at 13.5 min and positive at 13.9) and the extracted 2,3-dideoxy-Q signal (negative at 19.2 min). **e)** UHPLC-HRMS of the negative control DMSO. From top to bottom, the UV_{260nm} profile, the extracted G signal (positive at 10.5 min), the extracted Q signal (negative at 12.1 min), the extracted glyco-Q signals (negative at 11.5 and 12 min), the extracted deoxy-Q signal (negative at 13.3 min and 14.7), the extracted glyco-deoxy-Q signal (negative at 13.5 min and 13.9) and the extracted 2,3-dideoxy-Q signal (positive at 19.2 min). All mass above are presented as [M + 1].

2. Material and Methods

General Chemistry Material. Chemicals were purchased from Sigma-Aldrich, TCI, Fluka, ABCR, Carbosynth or Acros Organics and used with exceptions without further purification. Solvents of reagent grade were purified by distillation. Reactions and column chromatography fractions were monitored by thin-layer chromatography (TLC) on silica gel F254TLC plates from Merck KGaA. Flash column chromatography was performed on Geduran® Si60 (40-63 µm) silica gel from Merck KGaA, applying nitrogen pressure. Chemical transformations were conducted under nitrogen or argon atmosphere in oven-dried glassware unless otherwise specified. NMR spectra were recorded on *Bruker Ascend 500* or on *Bruker Avance III HD 800*. ^{1}H-NMR shifts were calibrated to the residual solvent resonances: CD_2Cl_2 (5.32 ppm), CD_3OD (3.31 ppm) and D_2O (4.79 ppm). ^{13}C-NMR shifts were calibrated to the residual solvent resonances: CD_2Cl_2 (53.84 ppm) and CD_3OD (49.00 ppm). All NMR spectra were analysed using the program MestReNova 15.0.0 from Mestrelab Research S.L. High resolution mass spectra (HRMS) were measured by the analytical section of the Department of Chemistry of the Ludwigs-Maximilians-Universität München on a MAT 95 (EI) from Thermo Finnigan GmbH, Thermo Finnigan LTQ FT Ultra Fourier Transform Ion Cyclotron Resonance or Thermo Finnigan LTQ Orbitrap XL mass spectrometer. IR spectra were recorded on a *Shimadzu IRSpirit* spectrometer. Substances were applied as a film or directly as solids on the ATR unit. Analytical RP-HPLC was performed on an Agilent 1260 Infinity II Analytical LC System equipped with the column Nucleodur 100-3 C18ec from Macherey-Nagel applying eluent flow of 0.5 mL/min. Preparative RP-HPLC was performed on an Agilent 1260 Infinity II Manual Preparative LC System equipped with the column Nucleodur 100-5 C18ec from Macherey-Nagel. A flowrate of 5 mL/min was applied.

3. Cell Culture and Biological Assays

General Materials. All commercially available reagents or media are used without purification if there is no other indication and stored according to the product sheet. All the reagents were purchased from Sigma Aldrich and Thermo Fisher Scientific, unless otherwise noted. All solutions and buffers were prepared with ultra-pure type I water (OmniaPure, Stakpure)) at room temperature. pH values were adjusted by using a pH meter (Mettler Toledo). The queuine and the deoxy-queuine compounds were synthesized in-house

3.1 Cell culture

Mycoplasma Contamination Test. Cells were routinely tested for contamination with Mycoplasma. The mycoplasma contamination test was carried out using Mycoplasma PCR Detection Kit (abm). The amplification products were analysed by agarose electrophoresis. 1% Agarose gel done with tris-acetate-EDTA (TAE). The result was then imaged, using GelGreen and recorded with Amersham Imager 680 (Blot und Gel, Cytvia)

Culture of HEK 293T Cells. HEK293T cells are cultivated at 37 °C in water-saturated (heat, sterilized and filtered), CO_2-enriched (5%) atmosphere. Cells were grown in High Glucose Dulbecco's Modified Eagle Medium (DMEM) supplemented with 10% (v/v) Heat Inactivated Fetal Bovine Serum (HI FBS, Gibco, A5256801), 1% (v/v) L-alanyl-L-glutamine, 1% (v/v) Penicillin-Streptomycin (Pen-Strep)), hereafter named DMEM-Full. After a minimum of 3 passages, cells were passed into DMEM reconstituted similarly with dialyzed FBS (Seraglob, S40500DIA), hereafter as DMEM-Dia. When reaching a confluency of 80 – 90%, HEK293T cells were routinely passaged. In each passage the washing step with Dulbecco's Phosphate Buffered Saline and with TrypLE (Life Technologies Corporation) as dissociation reagent. **HEK293T Queuosine-Starving.** To deplete cells of natural occurring queuosine, HEK293T are cultured for a minimum of 10 days in DMEM-Dia before any further experiment.

Cell culture for Q-depletion of HEK 293T cells. HEK 293T cells were initially seeded in DMEM medium with normal FBS. After a minimum of 3 passages after thawing, the medium was switched to the DMEM-Dia. Over the following ten days, cells were routinely passaged every two to three days into fresh dialyzed FBS medium. Daily cell collections allowed for small RNA isolation by precipitation of large RNA with LiCl. These enriched small RNAs were then digested to nucleosides and analysed using quantitative LC-MS/MS (LC-QQQ-MS) to quantify the levels of Q and glycosylated Q at various time points during the Q depletion period.

Queuine and q-derivative feeding. Q-starved cells were divided in 5 groups. Each group were first fed with a concentration range from 0.5 µM to 30 µM of queuine, 2-deoxy-q, 3-deoxy-q, 2,3-dideoxy-q or DMSO as control. The saturation point of Q in tRNA was reached after 24h at 20 µM for q and q-derivatives. HEK293T cells maintained in DMEM-Full or feed with natural q were used as positive control according to the aim of the test. Harvested cells were further processed for small RNA enrichment or tRNA isolation as describe below.

3.2 RNAs extraction and preparation

RNA Extraction and Enrichment of Small RNAs. To harvest the cells, growing media was removed and cells are washed with DPBS. The cells are then lysed directly inside the plate using 1 mL of TRI-Reagent® per $1 \cdot 10^7$ cells. The solution was incubated at room temperature for 5 min, scraped and pipetted into clean tubes. The following steps were done according to the manufacturer's protocol. After the addition of 1/5 volume (Vol) of chloroform, the solution was vortexed thoroughly and incubated for 5 min at room temperature. The mixture was centrifuged at 8'000 *xg* for 8 minutes. The RNA-containing

aqueous phase was transferred into new reaction tubes and mixed with 1 volume of isopropanol. The mixture was incubated at -20 °C for 1 hour to overnight to precipitate all the total RNA pool. The mixture was centrifuged at 12’000 *xg* for 30 min at 4 °C. The supernatant was discarded, and the pellet was carefully washed with 75 % ethanol and centrifuged again at 12’000 *xg* for 5 min. The supernatant was discarded and the pellet was air-dried for 2-5 minutes. The pellet was resuspended in ultrapure water or in 2 M lithium chloride solution to enrich the small RNA < 200 nt. For the small RNA enrichment, the solution is placed at -20 °C for an hour to overnight. The mixture was centrifuged thereafter at 14’000 *xg* for 15 minutes at 4 °C. The supernatant containing the small RNA was transferred in a new reaction tube and was mixed with GlycoBlue™ Coprecipitant, 1/10 volume 5 M ammonium acetate (NH_4OAc) and 2.5 volumes absolute EtOH. The mixture was then incubated at -20 °C for 1 hour to overnight. It was then centrifuged at 12’000 *xg* for 30 min at 4 °C. The supernatant was discarded, and the pellet was carefully washed with 75% EtOH. After centrifugation at 12’000 *xg* for 5 minutes at 4 °C, the supernatant was discarded, and the pellet was air-dried for 2-5 minutes. The small RNAs were resuspended in ultrapure water. The RNA concentration was determined photometrically at a wavelength of 260 nm and 280 nm on a UV/Vis nanophotometer (Implen).

tRNA Isoacceptor purification. Isolation of specific Q-tRNA ($tRNA^{Tyr}$, $tRNA^{Asp}$, $tRNA^{His}$, and $tRNA^{Asn}$) was done using an enrichment strategy with complementary biotinylated-oligodeoxynucleotides (bODN). The method was adapted from R. Hauenschild et al.[1] Enriched small RNA and bODN were mixed in a ratio of 1:1 in Saline Sodium Citrate (SSC) buffer (NaCl 750 mM, trisodiumcitrate 75 mM, pH 7.0). Hybridization of RNA was performed in a thermocycler under the following conditions: 90 °C for 3 minutes, 65 °C for 10 minutes and -1 °C/minute to 10 °C. Hybridised products were mixed with Dynabeads™ MyOne™ Streptavidin T1 after their equilibration (3x washing with B&W buffer (5 mM Tris-HCl (pH = 7.5), 0.5 mM EDTA, 1 M NaCl) and 1x washing with SSC buffer and resuspended in SSC buffer). The mixture was incubated in a Thermomixer at 600 rpm for 1 hour at 22 °C. The unbound small RNAs were removed by consecutive washing with diluted SSC buffer (1x with dilution of 5x and 3x with dilution of 50x). The purified target tRNA was removed from streptavidin beads by incubating at 90 °C for 2 min in ultrapure water (2x 15 µL) and the collection of supernatants on a magnetic rack. The specific Q-tRNA was transferred into clean tube and processed further to prepare for the analysis.

$\mathrm{tRNA}^{\mathrm{Asp}}_{\mathrm{GUC}}$ 5’-[Biotin]TGGCTCCCCGTCGGGGAATTGAACCCCGGTCTCCCGCGTGACAGGCGGGGATACTAACCACTATACTAACGAGG AAAA-3’

$\mathrm{tRNA}^{\mathrm{His}}_{\mathrm{GUG}}$ 5’-[Biotin]TGCCGTCACTCGGATTCGAACCGAGGTTGCTG-3’

$\mathrm{tRNA}^{\mathrm{Asn}}_{\mathrm{GUU}}$ 5’-[Biotin]AAATGGCGTCCCTGGGTGGGCTCGAACCACCAACCTTTTCGGTTAACAGCC-3’

$\mathrm{tRNA}^{\mathrm{Tyr}}_{\mathrm{GUA}}$ 5’-[Biotin]AAATGGTCCTTCGAGCCGGAATCGATCCAGCGA-3’

RNA, Small RNA and Fished-tRNA digestion. As reported previously by Heiss *et al.*[2], samples are digested in tris(hydroxymethyl)aminomethane (Tris) (5 mM, pH 8) with $MgCl_2$ (1 mM), benzonase (0.1 to 2 U), alkaline phosphatase (CIP) (0,1 to 2 U), phosphodiesterase (PDE1, Worthingtons Biochemical Corporation) (0.01 to 0.2 U), pentostatin (0.05 to 1 µg), tetrahydrouridine (THU) (0.25 to 5 µg) and butylated hydroxytoluene (BHT) (0.5 to 10 µM). The digestion reaction is incubated for 2 h at 37 °C. At the end of the reaction, 1:3 eq. of volume of the HRMS or UHPLC-QQQ aqueous running buffer are added to the mixture. It was then filtered through 96-well filter plates (AcroPrep Advance 350 0.2 µm Omega, PALL Corporation, New York, USA) at 3200 *xg* at 10 °C for 50 min.

3.3 Analytical Methods

Quality control of small RNAs enrichment. Before applying the isoacceptor fishing strategy, the quality of the enriched small RNAs was assessed. This step is crucial to avoid non-specific RNA binding during hybridization (see above), particularly with sequences that are similar to the target RNAs and are abundant in total RNA. The quality of the small RNAs enrichment was checked by using high sensitivity RNA ScreenTape analysis (HS RNA) with a TapeStation instrument (Agilent) as displayed in Figure S1 below. The sample preparation and assay were done according to the manufacturer recommendations. The samples were loaded into the TapeStation instrument and the results of integrity of the total RNAs were analysed by Agilent 4150 Bioanalyzer system based on the RNA Integrity Number (RIN). Small RNA-enriched samples contain primarily shorter RNA molecules and often contain no or very little intact long-chain rRNA, which result in lower RIN numbers.

UHPLC-HRMS, QExactive Orbitrap. For the HRMS, all solvents were LC-MS grade. This MS system combines high-performance liquid chromatography (LC) with HRMS *via* an Orbitrap mass analyser. It was used for qualitative small molecules exact mass (MS1) analysis of RNA samples according to the method reported previously.[3] The digested and filtered RNA samples were transferred into MS vials for HRMS measurement. For the HRMS analysis, the Thermo Scientific Vanquish system was integrated with a Thermo Scientific QExactive HF mass spectrometer. Ionization was achieved using a HESI source, and ions were monitored in positive polarity mode over a scan range of m/z 200-2000, reaching a resolution of 120’000, with an Automatic Gain Control (AGC) target of $3 \cdot 10^6$, and a maximum injection time (IT) of 200 ms. Settings for the HESI Tune: capillary temperature of 320 °C, sheath gas flow rate of 20 au, auxiliary gas flow rate of 4 au, sweep gas flow rate of 0 au, spray voltage of 3.5 kV, S-lens RF level of 30, and auxiliary gas heater set at 55 °C. Chromatographic separation was performed using an Uptisphere C18-HDO column (3.0 µm, 150 × 2.1 mm from Interchim, UP3HDO-150/021) maintained at 35 °C. For the mobile phase, buffer A consisted of a 5 mM NH_4OAc solution adjusted to pH 4.9 using 200 µL/L of glacial acetic acid, and buffer B was a 2 mM ammonium formate (NH_4COOH) solution in 80% acetonitrile (MeCN, Roth, Ultra LC-MS grade, with purity ≥99.98 %). The flow rate of the elution buffer was maintained at 0.20 mL/min. The gradient started at 100 % solvent A for 3 minutes, followed by an increase of solvent B to 15% over 15 min (3 min – 18 min) then to 60% B within the following 7 minutes (18 min – 25 min) and further to 100% B within 1 minute (25 min – 26 min). 100% B was maintained for 5 minutes (26 min – 31 min) before returning to 100 % solvent A in 1 minute and a 3 minutes re-equilibration period. Sample volumes ranging from 10 – 25 µL were injected using the automatic sampler of the HPLC system and concurrently analysed *via* HRMS. Ion chromatograms were derived from the total ion current (TIC) chromatogram with a mass tolerance set at 5 ppm, and peak areas were integrated for sample comparison. The HRMS data were analysed using the Qual Browser tool within Xcalibur.

UHPLC-QQQ-MS, quantitative LC-MS/MS. For QQQ-LC-MS/MS, all solvents were LC-MS grade. An Agilent 1290 Infinity equipped with a variable wavelength detector (VWD) combined with an Agilent Technologies G6490 Triple Quadrupole LC-MS/MS system and electrospray ionization (ESI-MS, Agilent Jetstream) was used. Operating parameters: positive-ion mode, cell accelerator voltage of 5 V, N_2 gas temperature of 120 °C and N_2 gas flow of 11 L/min, sheath gas (N_2) temperature of 280 °C with a flow of 11 L/min, capillary voltage of 3000 V, nozzle voltage of 0 V and nebulizer at 60 psi, high-pressure RF at 100 V and low-pressure RF at 60 V. The instrument was operated in dynamic MRM mode. For separation, an Uptisphere C18-HDO column (3.0 µm, 150 × 2.1 mm from Interchim, UP3HDO-150/021) was used at 35 °C and a flow rate of 0.35 mL/min was used for all experiments in combination with a binary mobile phase of 5 mM NH_4OAc aqueous buffer A (pH 4.9) and organic buffer B (2 mM NH_4COOH in 80% MeCN). The gradient started at 100 % solvent A for 1.5 minutes, followed by an increase of solvent B to 10% over 5.5 min (1.5 min – 8.5 min) and further to 80% B within the following minute (8.5 min – 9.5 min). 80% B was maintained for 1.5 minute (9.5 – 12 minute) before returning to 100 % solvent A in 0.5 minute and a 2.2 minutes re-equilibration period. Of each sample, 100 ng were co-injected with 1 µL of stable isotope labelled internal standard (ISTD). The QQQ-MS data were analysed by the quantitative and qualitative MassHunter Software from Agilent (v B07.01) using the integrated calibration function. The calibration solutions ranged from 0.05 pmol to 100 pmol for each canonical nucleoside (A, C, G and U), from 0.01 to 20 pmol for pseudouridine and from 0.002 pmol to 5 pmol for the others modified nucleosides (Cm m^3C, m^5C, m^5U, Gm, m^1G, m^7G, $m^{2,2}G$, m^1A, m^6A, $m^{6,6}A$, t^6A, ct^6A, i^6A, ms^2i^6A, Q, galQ, manQ). The quantification of Q-derivatives was done by using a stable isotope labeled internal standard of Q. As we assume low ionization differences between the single derivatives semi-quantitative analysis was possible and the general trend accurately displayed.

4. Experimental procedures and characterization

4.1 Synthesis of the amino-cyclopentenol rings

(±)-Cyclopentadiene monoepoxide ((±)-4)

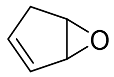

Freshly cracked cyclopentadiene (6.00 g, 90.8 mmol, 1.00 eq.) was dissolved in DCM (50 mL) and Na_2CO_3 (12.0 g, 113 mmol, 1.25 eq.) was added. The reaction mixture was cooled to 20 °C and a solution of NaOAc (246 mg, 3.00 mmol, 3.30 mol%) in peracetic acid (39% in acetic acid, 7.73 mL, 45.4 mmol, 0.50 eq.) was added over 40 min during which time the temperature was maintained at 20 °C by external cooling. The reaction mixture was allowed to come to r.t. and continued stirring for 1.5 h. The solid was filtered off and the filtrate was concentrated *in vacuo* at 22 °C. The residue was distilled at 50 °C under reduced pressure. The fractions containing product were combined and the solvent was distilled off to obtain compound (±)-**4** (4.69 g, 57.2 mmol, 63%) as a colorless oil.

^{1}H-NMR (500 MHz, $CDCl_3$) δ [ppm] = 6.20 - 6.12 (m, 1H, H-2), 6.00 (dtq, ^3J = 6.7, 4.7 Hz, ^4J = 2.2 Hz, 1H, H-3), 3.93 (ddd, ^3J = 5.7, 3.4 Hz, ^4J = 1.8 Hz, 1H, H-1), 3.84 (tt, ^3J = 5.7, 2.7 Hz, 1H, H-5), 2.65 (^2J = 18.8 Hz, ^3J = 7.3, 2.3 Hz, 1H, H-4), 2.41 (^2J = 18.4 Hz, ^3J = 4.0, 2.0 Hz, 1H, H-4); **^{13}C-NMR** (125 MHz, $CDCl_3$) δ [ppm] = 137.5, 131.1, 58.8, 56.4, 35.3.

The data is consistent with the literature[4]

(1*S*,2*S*)- 2-aminocyclopent-3-en-1-ol ((±)-5)

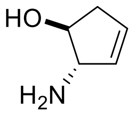

A solution of NH_3/MeOH (7 M, 40 mL) was cooled to 0 °C and cyclopentene epoxide (**4**) (500 mg, 6.09 mmol, 1.00 eq.) was added dropwise. The reaction mixture was stirred at 0 °C for 24 h and at r.t. for 64 h. Subsequently, the solvent was evaporated *in vacuo* and racemic **5** was obtained as a brown oil (316 mg, 3.19 mmol, 52%) and used in subsequent steps without further purification.

^{1}H-NMR (500 MHz, CDCl3) δ [ppm] = 5.74 (dq, 3J = 6.2, 2.1 Hz, 1H, H-3), 5.65 (dq, 3J = 6.1, 2.1 Hz, 1H, H-4), 4.04 (ddd, 3J = 7.0, 5.0, 4.2 Hz, 1H, H-1), 3.74 (dp, 3J = 5.0, 1.7 Hz, 1H, H-2), 2.74 (ddq, 2J = 16.9 Hz, 3J = 7.1, 2.0 Hz, 1H, H-5), 2.25 (ddq, 2J = 16.8 Hz, 3J = 4.9, 2.0 Hz, 1H, H-5). **^{13}C-NMR** (125 MHz, CDCl3) δ [ppm] = 133.9 (C3), 130.0 (C4), 81.5 (C1), 65.8 (C2), 40.1 (C5).

The data is consistent with the literature[5]

t-Butyl ((1*S*,5*S*)-5-hydroxycyclopent-2-en-1-yl)carbamate ((±)-6)

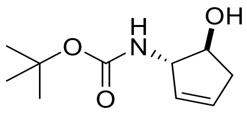

Compound (±)-**5** (3.00 g, 30.3 mmol, 1.00 eq.) was dissolved in EtOAc (300 mL) and sat. aq. $NaHCO_3$ solution (300 mL). Boc_2O (13.2 g, 60.5 mmol, 2.00 eq.) was added and the reaction mixture was stirred for 5 h at r.t. Subsequently the mixture was diluted with EtOAc (100 mL). The organic phase was washed with water (2 × 100 mL) and the aq. phase was reextracted with EtOAc (3 × 100 mL). The organic phases were combined, dried over $MgSO_4$ and the solvent was evaporated *in vacuo.* The residue was purified by flash column chromatography (i-hex/EtOAc, 1:1) to give racemic compound (±)-**6** (2.48 g, 12.5 mmol, 41%).

R_f=0.57 (i-hex/EtOAc, 1:1); **^{1}H-NMR** (500 MHz, $CDCl_3$) δ [ppm] = 5.85 (dt, ^3J = 6.6, 2.3 Hz, 1H, H-2), 5.48 (dq, ^3J = 6.4, 2.1 Hz, 1H, H-3), 4.77 (s, 1H, NH), 4.43 (tt, ^3J = 4.8, 2.1 Hz, 1H, H-1), 4.30 – 4.23 (m, 1H, H-5), 3.72 (d, ^3J = 3.0 Hz, 1H, OH), 2.82 – 2.72 (m, 1H, H-4), 2.40 – 2.31 (m, 1H, H-4), 1.46 (s, 9H, *t*Bu); **^{13}C-NMR** (125 MHz, $CDCl_3$) δ [ppm] = 133.4 (C2), 127.9 (C3), 81.3 (C5), 80.3 (C-(CH_3)$_3$), 66.2 (C1), 39.7 (C4), 28.5 (*t*Bu); **IR** (ATR): $\tilde{\nu}$ = 3328 (m), 3255 (m), 3051 (w), 2978 (w), 2943 (w), 2377 (w), 2314 (w), 1735 (w), 1667 (s), 1559 (s), 1390 (m), 1370 (m), 1362 (s), 1324 (s), 1299 (s), 1276 (s), 1250 (s), 1167 (s), 1101 (s), 1081 (s), 1051 (s), 1030 (m), 1017 (m), 994 (s), 892 (s), 856 (m), 736 (m), 713 (s), 575 (m), 452 (m), 427 (s) cm^{-1}; **EI-HRMS** m/z for [$C_{10}H_{17}NO_3$], calcd. [M-*t*Bu+H]$^+$ = 143.0582; found [M-*t*Bu+H]$^+$ = 143.0576.

t-Butyl ((1*S*,5*S*)-5-hydroxycyclopent-2-en-1-yl)carbamate ((+)-7)

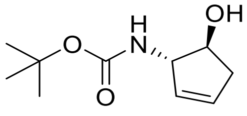

Compound (±)-**6** (1.50 g, 7.53 mmol, 1.00 eq.) was dissolved in isopropenyl acetate (9 mL). To this solution, *Candida antarctica lipase B* (150 mg, 10% by wt.) was added and stirred for 18 h at r.t. The reaction mixture was filtered, concentrated *in vacuo* and purified by flash column chromatography (i-hex/EtOAc, 4:1) to give compound (+)-**7** (680 mg, 3.41 mmol, 45%, 93% ee). The enantiopurity was further increased to >98% by a single recrystallization from hexane:DCM.

R_f=0.16 (i-hex/EtOAc, 4:1); **^{1}H-NMR** (500 MHz, $CDCl_3$) δ [ppm] = 5.82 (dq, ^3J = 4.7, 2.3 Hz, 1H, H-2), 5.51 – 5.45 (m, 1H, H-3), 4.90 – 4.85 (m, 1H, NH), 4.42 – 4.35 (m, 1H, H-1), 4.21 (q, ^3J = 5.5 Hz, 1H, H-5), 3.99 (s, 1H, OH), 2.72 (ddq, ^2J = 17.0 Hz, ^3J = 7.1, 2.2 Hz, 1H, H-4), 2.35 – 2.27 (m, 1H, H-4), 1.42 (s, 9H, *t*Bu); **^{13}C-NMR** (125 MHz, $CDCl_3$) δ [ppm] = 156.9 (CO-O*t*Bu), 133.2 (C2), 128.1 (C3), 80.5 (C5), 80.1 (C-(CH_3)$_3$), 65.9 (C1), 39.6 (C4), 28.4 (*t*Bu); **IR** (ATR) $\tilde{\nu}$ (cm^{-1}) = 3350 (m), 3067 (w), 3006 (w), 2972 (w), 2933 (w), 2851 (w), 2377 (w), 2312 (w), 1735 (m), 1674 (s), 1512 (s), 1466 (m), 1444 (m), 1390 (m), 1366 (s), 1332 (s), 1279 (s), 1267 (s), 1255 (s), 1227 (m), 1163 (s), 1089 (m), 1058 (s), 990 (s), 944 (w), 892 (s), 783 (m),

773 (m), 740 (m), 714 (s), 594 (s), 420 (m) cm^{-1}. **EI-HRMS** m/z for [$C_{10}H_{17}NO_3$]; calcd. [M-*t*Bu+H]$^+$ = 143.0582; found [M-*t*Bu+H]$^+$ = 143.0577.

The data is consistent with the literature[6]

t-butyl ((1S,5S)-5-((triisopropylsilyl)oxy)cyclopent-2-en-1-yl)carbamate (8)

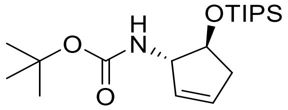

Compound (+)-**7** (116 mg, 582 µmol, 1.00 eq.) was dissolved in DCM (5 mL) under N_2 atmosphere and TEA (97.4 µl, 699 µmol, 1.20 eq.) and DMAP (7.1 mg, 58.2 µmol. 0.1 eq.) was added. The solution was cooled to 0 °C and TIPS-Cl (168 µl, 785 µmol, 1.35 eq.) was added dropwise. Subsequently the solution was warmed to r.t. and stirred for further 24 h. The reaction mixture was quenched with sat. $NaHCO_3$ solution and extracted with EtOAc (3 × 10 mL). The combined organic phases were dried over Na_2SO_4, filtered and concentrated *in vacuo*. The crude residue was purified by flash column chromatography on silica gel (i-hex/EtOAc, 10:1) to afford **8** (182 mg, 511 µmol, 88%) as a white solid.

R_f=0.57 (i-hex/EtOAc, 8:1); **^{1}H-NMR** (500 MHz, $CDCl_3$) δ [ppm] = 5.66 (dq, *J* = 6.3, 2.2 Hz, 1H), 5.51 (dq, *J* = 6.1, 2.1 Hz, 1H), 4.45 – 4.31 (m, 2H), 4.17 (dt, *J* = 7.1, 4.4 Hz, 1H), 2.58 (ddq, *J* = 16.6, 6.6, 2.0 Hz, 1H, (CH_2)), 2.18 (ddq, *J* = 16.7, 4.3, 2.1 Hz, 1H (CH_2)), 1.34 (s, 9H, *t*-Bu), 1.05 – 0.90 (m, 21H, TIPS); **^{13}C-NMR** (125 MHz, $CDCl_3$) δ [ppm] = 155.1, 130.9, 130.9, 79.9, 79.2, 77.4, 77.2, 76.9, 65.0, 41.2, 28.7, 28.5, 18.1, 18.1, 18.0, 18.0, 12.5, 12.4, 12.3, 12.2, 12.1; **IR** (ATR) $\tilde{\nu}$ (cm^{-1}) = 3326 (m), 2963 (m), 2864 (s),1701 (m), 1694 (vs), 1600 (s), 1493 (s), 1408 (vs), 1366 (m), 1345 (m), 1307 (s), 1286 (m), 1217 (w), 1141 (m), 1100 (m), 1082 (w), 1050 (m), 994 (w), 916 (s), 898 (m), 880 (m), 865 (s), 776 (s), 715 (w), 533 (vs), 498 (w), 464 (w), 421 (w); **ESI-HRMS** m/z for [$C_{19}H_{37}NO_3Si$] calcd. [M+Na]$^+$ = 378.2440; found [M+Na]$^+$ = 378.2426.

(1S,5S)-5-((triisopropylsilyl)oxy)cyclopent-2-en-1-amine (9)

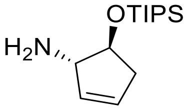

To compound **8** (144 mg, 563 µmol, 1.00 eq.) in DCM (1.5 mL) was added TFA (729 µL, 13.2 mmol, 23.5 eq.). The reaction mixture was stirred at 0 °C for 10 min and 10 at r.t. and neutralized with aq. $NaHCO_3$ solution. The mixture was extracted with DCM (3 × 10 mL), dried over $MgSO_4$ and concentrated *in vacuo*. The residue was purified by flash column chromatography (DCM/MeOH, 0 → 3%) to afford compound **9** (96 mg, 523 µmol, 93%).

R_f=0.35 (DCM/MeOH, 9:1); **^{1}H-NMR** (500 MHz, $CDCl_3$) δ [ppm] = 5.77 – 5.72 (m, 1H), 5.69 – 5.64 (m, 1H), 4.14 (dt, *J* = 7.4, 4.1 Hz, 1H), 3.80 (s, 1H), 2.54 – 2.35 (m, 2H), 2.32 – 2.23 (m, 1H), 1.07 (t, *J* = 4.2 Hz, 23H).; **^{13}C-NMR** (125 MHz, $CDCl_3$) δ [ppm] = 132.8, 130.5, 82.4, 66.3, 41.6, 18.2, 18.2, 18.1, 12.6,

12.5, 12.4, 12.1; **IR** (ATR) $\tilde{\nu}$ (cm^{-1}) = 2954 (vs), 2921 (vs), 2866 (s), 2361 (w), 1462 (m), 1378 (m), 883 (w), 715 (w); **ESI-HRMS** m/z for [$C_{14}H_{29}NOSi$] calcd. $[M+H]^+$ = 256.2092 ; found $[M+H]^+$ =256.1992.

(R)-3,3,3-trifluoro-2-methoxy-2-phenyl-N-((1S,5S)-5-((triisopropylsilyl)oxy)cyclopent-2-en-1-yl)propenamide (10)

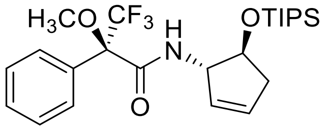

To a stirred solution of compound **9** (5.00 mg, 14.8 µmol, 1.00 eq.) and Hunig's base (3.87 µL, 22.2 µmol, 1.50 eq.) in DCM (0.2 mL), (*S*)-(+)-MTPA-Cl (3.32 µL, 17.8 µmol, 1.20 eq.) was added and the reaction mixture was stirred overnight. The mixture was concentrated *in vacuo*, the residue was taken up in aq. NH_4Cl solution (1 M, 5 mL) and extracted into DCM (3 × 5 mL). The combined organic phases were dried over $MgSO_4$ and purified by flash column chromatography (i-hex/EtOAc, 9:1) to afford compound **10** (3.00 mg, 5.42 µmol, 37%).

R_f=0.70 (i-hex/EtOAc, 9:1); **^{1}H-NMR** (500 MHz, $CDCl_3$) δ [ppm] = δ 7.56 – 7.50 (m, 2H), 7.43 – 7.34 (m, 3H), 6.63 (d, *J* = 8.8 Hz, 1H), 5.90 – 5.84 (m, 1H), 5.66 (dq, *J* = 6.2, 2.1 Hz, 1H), 4.85 (ddq, *J* = 8.8, 3.5, 1.7 Hz, 1H), 4.30 (dt, *J* = 7.0, 3.7 Hz, 1H), 3.40 (q, *J* = 1.6 Hz, 3H, OCH_3), 2.77 – 2.67 (m, 1H, CH_2), 2.37 – 2.28 (m, 1H, CH_2), 1.10 – 0.97 (m, 21H, TIPS).; **^{13}C-NMR** (200 MHz, $CDCl_3$) δ [ppm] =165.8, 132.7, 132.6, 129.6, 129.5, 128.6, 127.8, 78.7, 77.4, 77.4, 77.2, 76.9, 64.2, 55.0, 41.6, 29.9, 18.1, 12.3; **^{19}F-NMR** (377 MHz, $CDCl_3$) δ [ppm] = -68.8 (CF_3); **IR** (ATR) $\tilde{\nu}$ (cm^{-1}) = ; **ESI-HRMS** m/z for [$C_{24}H_{37}F_3NO_3Si$] calcd. $[M+H]^+$ = 473.2489; found $[M+H]^+$ = 473.2391.

4-hydroxycyclopent-2-en-1-one (15)

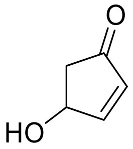

A solution of furfuryl alcohol (5.20 mL, 61.2 mmol, 1.00 eq.) and KH_2PO_4 (400 mg, 2.94 mmol, 0.05 eq.) in water (100 mL) was purged with N_2 for 2 h and then placed in a preequilibrated 125 °C heating block. The solution was stirred for 48 h before being cooled to r.t. The mixture was washed with DCM (3 × 50 mL). The organic phase was back extracted with water (50 mL), dried over $MgSO_4$, filtered and concentrated *in vacuo* to afford an amber-colored oil (2.89 g, 29.5 mmol, 48%) which was used in the subsequent reaction without further purification.

^{1}H-NMR (500 MHz, $CDCl_3$) δ [ppm] = 7.57 (dd, *J* = 5.6, 2.3 Hz, 1H), 6.24 (dd, *J* = 5.6, 1.2 Hz, 1H), 5.07 (dq, *J* = 6.0, 1.9 Hz, 1H), 2.82 – 2.76 (m, 1H), 2.32 – 2.26 (m, 1H); **^{13}C-NMR** (200 MHz, $CDCl_3$) δ [ppm] = 206.4, 162.9, 135.4, 70.5, 44.3.

The data is consistent with the literature[7]

(1R,3S)-cyclopent-4-ene-1,3-diol (16)

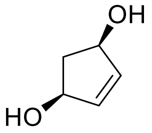

Enone **15** (750 mg, 7.65 mmol, 1.00 eq.) was dissolved in Methanol (20 mL) and $CeCl_3 \cdot 7\ H_2O$ (5.70 g, 7.65 mmol, 2.00 eq.) was added as a solution in MeOH (10 mL). The reaction was cooled to -30 °C and $NaBH_4$ (289 mg, 7.65 mmol, 1.00 eq) was added portion wise. After 1 h the reaction mixture was quenched with 5% HCl and slowly warmed to r.t. The organic phase was extracted with DCM and dried over $MgSO_4$, filtered and concentrated *in vacuo*. The residue was purified by column chromatography (DCM/MeOH, 20:1) to afford the diol **16** as a colorless oil (566 mg, 5.66 mmol, 74%).

^{1}H-NMR (500 MHz, $CDCl_3$) δ [ppm] = 5.88 (s, 2H), 4.61 (s, 2H, OH), 4.53 (dd, *J* = 7.6, 3.8 Hz, 2H), 2.63 (dt, *J* = 14.5, 7.3 Hz, 1H), 1.44 (dt, *J* = 14.4, 3.7 Hz, 1H); **^{13}C-NMR** (200 MHz, $CDCl_3$) δ [ppm] = 136.2, 74.7, 43.2; **ESI-HRMS** m/z for [$C_5H_8O_2$]; calcd. $[M+H]^+$ = 99.0441; found $[M+H]^+$ = 99.0432.

The data is consistent with the literature[7]

(1S,4R)-4-hydroxycyclopent-2-en-1-yl acetate (17)

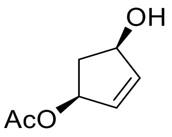

Vinyl acetate (1.33 mL, 13.98 mmol) and triethylamine (1.95 mL, 13.98 mmol) were added to a stirred solution of the diol **16** (0.200 g, 1.99 mmol) in dry THF (5 mL). Then Pancreatin (1.00 g, 5 wt equiv, Sigma 4 × USP) was added. The suspension was stirred for 24 h at r.t. and then filtered over a pad of Celite®. The solid was washed with ethyl acetate (90 mL) and the filtrate was concentrated *in vacuo*. The crude residue purified by flash column chromatography on silica gel (i-hex/EtOAc 4:1→2:1) to obtain the monoacetate **17** as a yellow solid (141 mg, 995 µmol, 50%) and the diacetate derivative as yellow oil (160 mg, 1.13 mmol, 43%).

^{1}H-NMR (500 MHz, $CDCl_3$) δ [ppm] = 6.14–6.09 (1H, m, H-2), 6.01–5.96 (1H, m, H-3), 5.54–5.45 (1H, m, H-1), 4.75–4.68 (1H, m, H-4), 2.80 (1H, dt, J = 14.7, 7.4 Hz, H-5), 2.05 (3H, s, Ac), 1.66 (1H, dt, J = 14.7, 3.8 Hz, H-5); **^{13}C-NMR** (200 MHz, $CDCl_3$) δ [ppm] = 170.9 (C=O, Ac), 138.6 (C2), 132.6 (C3), 77.2 (C1), 74.9 (C4), 40.6 (C5), 21.3 (Ac); **EI-HRMS** m/z for [$C_7H_{10}O_3$] calcd. $[M-OH]^+$ = 126.0681; found $[M-OH]^+$ = 126.0599; **Optical purity** 99.9% ee; **rotation** $[\alpha]_D^{20}$ = −70.3 ($CHCl_3$).

The data is consistent with the literature[8]

(1S,4R)-4-((triisopropylsilyl)oxy)cyclopent-2-en-1-yl acetate (18)

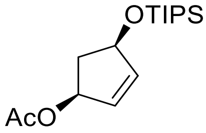

17 (90 mg, 633 µmol, 1.00 eq.) was dissolved in DCM (5 mL) and TEA (265 µL, 1.90 mmol, 3.00 eq.) and DMAP (7.73 mg, 63.3 µmol. 0.1 eq.) was added. The solution was cooled to 0 °C and TIPS-Cl (183 µL, 855 µmol, 1.35 eq.) was added dropwise. Subsequently the solution was warmed to r.t. and stirred for further 24 h. The reaction mixture was quenched with sat. $NaHCO_3$ solution and extracted with EtOAc (3 × 10 mL). The combined organic phases were dried over Na_2SO_4, filtered and concentrated *in vacuo*. The crude residue was purified by flash column chromatography on silica gel (i-hex/EtOAc, 10:1) to afford **18** (168 mg, 563 µmol, 89%) as a white solid.

R_f=0.25 (i-hex/EtOAc, 10:1); **^{1}H-NMR** (500 MHz, $CDCl_3$) δ [ppm] = 6.03 (dt, *J* = 5.6, 1.6 Hz, 1H), 5.89 (dt, *J* = 5.7, 1.6 Hz, 1H), 5.46 (ddq, *J* = 7.4, 5.2, 1.3 Hz, 1H), 4.80 (ddq, *J* = 7.2, 4.9, 1.5 Hz, 1H), 2.85 (dt, *J* = 13.7, 7.3 Hz, 1H), 2.05 (s, 3H), 1.65 (dt, *J* = 13.7, 5.1 Hz, 1H), 1.16 – 1.01 (m, 21H); **^{13}C-NMR** (200 MHz, $CDCl_3$) δ [ppm] = 171.11, 139.27, 131.20, 77.36, 77.04, 74.99, 41.66, 21.36, 18.11, 18.09, 17.90, 12.45, 12.22; **IR** (ATR) $\tilde{\nu}$ (cm^{-1}) = 2944 (s), 2892 (m), 2866 (s), 2359 (m), 2342 (m), 1738 (s) 1464 (w), 1368 (m), 1237 (s), 1129 (w), 1106 (m), 1050 (m), 1020 (m), 882 (m), 684 (w) cm^{-1}; **ESI-HRMS** m/z for [$C_5H_8O_2$]; calcd. $[M+H]^+$ = 299.2037; found $[M+H]^+$ = 299.3062.

The data is consistent with the literature[8]

(1S,4R)-4-((triisopropylsilyl)oxy)cyclopent-2-en-1-ol (19)

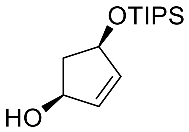

A mixture of **18** (292 mg, 978 µmol, 1.00 eq.) and K_2CO_3 (135 mg, 978 µmol, 1.00 eq.) in MeOH (10 mL) was stirred at r.t. for 90 min. The reaction mixture was diluted with $CHCl_3$ and washed with sat. aq. NH_4Cl and brine. The organic phase was dried over Na_2SO_4 and concentrated *in vacuo.* The crude residue was chromatographed on silica gel (*i*-hex/EtOAc, 4:1) to give **19** (238 mg, 978 µmol, 95%) as a white solid.

^{1}H-NMR (500 MHz, $CDCl_3$) δ [ppm] = 5.96 (s, 2H), 4.77 – 4.72 (m, 1H), 4.59 (dd, *J* = 7.0, 4.6 Hz, 1H), 2.71 (dt, *J* = 13.8, 6.9 Hz, 1H), 1.57 (dt, *J* = 13.6, 4.4 Hz, 1H), 1.26 (t, *J* = 7.1 Hz, 1H), 1.15 – 1.00 (m, 21H); **^{13}C-NMR** (200 MHz, $CDCl_3$) δ [ppm] = 137.30, 135.52, 75.25, 75.09, 45.09, 18.00, 12.08; **IR** (ATR) $\tilde{\nu}$ (cm^{-1}) = (br), 2943 (s), 2891 (m), 2866 (s), 2359 (s), 2342 (s), 1464 (m), 1365 (m), 1243 (w), 1100 (m), 1066 (m), 1033 (s), 1014 (s), 899 (s) 814 (w), 758 (w), 681 (s) cm^{-1}; **ESI-HRMS** m/z for [$C_{14}H_{28}O_2Si$] calcd. $[M+H]^+$ = 257.1931; found $[M+H]^+$ = 257.2131.

2-((1R,4R)-4-((triisopropylsilyl)oxy)cyclopent-2-en-1-yl)isoindoline-1,3-dione (20)

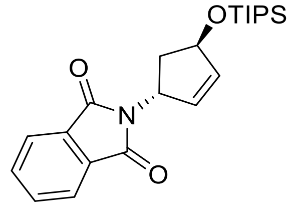

To a solution of **19** (352 mg, 1.37 mmol, 1.00 eq.) in Benzene (12 mL) were added phthalimide (404 mg, 2.75, 2.00 eq.) and triphenylphosine (720 mg, 2.75 mmol, 2.00 eq.). Then DEAD (306 µL, 975 µmol, 2.00 eq.) was added dropwise and the solution was stirred at r.t. for 24 h. The reaction was quenched by the addition of water (10 mL) and then extracted with diethylether (3 × 20 mL). The combined organic phases were washed with brine, dried over Na_2SO_4, filtered and concentrated *in vacuo*. The residue was purified by flash column chromatography (i-hex/EtOAc, 7:3) to obtain **20** as a viscous oil (418 mg, 1.08 mmol, 79%).

R_f = 0.67 (i-hex/EtOAc, 7:3); **^{1}H-NMR** (500 MHz, $CDCl_3$) δ [ppm] = 7.85 – 7.78 (m, 2H), 7.73 – 7.66 (m, 2H), 6.10 (dt, *J* = 5.6, 2.0 Hz, 1H), 5.74 (ddd, *J* = 5.6, 2.5, 1.3 Hz, 1H), 5.58 – 5.51 (m, 1H), 5.47 – 5.41 (m, 1H), 2.47 (ddd, *J* = 14.0, 7.1, 3.5 Hz, 1H), 2.20 (ddd, *J* = 13.9, 9.0, 3.8 Hz, 1H), 1.19 – 1.11 (m, 3H), 1.08 (dd, *J* = 6.7, 3.4 Hz, 18H); **^{13}C-NMR** (125 MHz, $CDCl_3$) δ [ppm] = 168.07, 139.08, 133.96, 132.04, 130.21, 123.13, 54.80, 39.25, 18.03, 18.01, 12.14; **IR** (ATR) $\tilde{\nu}$ (cm^{-1}) = 2941 (m), 2865 (m), 2361 (s), 2342 (s), 1770 (w), 1707 (s), 1465 (w), 1391 (s) 1368 (s) 1323 (w), 1118 (s), 1080 (m) 1061 (m) 1013 (w) 994 (w), 883 (s), 787 (w), 740 (w), 715 (s), 668 (s), 525 (w) cm^{-1}; **EI-MS** m/z for [$C_{22}H_{31}NO_3Si$] calcd. $[M+K+H]/2^+$ = 212.4852; found $[M+K+H]/2^+$ = 212.0705.

(1R,4R)-4-((triisopropylsilyl)oxy)cyclopent-2-en-1-amine (13)

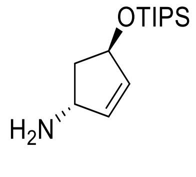

20 (1.06 g, 2.75 mmol, 1.00 eq.) was dissolved in dry EtOH (3 mL) under an Ar atmosphere. Hydrazine monohydrate (517 µL, 6.87 mmol, 2.50 eq.) was added and the reaction mixture was heated under reflux for 8 h. The reaction mixture was cooled to r.t. and Et_2O (5 mL) was added. The mixture was filtered and washed two times with EtOH (10 mL). The filtrate was concentrated *in vacuo* and chromatographed on silica gel (DCM/EtOAc, 2:1) to obtain **13** as a clear oil (618 mg, 242 mmol, 88%).

^{1}H-NMR (500 MHz, $CDCl_3$) δ [ppm] = 5.88 (ddd, *J* = 5.6, 2.1, 1.0 Hz, 2H), 5.83 (dt, *J* = 5.6, 1.8 Hz, 1H), 5.19 – 4.98 (m, 1H), 4.18 (ddd, *J* = 7.4, 3.7, 1.8 Hz, 1H), 2.13 (ddd, *J* = 13.8, 7.4, 3.3 Hz, 1H), 1.83 (ddd, *J* = 13.8, 6.9, 3.7 Hz, 1H), 1.53 (s, 2H), 1.16 – 1.00 (m, 21H); **^{13}C-NMR** (125 MHz, $CDCl_3$) δ [ppm] = 138.64,

135.33, 77.00, 56.76, 45.68, 18.13, 12.30; **IR** (ATR) $\tilde{\nu}$ (cm^{-1}) = (s), 2865 (s), 2361 8m), 1572 (m), 1462 (s), 1363 (s), 1247 (w), 1122 (m), 1056 (s), 1013 (s), 995 (m), 880 (w), 678 (s), 644 (w) cm^{-1}. **ESI-HRMS** m/z for [$C_7H_5N_5O$] calcd. $[M+H]^+$ = 256.2091; found $[M+H]^+$ = 256.2143.

(R)-3,3,3-trifluoro-2-methoxy-2-phenyl-N-((1S,5S)-5-((tri-tert-butylsilyl)oxy)cyclopent-2-en-1-yl)propenamide (21)

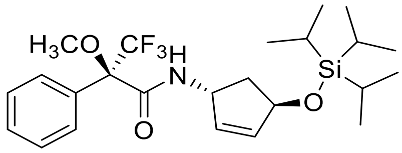

To a stirred solution of compound **13** (15.00 mg, 58.7 µmol, 1.00 eq.) and Hunig's base (15.3 µL, 88.1 µmol, 1.50 eq.) in DCM (0.3 mL), (*S*)-(+)-MTPA-Cl (13.1 µL, 170.4 µmol, 1.20 eq.) was added. The reaction mixture was stirred overnight. The mixture was concentrated *in vacuo*, the residue was taken up in aq. NH_4Cl solution (1 M, 5 mL) and extracted into DCM (3 × 5 mL). The combined organic phases were dried over $MgSO_4$ and purified by flash column chromatography (i-hex/EtOAc, 10:1) to afford compound **21** (19.0 mg, 40.0 µmol, 69%).

$\mathbf{R_f}$=0.69 (i-hex/EtOAc, 9:1); **^{1}H-NMR** (800 MHz, $CDCl_3$) δ [ppm] = 7.52 (dd, *J* = 6.7, 3.0 Hz, 2H), 7.45 – 7.37 (m, 3H), 6.61 (d, *J* = 8.3 Hz, 1H), 5.99 (dt, *J* = 5.6, 1.9 Hz, 1H), 5.88 (ddd, *J* = 5.6, 2.3, 1.1 Hz, 1H), 5.20 (tdq, *J* = 7.8, 3.8, 2.0 Hz, 1H), 5.06 (ddq, *J* = 6.8, 3.3, 1.7 Hz, 1H), 3.40 (q, *J* = 1.6 Hz, 3H), 2.23 (ddd, *J* = 14.1, 7.8, 3.5 Hz, 1H), 1.91 (ddd, *J* = 14.1, 6.9, 3.7 Hz, 1H), 1.15 – 0.99 (m, 21H); **^{13}C-NMR** (200 MHz, $CDCl_3$) δ [ppm] = 166.00 (C=O), 138.63 (C2), 133.13 (C3), 129.63 (C_{Ar}), 128.72 (C_{Ar}), 127.74 (C_{Ar}), 127.73 (C_{Ar}), 125.05 (CF_3), 122.74 (C4), 76.45 (C4), 55.1 (C1) 54.97 (OCH_3), 42.26 (C5), 18.10 (TIPS), 12.26 (TIPS); **^{19}F-NMR** (377 MHz, $CDCl_3$) δ [ppm] = 68.8 (CF_3); **ESI-HRMS** m/z for [$C_{31}H_{34}F_3NO_3Si$] calcd. $[M+H]^+$= 472.2489; found $[M+H]^+$ = 472.2418.

Barton-McCombie deoxygenation: O-((1S,2S)-2-((tert-butoxycarbonyl)amino)cyclopent-3-en-1-yl) 1H-imidazole-1-carbothioate intermediate and tert-butyl (R)-cyclopent-2-en-1-ylcarbamate (11)

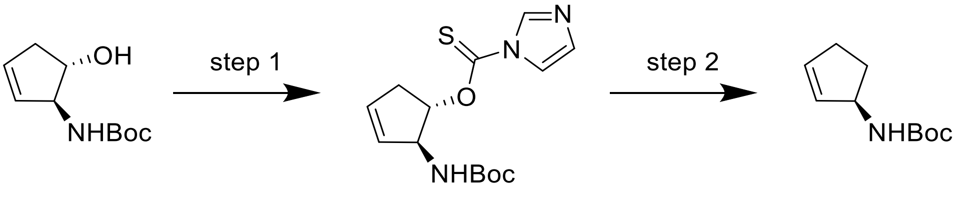

Barton McCombie deoxygenation reaction step 1:

To a solution **(+)-7** (315 mg, 1.58 mmol, 1.0 eq.) in DCM (15 mL) was added 1,1’-thiocarbonyldiimidazole (563 mg, 3.16 mmol, 2.00 eq.) was added, followed by imidazole (21.5 mg, 2316 µmol, 0.20 eq.). The reaction was stirred under an Ar atmosphere at r.t. overnight. The solution was washed with sat. aq. NaHCO3 solution (3 × 20 mL), dried over Na_2SO_4 and concentrated *in vacuo*. The residue was purified by flash column chromatography using a gradient elution (i-hex/EtOAc, 7:3)

to yield the Barton McCombie deoxygenation intermediate (401 mg, 1.58 mmol, 82%) as an off-white foam.

R_f=0.15 (i-hex/EtOAc, 7:3); **^{1}H-NMR** (800 MHz, $CDCl_3$) δ [ppm] = 8.33 (d, *J* = 1.1 Hz, 1H), 7.61 (t, *J* = 1.5 Hz, 1H), 7.04 – 6.99 (m, 1H), 5.97 – 5.90 (m, 1H), 5.78 – 5.70 (m, 1H), 5.67 (s, 1H), 4.96 – 4.92 (m, 1H), 4.73 (s, 1H), 3.15 – 3.05 (m, 1H), 2.52 – 2.44 (m, 1H), 1.42 (s, 9H); **^{13}C-NMR** (200 MHz, $CDCl_3$) δ [ppm] = 183.6, 154.9, 137.0, 132.2, 130.8, 129.4, 117.9, 88.6, 65.9, 61.8, 37.9, 28.3; **ESI-HRMS** m/z for [$C_{14}H_{19}N_3O_3S$] calcd. $[M+H]^+$ = 310.1220; found $[M+H]^+$ = 310.1315.

Barton McCombie deoxygenation reaction step 2:

Barton McCombie deoxygenation intermediate (180 mg, 582 µmol, 1.0 eq) was dissolved in dry toluene (3 mL) and nitrogen was bubbled through the solution for 20 min. Tris(trimethylsilyl)silane (198 µL, 640 µmol, 1.1 eq.) was added dropwise, followed by AIBN (23.9 mg, 145 µmol 0.25 eq.). The solution was stirred under N_2 at 90 °C for 2.5 h. The solution was cooled to r.t., concentrated *in vacuo* and purified by flash column chromatography (i-hex/EtOAc, 3:1) to yield **11** (102 mg, 582 µmol, 96%) as a white foam.

^{1}H-NMR (500 MHz, $CDCl_3$) δ [ppm] = 5.93 – 5.87 (m, 1H), 5.68 (dd, *J* = 5.6, 2.3 Hz, 1H), 4.71 (s, 1H), 4.49 (s, 1H), 2.45 – 2.38 (m, 1H), 2.35 – 2.26 (m, 1H), 1.60 – 1.53 (m, 1H), 1.44 (s, 9H); **^{13}C-NMR** (125 MHz, $CDCl_3$) δ [ppm] = 134.2, 131.6, 77.3, 77.0, 76.7, 56.7, 31.6, 31.0, 28.4; **IR** (ATR) $\tilde{\nu}$ (cm^{-1}) = 3335 (m), 2983 (m), 2362 (m), 1677 (s), 1526 (s), 1452 (w), 1391 (w), 1365 (m), 1335 (w), 1265 (w), 1247 (s), 1164 (s), 1097 (w), 1051 (s), 1000 (m), 971 (w), 855 (m), 780 (m), 719 (w) cm^{-1}; **EI-MS** m/z for [$C_3HClNNaO$] calcd. $[M+K]^+$ = 221.9631; found $[M+K]^+$ = 221.0917.

(R)-cyclopent-2-en-1-amine (12)

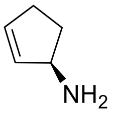

11 (85.0 mg, 463 µmol, 1.00 eq.) was dissolved in DCM (1 mL) and TFA (533 µL, 6.95 mmol, 15.0 eq.) was added. The reaction mixture was stirred at 0 °C for 10 min and 10 at r.t. and neutralised with aq. $NaHCO_3$ solution. The mixture was extracted with DCM (3 × 10 mL), dried over $MgSO_4$ and concentrated *in vacuo*. The residue was purified by flash column chromatography on silica gel (DCM/MeOH, 10:1) to obtain **12** as a yellow oil (33.5 mg, 402 µmol, 87%).

^{1}H-NMR (500 MHz, $CDCl_3$) δ [ppm] = 7.78 (s, 2H, NH2), 6.11 – 6.07 (m, 1H, C2-H), 5.71 – 5.64 (m, 1H), 4.22 – 4.16 (m, 1H), 3.61 – 3.56 (m, 1H), 2.57 – 2.31 (m, 2H, CH_2), 2.28 – 2.18 (m, 1H), 1.86 – 1.73 (m, 1H); **IR** (ATR) $\tilde{\nu}$ (cm^{-1}) = 3335 (m), 2983 (m), 2362 (m), 1677 (s), 1526 (s), 1452 (w), 1391 (w), 1365 (m), 1335 (w), 1265 (w), 1247 (s), 1164 (s), 1097 (w), 1051 (s), 1000 (m), 971 (w), 855 (m), 780 (m), 719 (w) cm^{-1}; **EI-MS** m/z for [$C_5H_{10}NClO$] calcd. $[M+K]^+$ = 122.0367; found $[M+K]^+$ = 122.4917.

4.2 Synthesis of deoxyQ (1, 2 and 3)

t-Butyl 2-(bis(*t*-butoxycarbonyl)amino)-4-(*t*-butoxy)-5-formyl-7H-pyrrolo[2,3-d]pyrimidine-7-carboxylate (22)

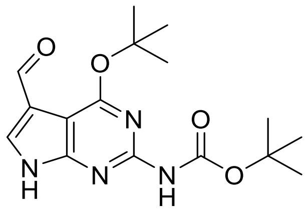

Compound **30** (500 mg, 941 µmol, 1.00 eq.) was dissolved in DCM (5 mL) and cooled to −78 °C. DIBAL-H (1 M in DCM, 2.82 mL, 2.82 mmol, 3.00 eq.) was added dropwise and the reaction was stirred for 3 h. After addition of EtOAc (2 mL) the reaction mixture was allowed to come to r.t. Half-saturated potassium sodium tartrate solution (2 mL) was added and the biphasic mixture was stirred for 30 min until phase separation was achieved. The aq. layer was separated and subsequently extracted with EtOAc (3 × 5 mL). The combined organic phases were washed with brine, dried over $MgSO_4$ and evaporated *in vacuo*. The residue was purified by flash column chromatography (i-hex/EtOAc, 10→25%) to afford compound **22** (427 mg, 799 µmol, 85%).

R$_f$=0.76 (i-hex/EtOAc, 30%); **^{1}H-NMR** (500 MHz, $CDCl_3$) δ [ppm] = 10.21 (s, 1H, CHO), 7.86 (s, 1H, C8), 1.73 (s, 9H, *t*-Bu), 1.58 (s, 9H, *t*-Bu); **^{13}C-NMR** (125 MHz, $CDCl_3$) δ [ppm] = 186.5, 152.0, 117.2, 101.4, 83.4, 77.4, 77.4, 77.2, 76.9, 60.6, 28.8, 28.5, 28.1, 14.4; **IR** (ATR) $\tilde{\nu}$ (cm^{-1}) = 3184 (b), 2971 (w), 2831 (w), 1848 (w), 1740 (s), 1664 (s), 1610 (m), 1585 (m), 1518 (m), 1481 (m), 1425 (m), 1382 (m), 1368 (s), 1278 (w), 1225 (s), 1148 (vs), 1095 (vs), 1059 (m), 899 (m), 846 (w), 586 (m), 464 (w); **ESI-HRMS** m/z for [$C_{26}H_{38}N_4O_8$] calcd. $[M+H]^+$ = 335.1714; found $[M+H]^+$ = 335.1891.

General procedure for the synthesis of deoxy-queuine via reductive amination and subsequent deprotection of the molecule (23, 24, 25)

Compound **9/13/12** (1.00 eq.) and compound **22** (65.0 mg, 135 µmol, 1.00 eq.) were dissolved in a solvent mixture (EtOAc/DCM, 1:3, 1 mL) and stirred at r.t. for 5 h. Subsequently, $NaBH(OAc)_3$ (36.4 mg, 135 µmol, 1.00 eq.) was added and stirred the mixture for 1 h. After addition of sat. aq. $NaHCO_3$ solution (1 mL) the mixture was stirred for another 15 min and then extracted with EtOAc (3 × 1 mL). The combined organic phases were dried over Na_2SO_4 and the solvent was evaporated *in vacuo*. The residue was purified by flash column chromatography (DCM/MeOH, 3 → 10%) to afford compound **23/24/25**. The obtained intermediate compounds were verified by mass spectroscopy and crude NMR and subsequently redissolved for deprotection and purification. Analytics for **23** & **24** listed below.

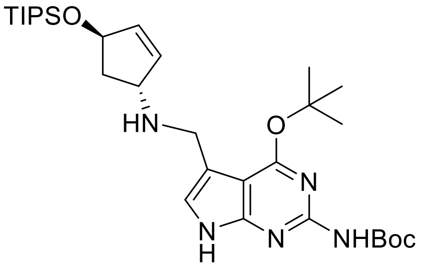

23: R_f=0.13 (DCM/MeOH, 100:5); **^{1}H-NMR** (500 MHz, $CDCl_3$) δ [ppm] = 9.88 (s, 1H, NH), 7.16 (s, 1H, C8), 6.92 (s, 1H), 5.95 (s, 1H, C5'-H), 5.13 (s, 1H, C4'-H), 3.89 (d, *J* = 10.3 Hz, 2H,), 1.67 (s, 10H), 1.55 (s, 9H), 1.14 – 1.00 (m, 22H); **^{13}C-NMR** (125 MHz, $CDCl_3$) δ [ppm] = 162.8, 153.2, 151.2, 150.7, 136.9, 101.6, 82.3, 81.3, 77.4, 77.2, 76.9, 76.8, 62.7, 40.7, 29.8, 28.9, 28.8, 28.5, 28.4, 18.1, 18.1, 18.1, 12.5, 12.2, 12.0; **IR** (ATR) $\tilde{\nu}$ (cm^{-1}) = **ESI-HRMS** m/z for [$C_{30}H_{51}N_5O_4Si$] calcd. $[M+H]^+$ = 574.3783; found $[M+H]^+$ = 574.3772.

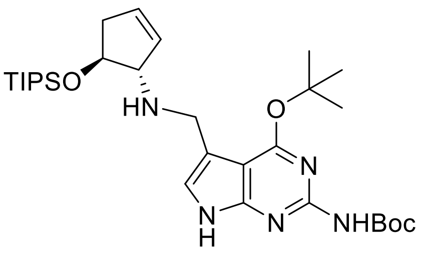

24: R_f=0.13 (DCM/MeOH, 100:5); **^{1}H-NMR** (500 MHz, $CDCl_3$) δ [ppm] = 8.94 (s, 1H, NH), 7.10 (s, 1H, NH), 6.84 (d, *J* = 2.1 Hz, 1H, C8), 5.78 (dq, *J* = 6.1, 2.0 Hz, 1H, C5'-H), 5.67 (dq, *J* = 6.2, 2.1 Hz, 1H, (C4'-H)), 4.31 (dt, *J* = 6.3, 2.9 Hz, 1H,C2'-H), 3.92 (d, *J* = 1.7 Hz, 2H, CH_2), 3.67 (d, *J* = 2.9 Hz, 1H, C1'-H), 2.76 (ddq, *J* = 16.8, 6.3, 2.1 Hz, 1H, CH_2''). 2.29 – 2.19 (m, 1H, CH_2'), 1.67 (s, 9H), 1.54 (s, 9H), 1.05 – 1.02 (m, 21H); **^{13}C-NMR** (125 MHz, $CDCl_3$) δ [ppm] = 163.3, 154.1, 151.6, 150.9, 130.9, 119.1, 101.8, 82.4, 81.1, 79.0, 77.4, 77.4, 77.2, 76.9, 71.8, 44.1, 42.0, 29.9, 28.9, 28.5, 18.2, 18.2, 12.5; **IR** (ATR) $\tilde{\nu}$ (cm^{-1}) = **ESI-HRMS** m/z for [$C_{30}H_{51}N_5O_4Si$] calcd. $[M+H]^+$ = 574.3783; found $[M+H]^+$ = 574.3772.

2-amino-5-((((1R,4R)-4-hydroxycyclopent-2-en-1-yl)amino)methyl)-3,7-dihydro-4H-pyrrolo[2,3-d]pyrimidin-4-one (1)

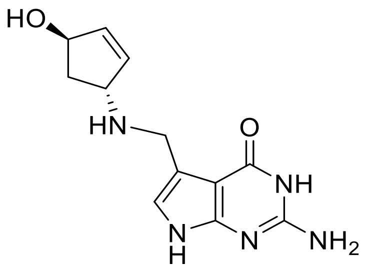

Compound **23** (35.0 mg, 43.6 µmol, 1.00 eq.) was dissolved in THF (1 mL) and cooled to 0 °C. Acetic acid (5.00 µL, 87.3 µmol, 2.00 eq.) and TBAF (1.0 M in THF, 87.3 µL, 87.3 µmol, 2.00 eq.) were added and the reaction mixture was allowed to come to r.t. and stirred for 120 h. The solvent was evaporated *in vacuo*. The residue was dissolved in DCM (4 mL) and TFA (0.1 mL, 1.31mmol, 29.0 eq.) was added. The formed suspension was evaporated to dryness and redissolved in THF (1 mL) and aq. HCl solution (6 M, 1 mL) was added. The reaction mixture was stirred for 5 min, diluted with water and neutralized with aq. NH_4OH solution. The solution was evaporated *in vacuo* and the crude product was purified by HPLC (H_2O/MeCN, 0 → 15%, 45 min) to afford compound **1** (9.00 mg, 34.0 µmol, 79%).

^{1}H-NMR (800 MHz, D_2O) δ [ppm] = 6.87 (s, 1H), 6.27 (dt, *J* = 5.8, 1.8 Hz, 1H), 6.08 (ddd, *J* = 5.8, 2.2, 1.3 Hz, 1H), 5.05 (ddd, *J* = 7.2, 3.6, 1.7 Hz, 1H), 4.54 (ddd, *J* = 7.9, 3.7, 1.8 Hz, 1H), 4.23 (d, *J* = 3.2 Hz, 2H), 2.39 (ddd, *J* = 15.2, 7.1, 3.7 Hz, 1H), 2.15 (ddd, *J* = 15.2, 7.8, 3.4 Hz, 1H); **^{13}C-NMR** (200 MHz, D_2O) δ [ppm] = 162.8, 161.3, 152.8, 151.5, 141.2, 129.1, 119.1, 117.4, 115.1, 107.8, 98.3, 74.7, 62.2, 41.3, 36.1; **IR** (ATR) $\tilde{\nu}$ (cm^{-1}) = 3336 (br), 3138 (br), 2877 (br), 1671 (vs), 1638 (s), 1547 (s), 1523 (s), 1312 (w), 1253 (m), 1194 (w), 1128 (s), 1059 (s), 936 (w), 797 (m), 777 (s), 748 (m), 685 (s), 599 (m), 436 (w); **ESI-HRMS** m/z for [$C_{12}H_{15}N_5O_2$] calcd. $[M+H]^+$ = 262.1304; found $[M+H]^+$ = 262.1247.

2-amino-5-((((1*S*,5*S*)-5-hydroxycyclopent-2-en-1-yl)amino)methyl)-3,7-dihydro-4H-pyrrolo[2,3-d]pyrimidin-4-one (2)

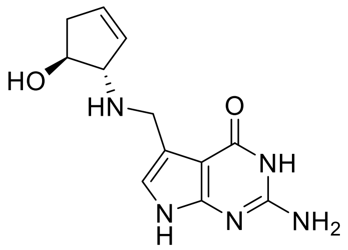

Compound **24** (35.0 mg, 43.6 µmol, 1.00 eq.) was dissolved in THF (1 mL) and cooled to 0 °C. Acetic acid (5.00 µL, 87.3 µmol, 2.00 eq.) and TBAF (1.0 M in THF, 87.3 µL, 87.3 µmol, 2.00 eq.) were added and the reaction mixture was allowed to come to r.t. and stirred for 120 h. The solvent was evaporated *in vacuo*. The residue was dissolved in DCM (4 mL) and TFA (0.1 mL, 1.31 mmol, 29.0 eq.) was added.

The formed suspension was evaporated to dryness and redissolved in THF (1 mL) and aq. HCl solution (6 M, 1 mL) was added. The reaction mixture was stirred for 5 min, diluted with water and neutralized with aq. NH_4OH solution. The solution was evaporated *in vacuo* and the crude product was purified by HPLC (H_2O/MeCN, 0 → 15%, 45 min) to afford compound **2** (7.83 mg, 31.4 µmol, 73%)

^{1}H-NMR (800 MHz, D_2O) δ [ppm] = 6.83 – 6.80 (m, 1H, H-8), 6.06 – 6.03 (m, 1H, H-2''), 5.77 (dd, ^3J = 5.9, 2.6 Hz, 1H, H-1''), 4.44 – 4.41 (m, 1H, H-4''), 4.08 (d, ^2J = 13.6 Hz, 1H, $NHCH_2$), 4.02 (d, ^2J = 13.2 Hz, 1H, $NHCH_2$), 3.85 (s, 1H, H-3''), 2.89 – 2.84 (m, 1H, H-5''), 2.31 – 2.26 (m, 1H, H-5''); **^{13}C-NMR** (200 MHz, D_2O) δ [ppm] = 162.9 (C6), 154.1 (C2), 151.7 (C4), 135.0 (C2''), 126.7 (C1''), 117.5 (C8), 112.3 (C5), 98.8 (C7), 74.4 (C4''), 70.1 (C3''), 41.9 (C7-CH_2), 40.2 (C5''); **IR** (ATR) $\tilde{\nu}$ (cm^{-1}) = 3330 (m), 3088 (m), 2927 (m), 2800 (w), 2361 (w), 1656 (s), 1618 (s), 1546 (m), 1439 (m), 1354 (m), 1308 (m), 1259 (w), 1163 (w), 1085 (m), 1016 (m), 944 (w), 839 (m), 777 (s), 737 (m), 720 (m), 688 (m), 611 (s), 568 (s), 532 (s), 463 (s), 412 (s), 402 (s) cm^{-1}; **ESI-HRMS** m/z for [$C_{12}H_{15}N_5O_2$] calcd. $[M+H]^+$ = 262.1304; found $[M+H]^+$ = 262.1297.

(R)-2-amino-5-((cyclopent-2-en-1-ylamino)methyl)-3,7-dihydro-4H-pyrrolo[2,3-d]pyrimidin-4-one (3)

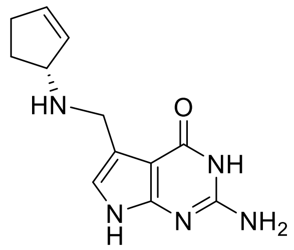

Compound **25** (67.0 mg, 170 µmol, 1.00 eq.) was dissolved in dry DCM (1 mL) and TFA (300 uL, 3.91 mmol, 23.0 eq.) was added at 0 °C. The solution was warmed to r.t. and stirred for further 30 min. The solvent was removed *in vacuo* and the residue was purified by HPLC (H_2O/MeCN, 0 → 15%, 45 min) to afford compound **3** (36.0 mg, 140 µmol, 82%).

^{1}H-NMR (800 MHz, D_2O) δ [ppm] = 6.74 (s, 1H), 6.17 (dq, *J* = 5.9, 2.2 Hz, 1H), 5.70 (dq, *J* = 4.9, 2.3 Hz, 1H), 4.27 – 4.21 (m, 1H), 4.08 (d, *J* = 5.6 Hz, 1H), 2.42 (dddq, *J* = 16.5, 8.1, 5.2, 2.5 Hz, 1H), 2.36 – 2.28 (m, 1H), 2.20 (dtd, *J* = 14.5, 8.7, 5.5 Hz, 1H), 1.86 (ddt, *J* = 14.4, 8.2, 3.8 Hz, 1H); **^{13}C-NMR** (200 MHz, D_2O) δ [ppm] = 140.93, 124.58, 119.10, 108.21, 98.46, 63.51, 40.88, 30.90, 26.34; **IR** (ATR) $\tilde{\nu}$ (cm^{-1}) = 3118 (br), 2361 (s), 2343 (s), 1677 (vs), 1654 (vs), 1559 (w), 1541 (w), 1207 (vs), 1182 (s), 1129 (s), 834 8m), 800 (m), 777 (w), 722 (w) 405 (w); **ESI-HRMS** m/z for [$C_{12}H_{15}N_5O_2$] calcd. $[M+H]^+$ = 246.1394; found $[M+H]^+$ = 246.1317.

5. NMR-spectra of important compounds

^{1}H-NMR ((±)-**5**)

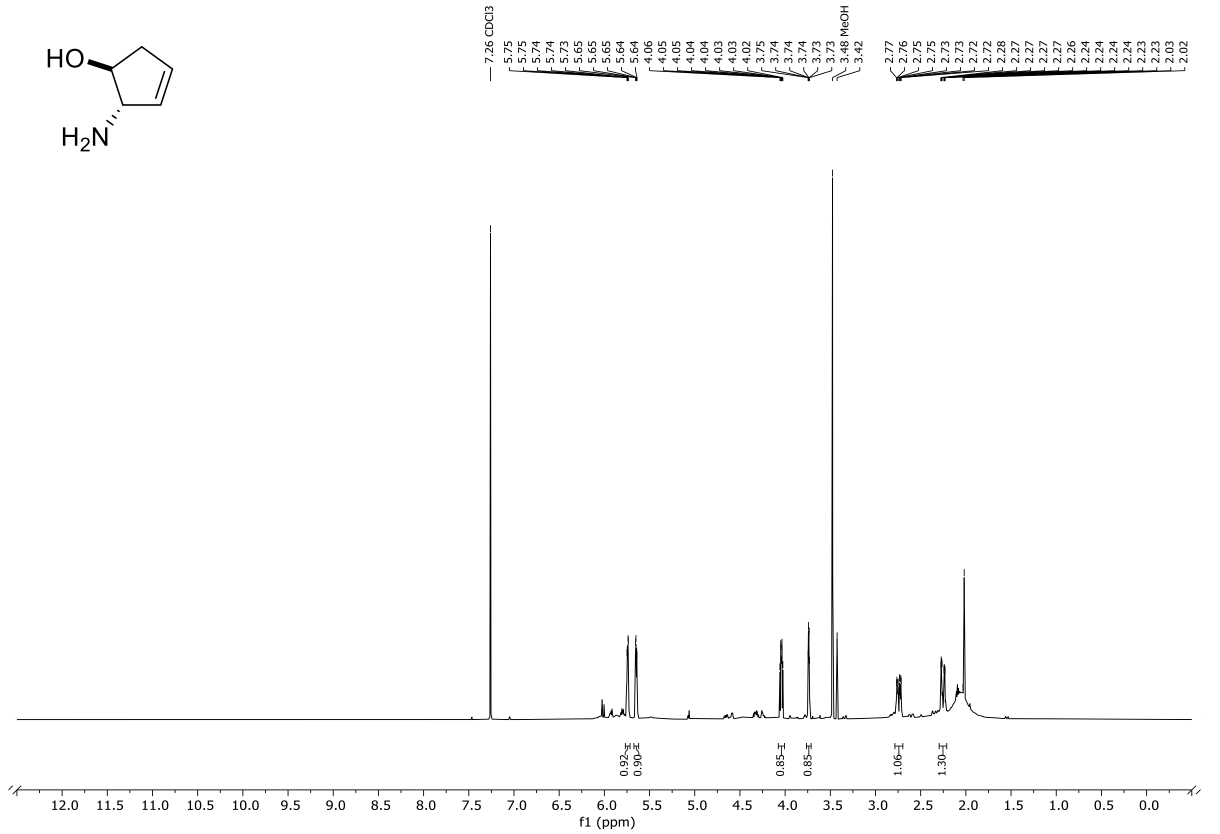

^{13}C-NMR ((±)-**5**)

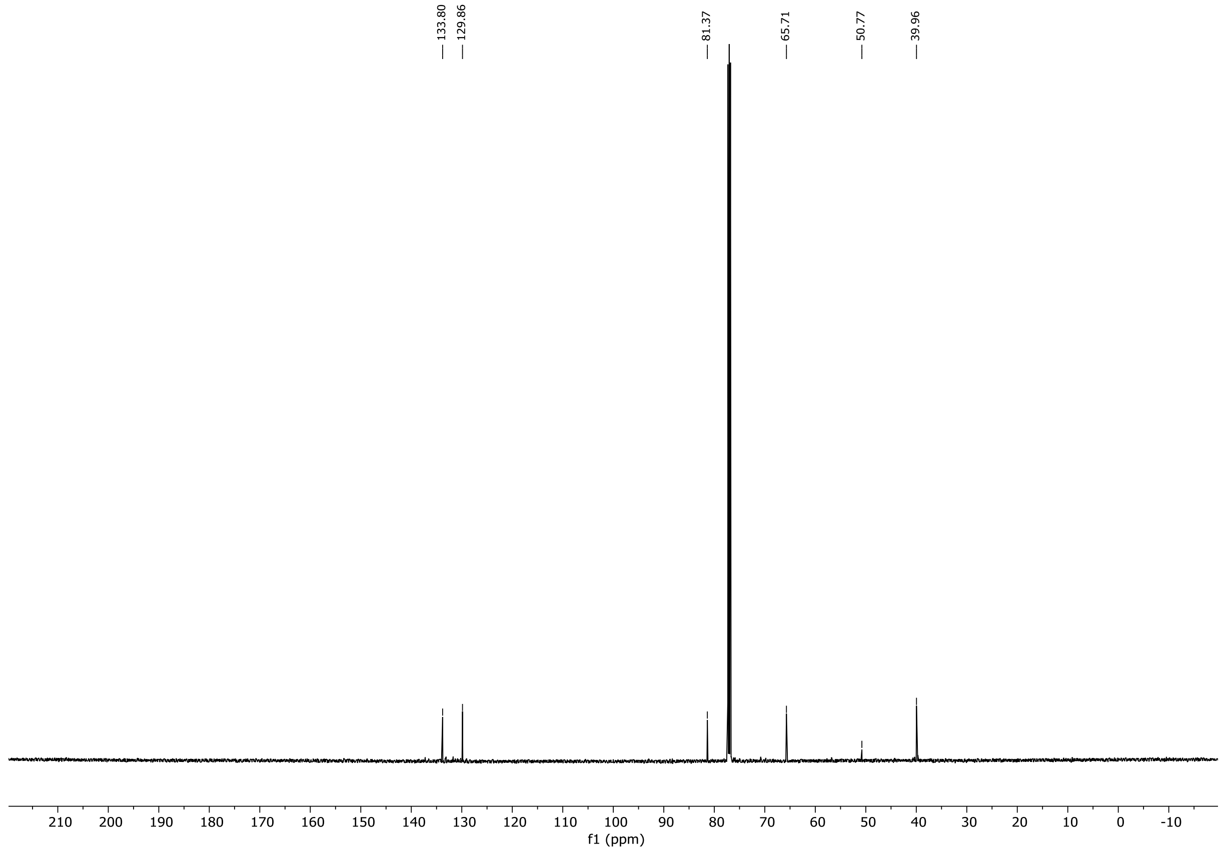

^{1}H-NMR ((+)-**7**)

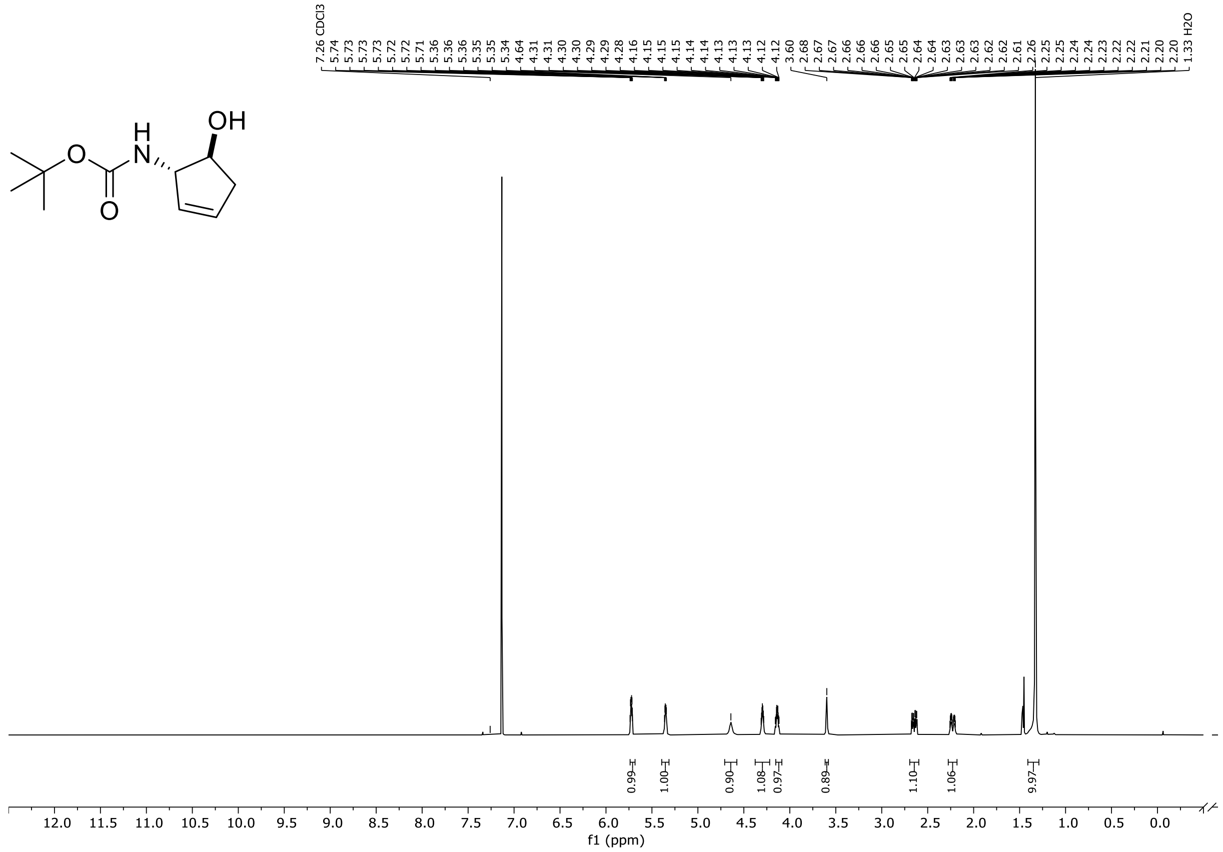

^{13}C-NMR ((+)-**7**)

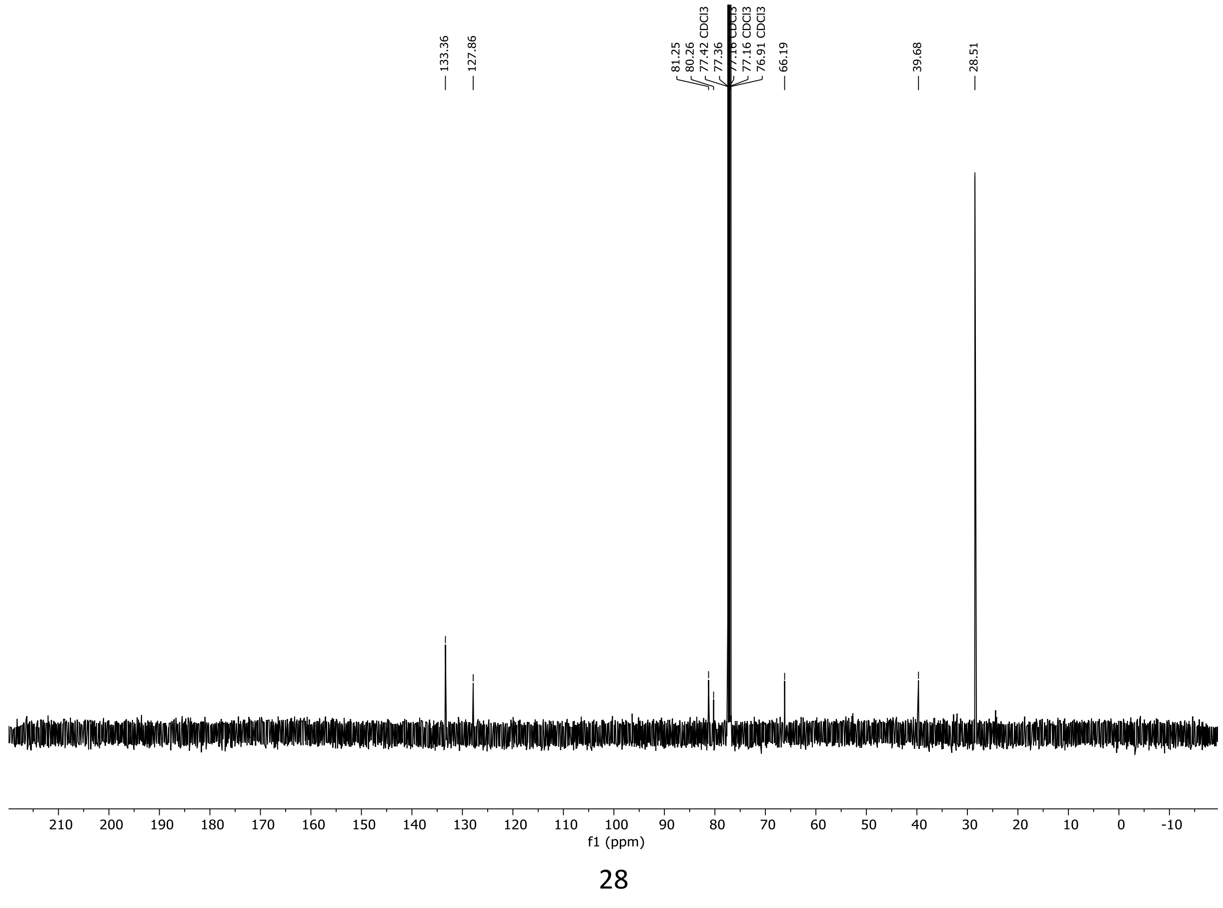

^{1}H-NMR **(8)**

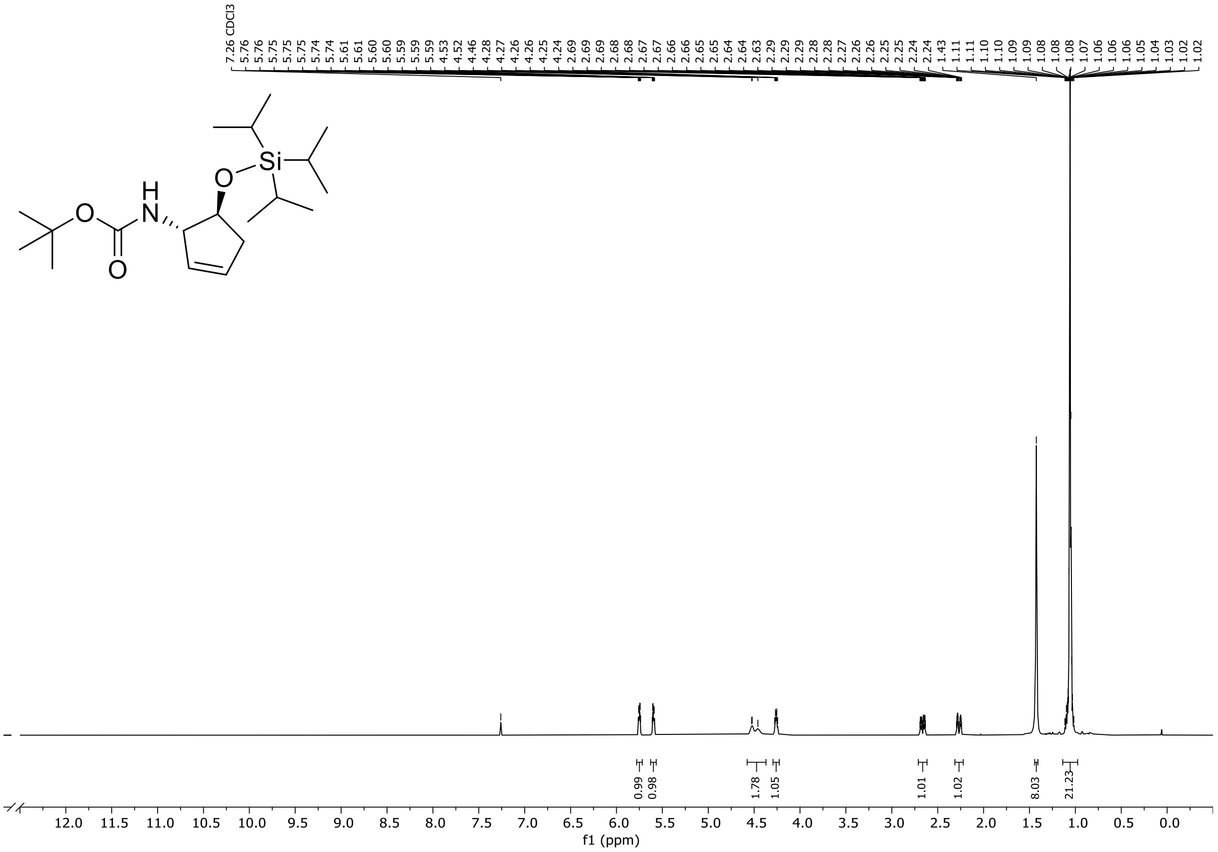

^{13}C-NMR (**8**)

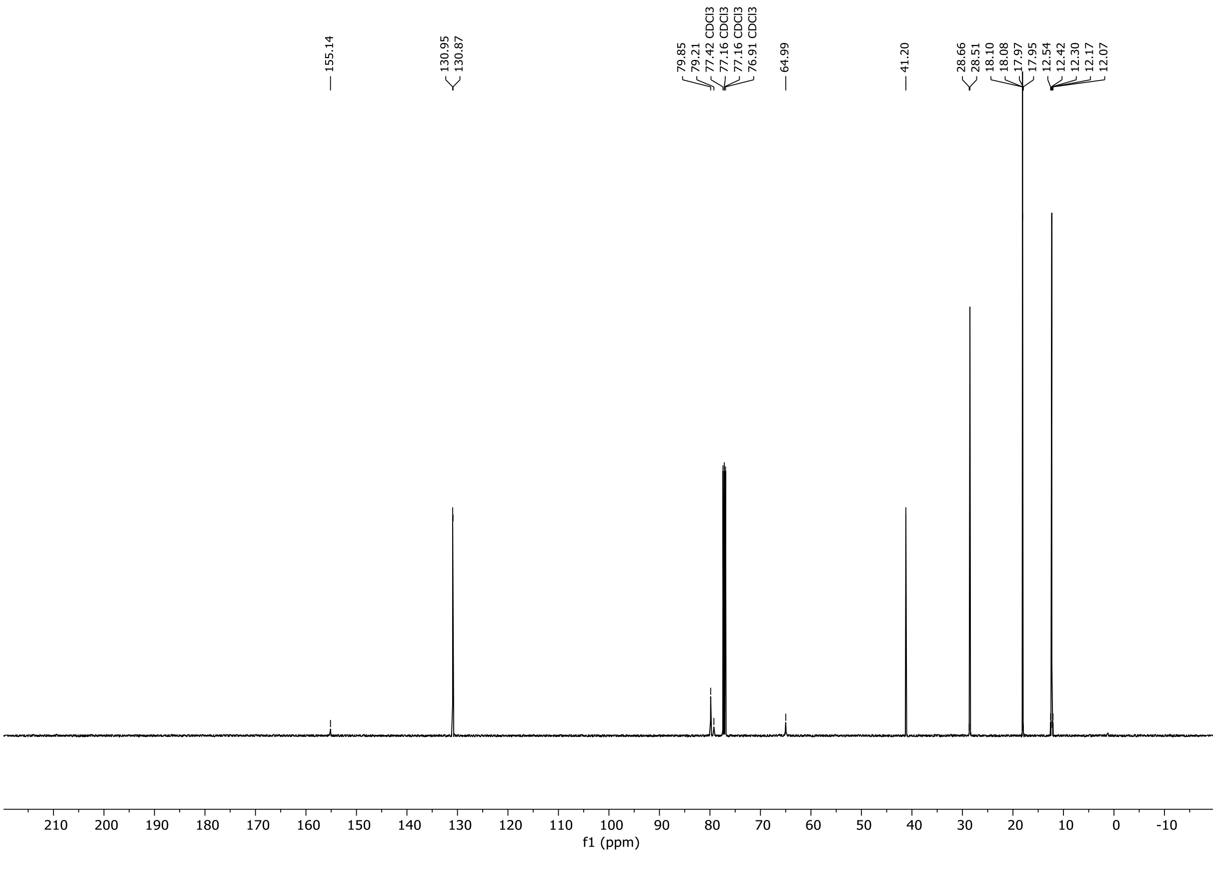

^{1}H-NMR (**9**)

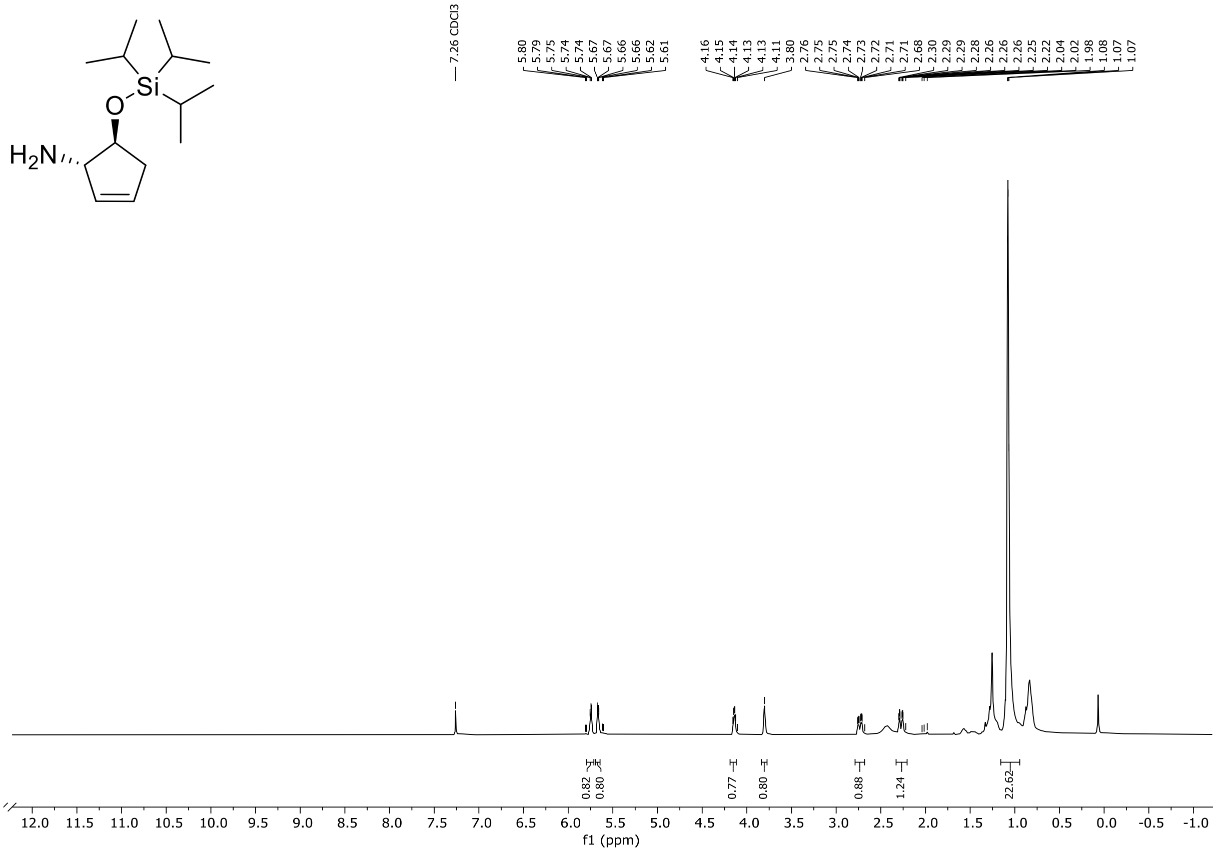

^{13}C-NMR (**9**)

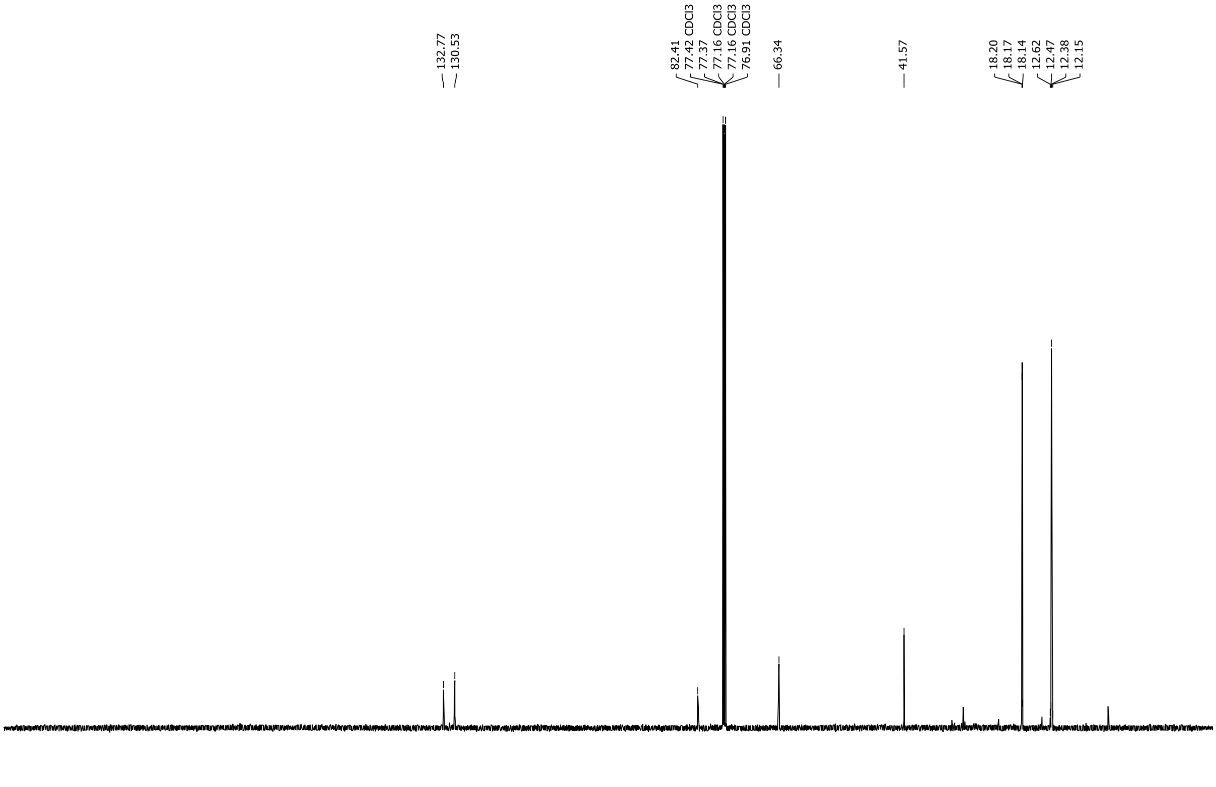

^{1}H-NMR (**10**)

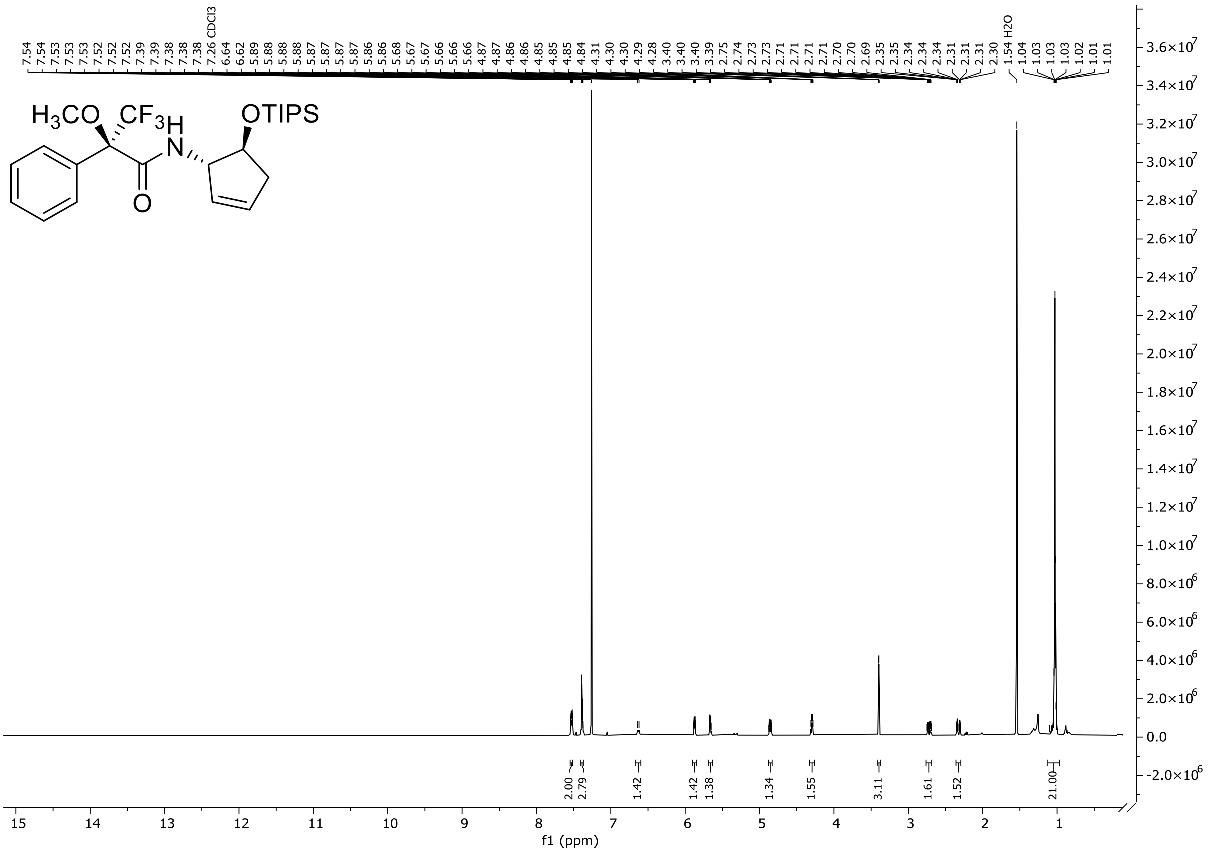

^{13}C-NMR (**10**)

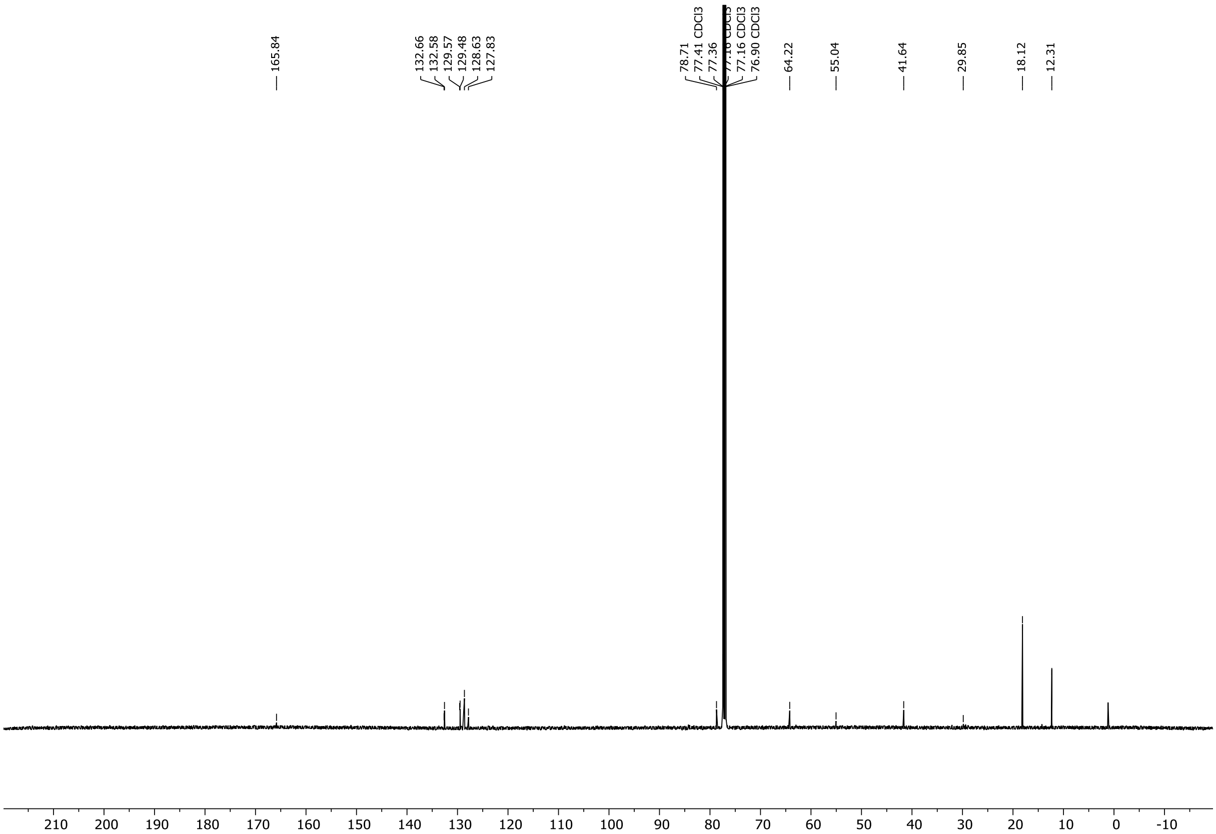

^{19}F-NMR (**10**)

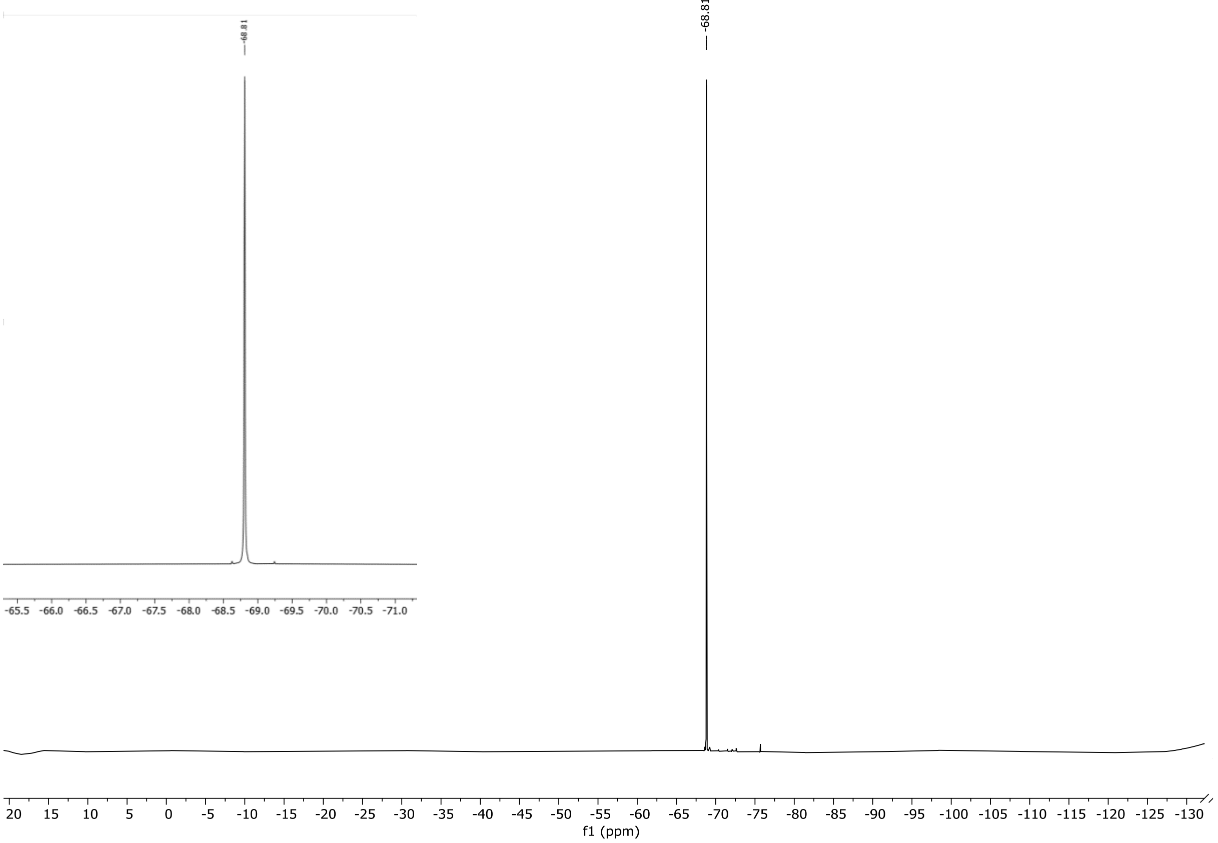

^{1}H-NMR (**15**)

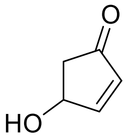

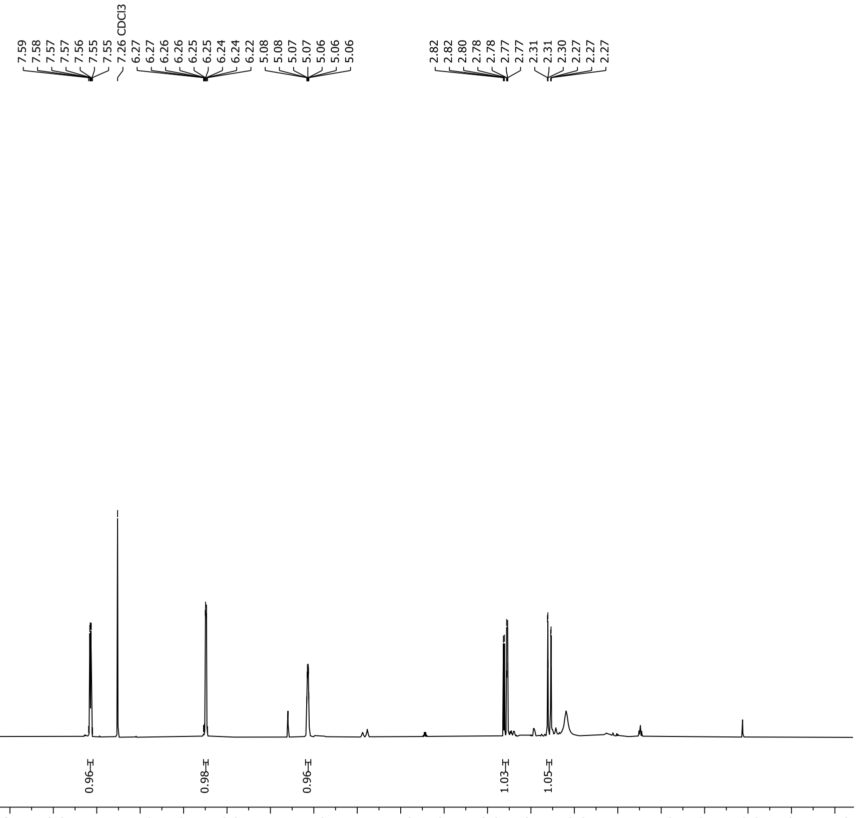

^{13}C-NMR (**15**)

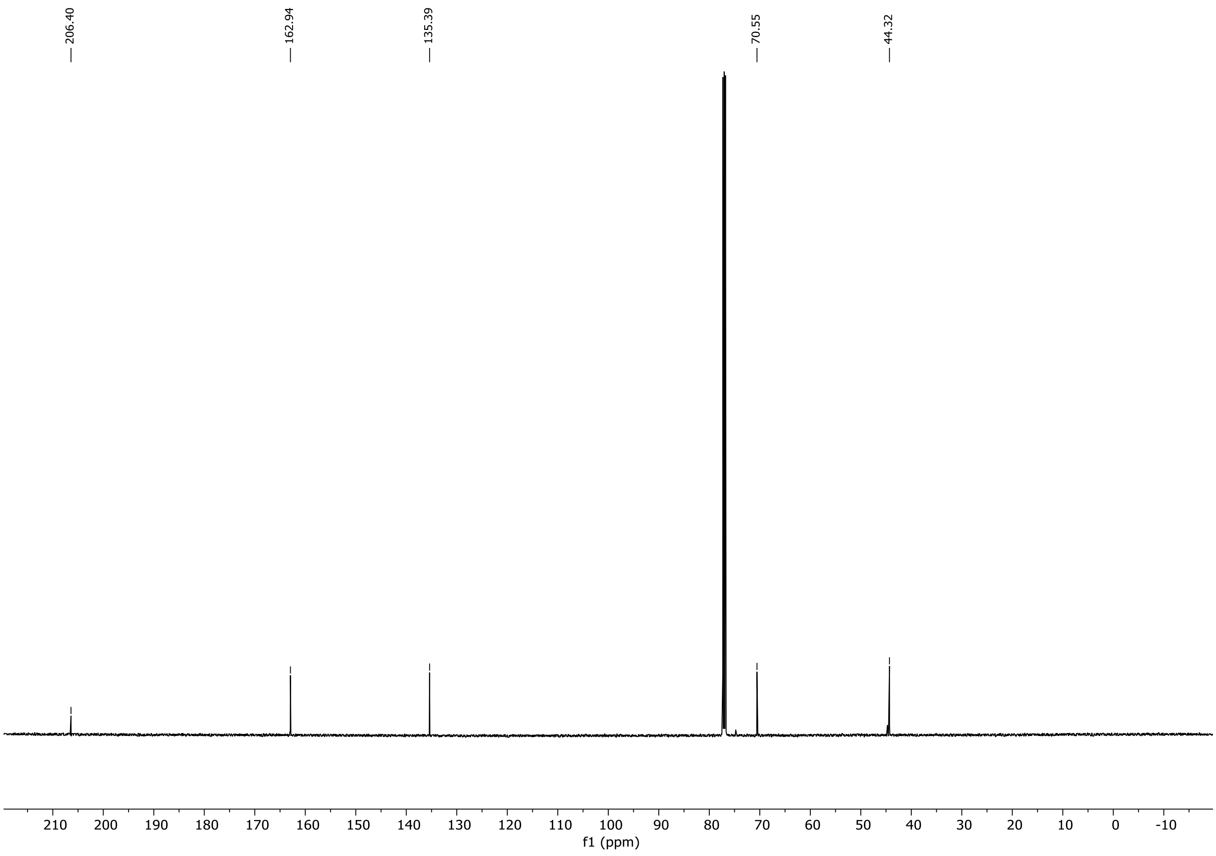

^{1}H-NMR (**16**)

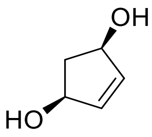

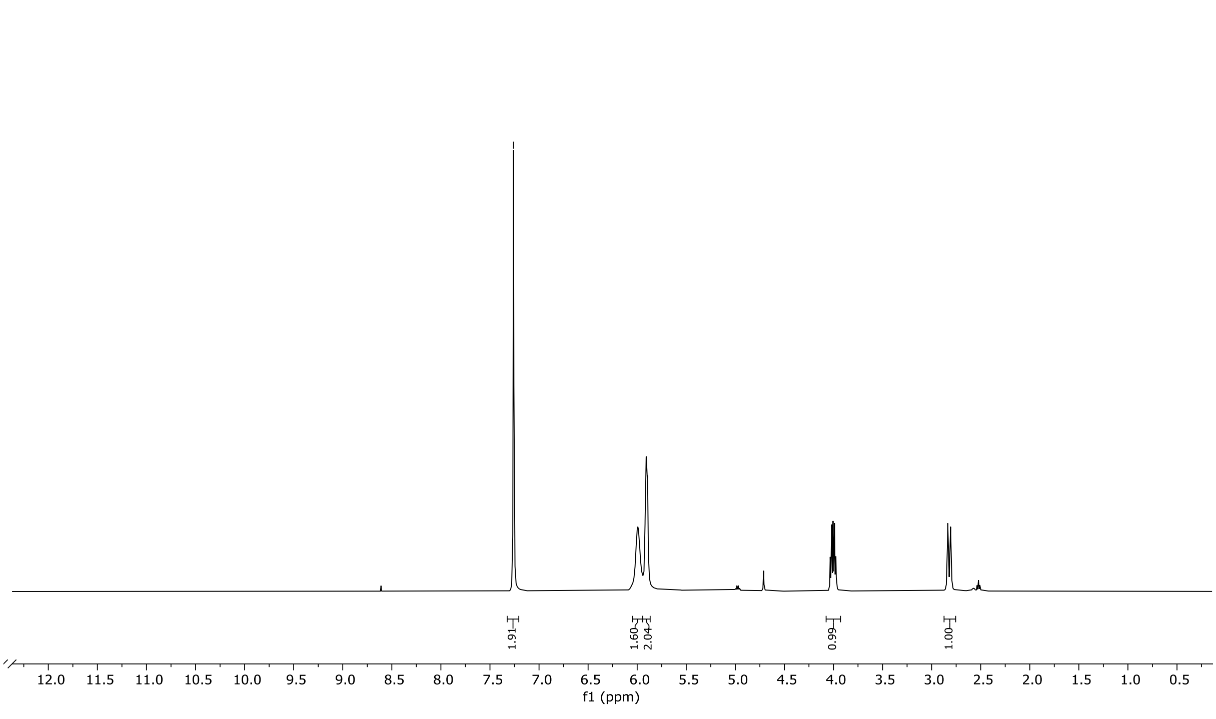

^{13}C-NMR (**16**)

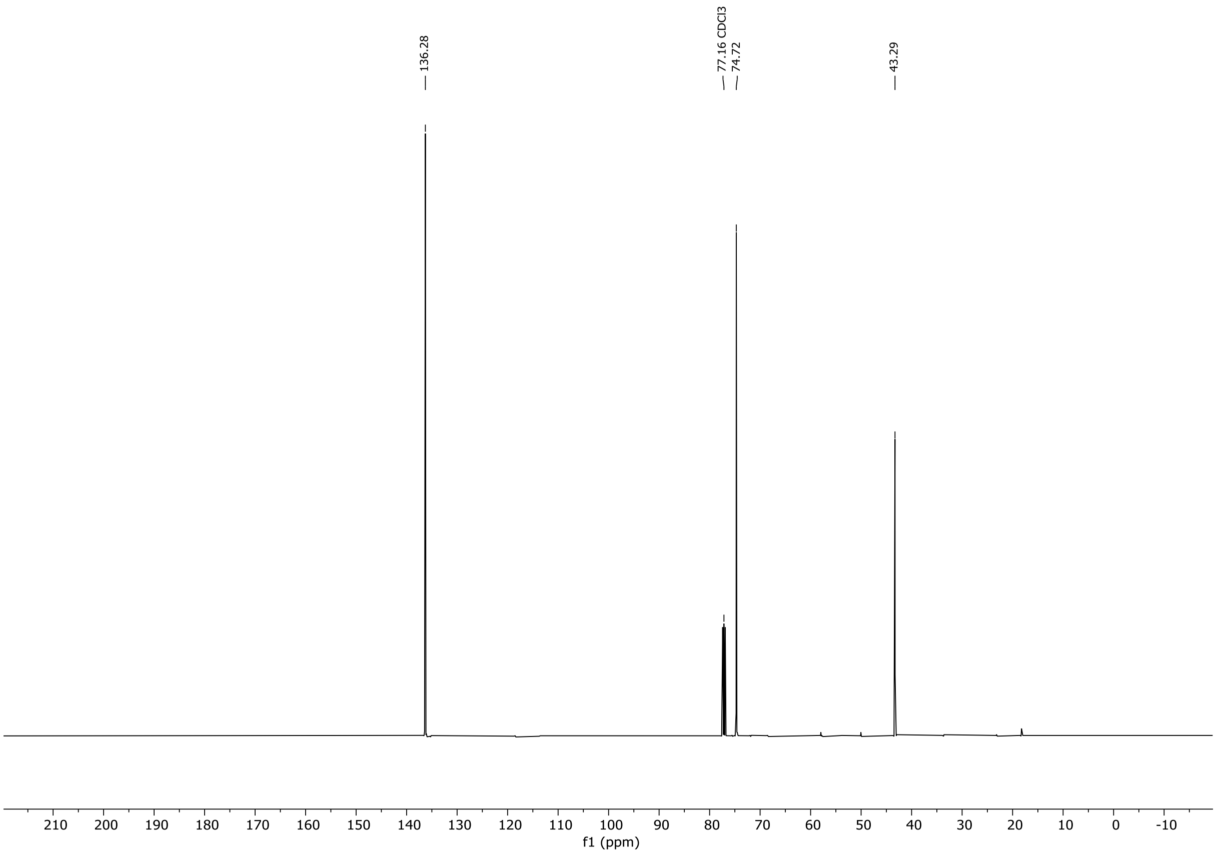

^{1}H-NMR (**17**)

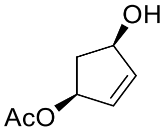

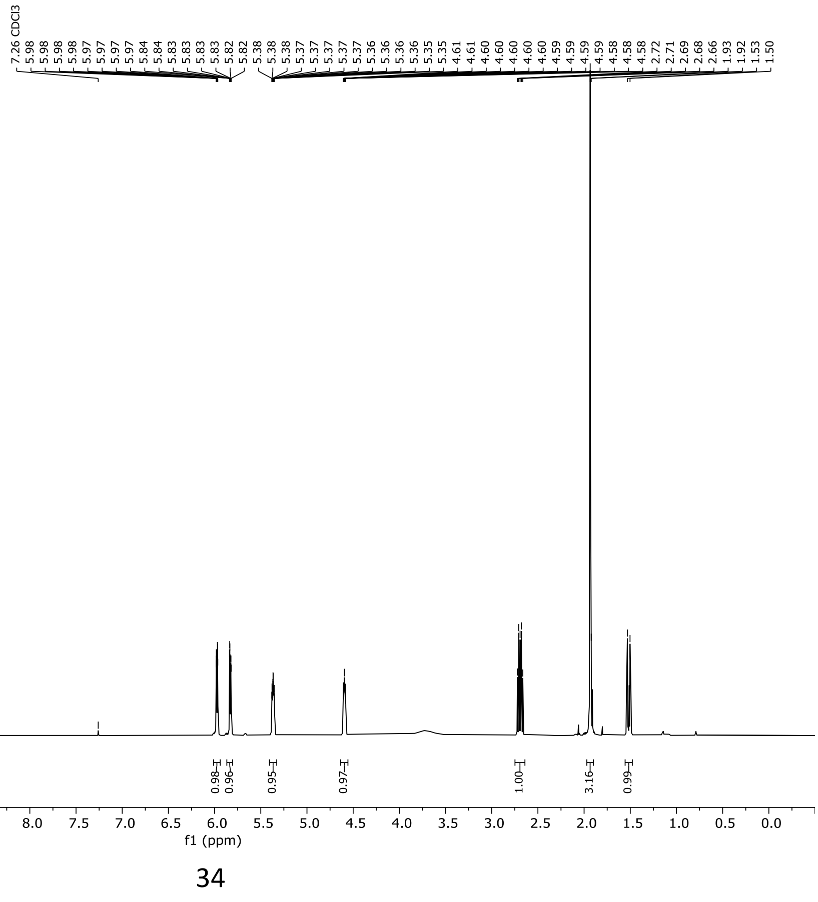

^{13}C-NMR (**17**)

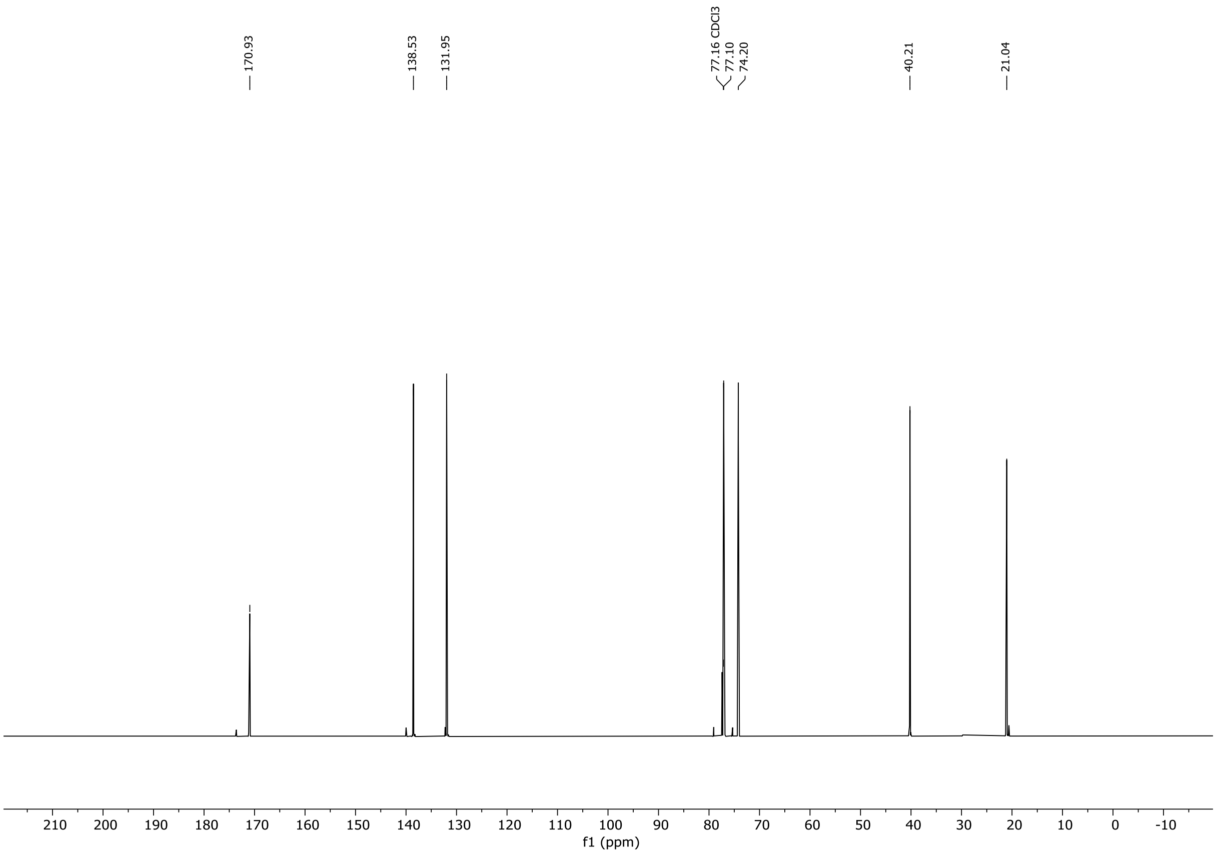

^{1}H-NMR (**18**)

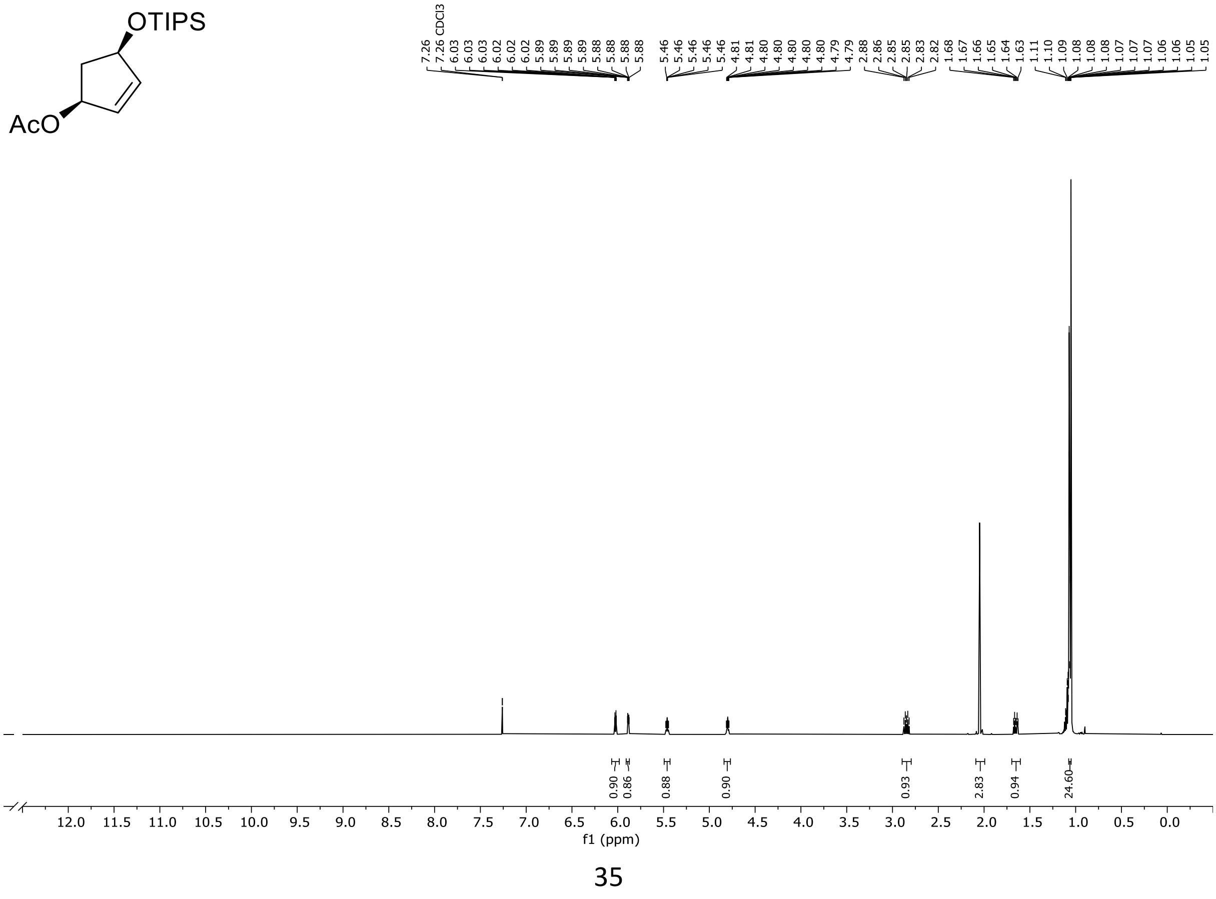

^{13}C-NMR (**18**)

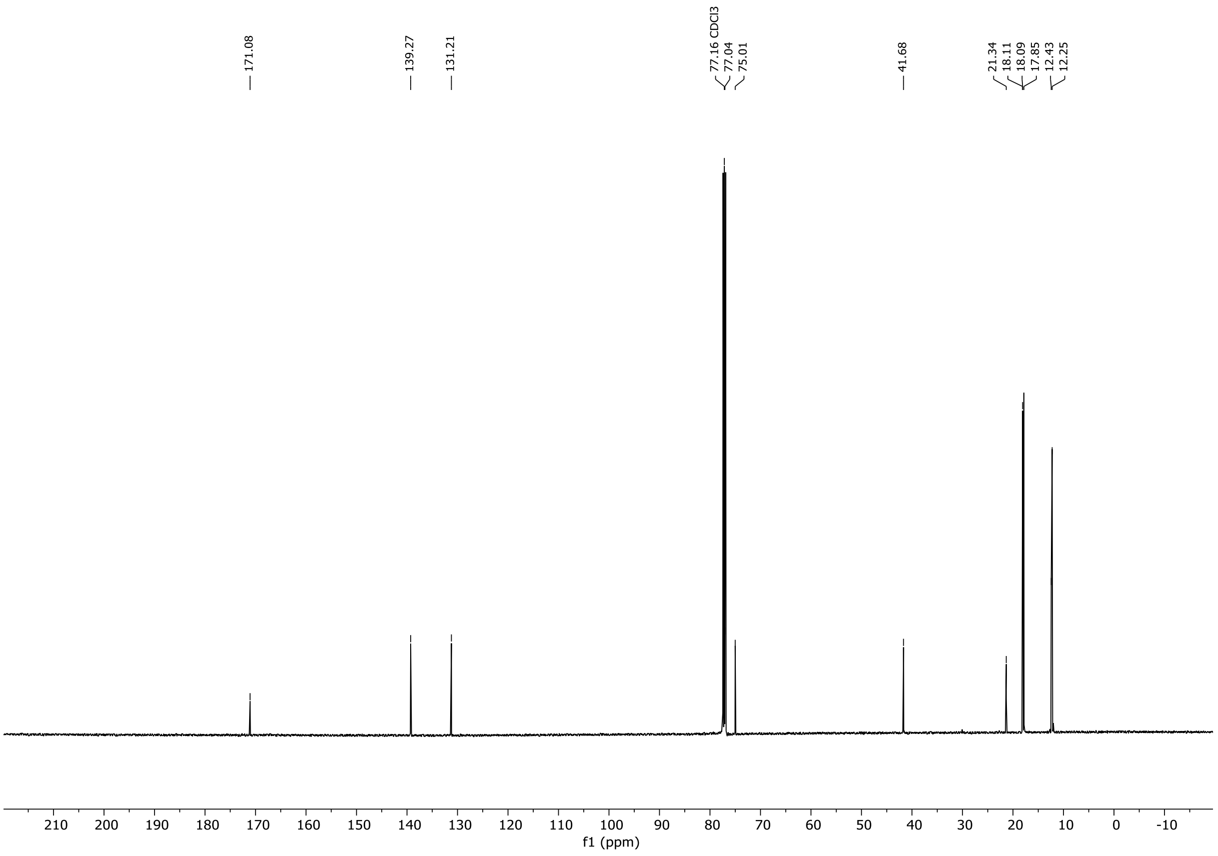

^{1}H-NMR (**19**)

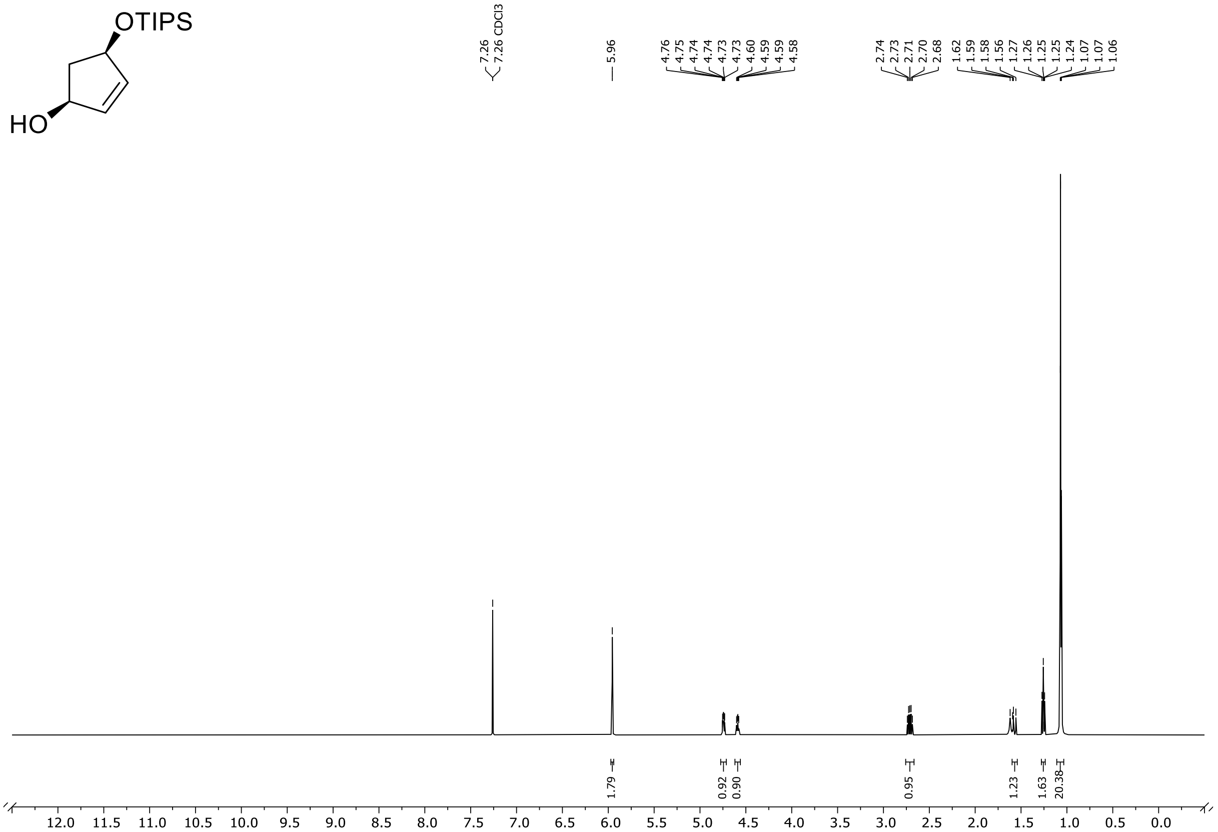

^{13}C-NMR (**19**)

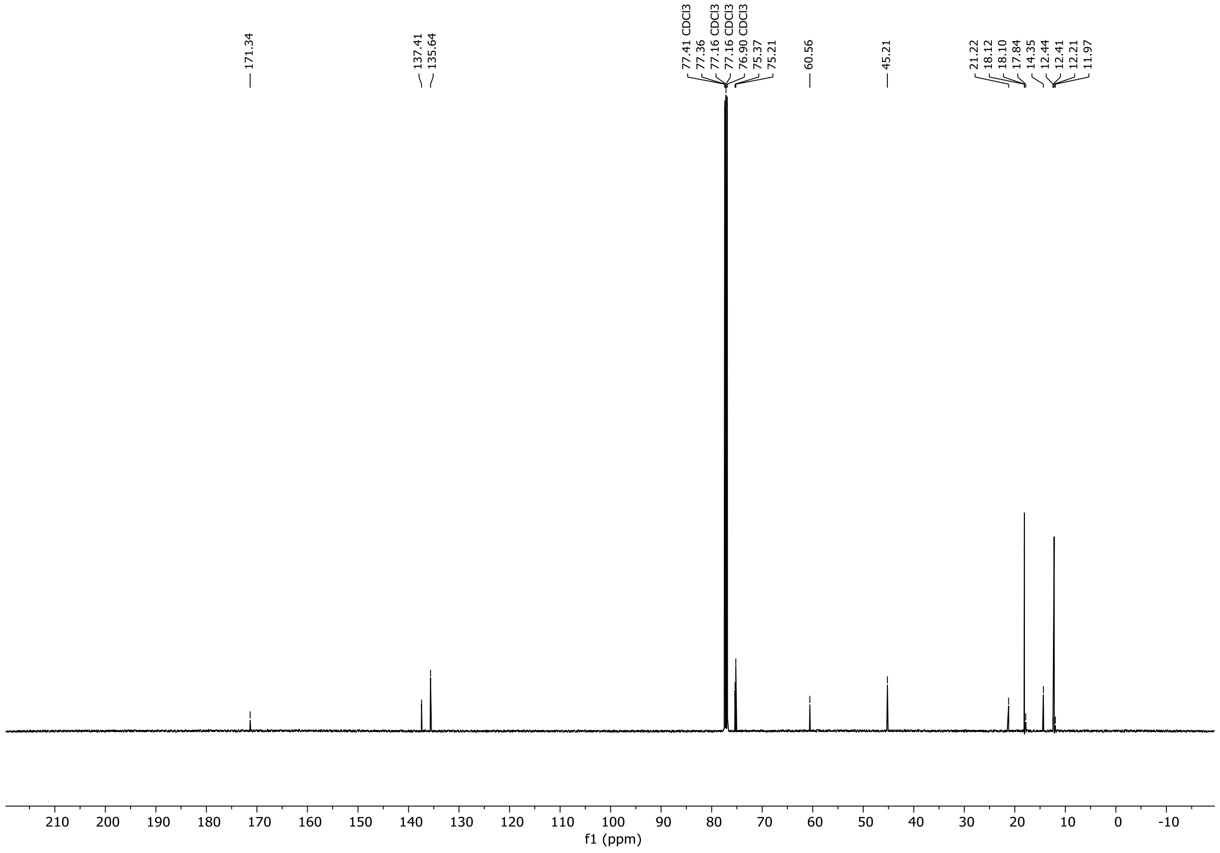

^{1}H-NMR (**20**)

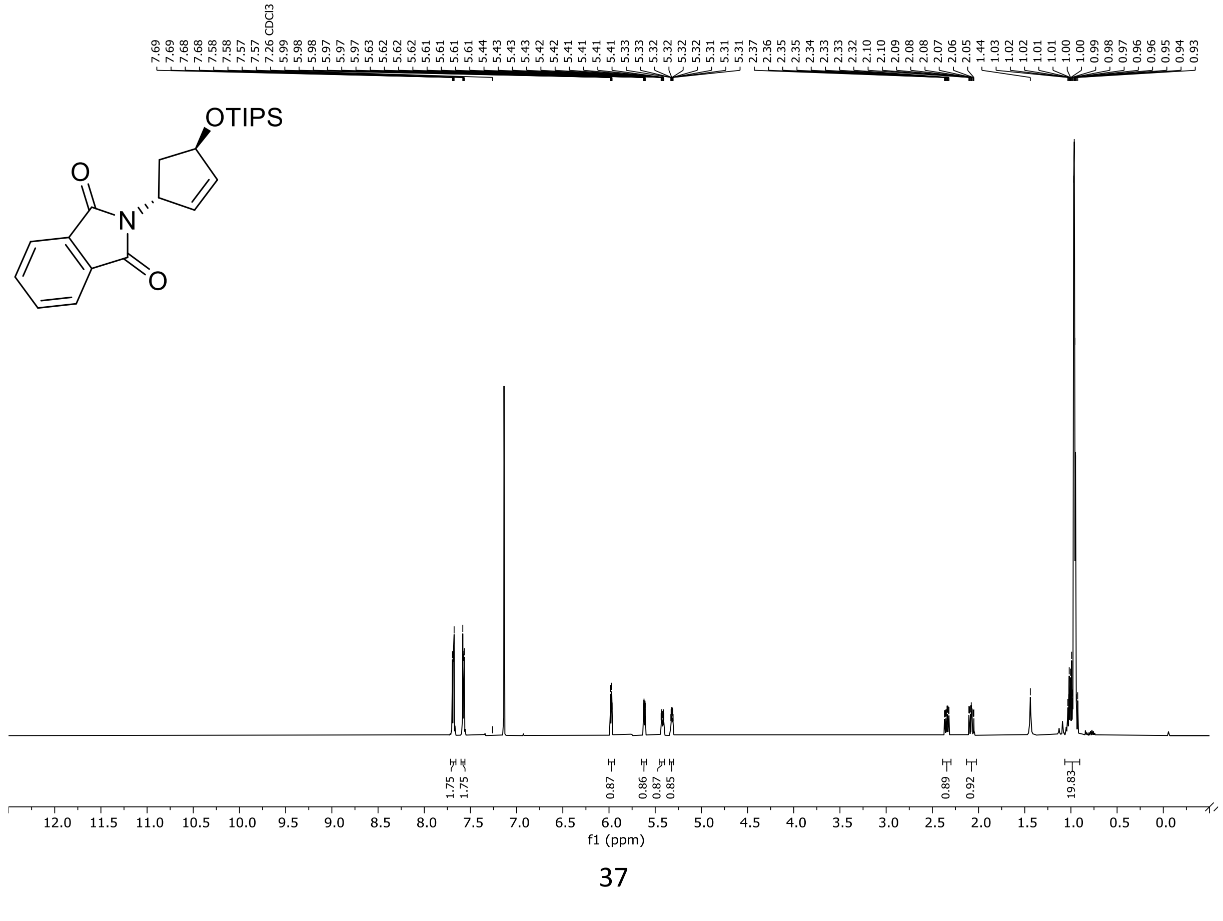

^{13}C-NMR (**20**)

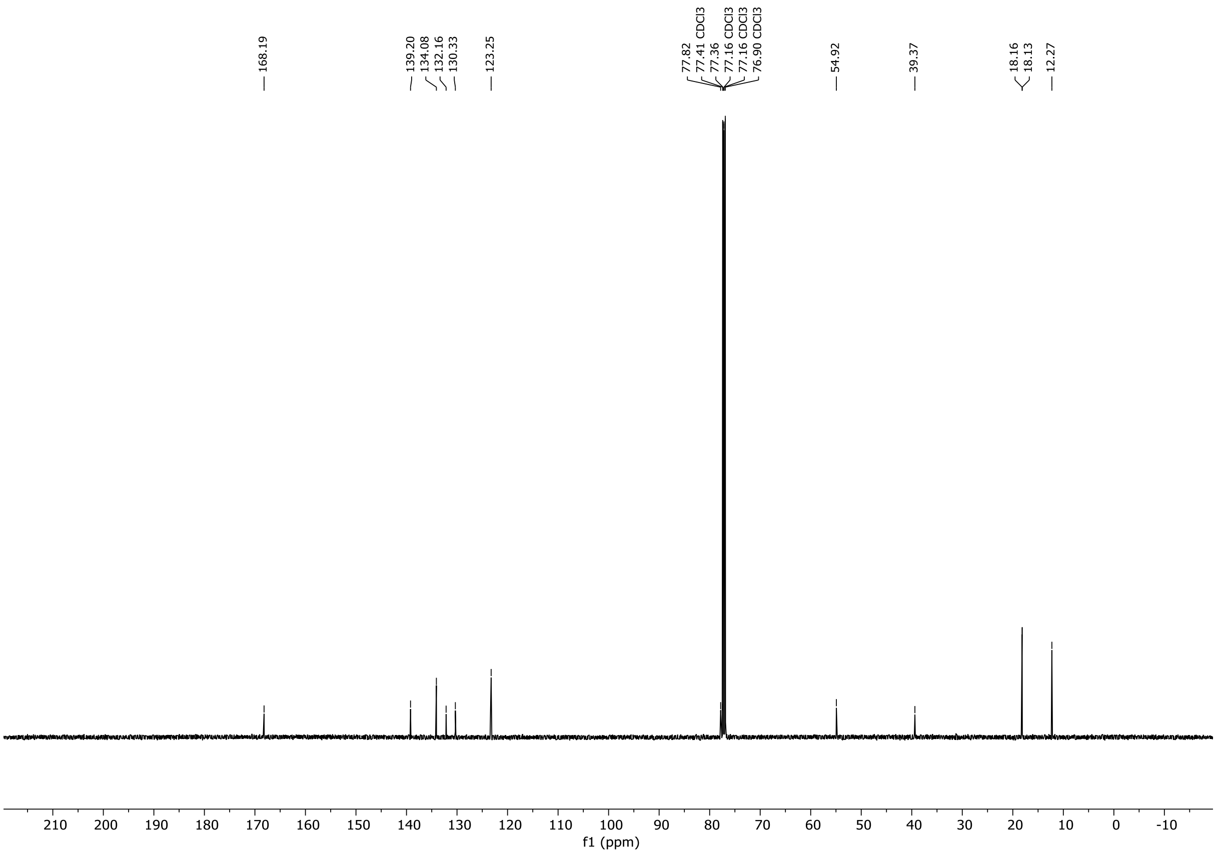

^{1}H-NMR (**13**)

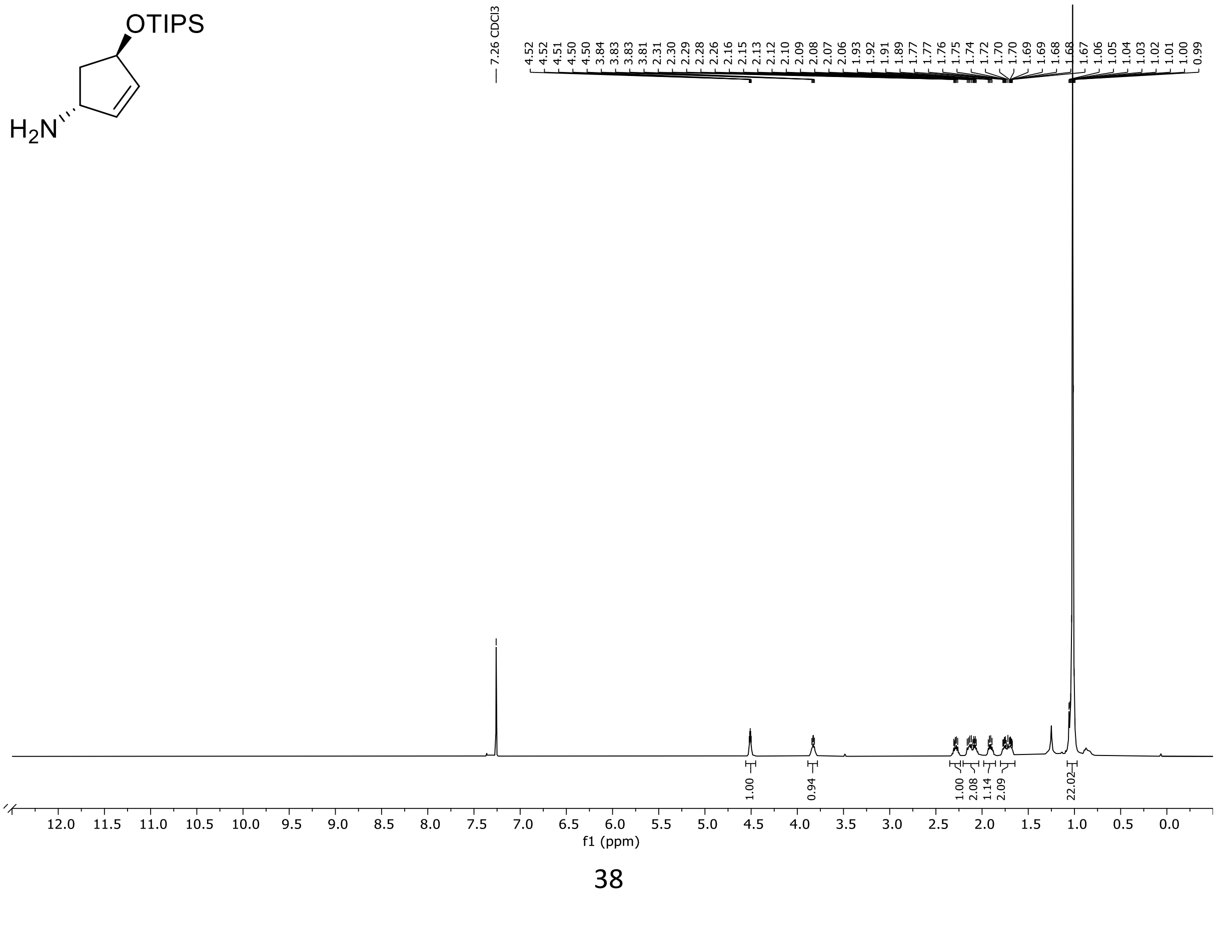

^{13}C-NMR (**13**)

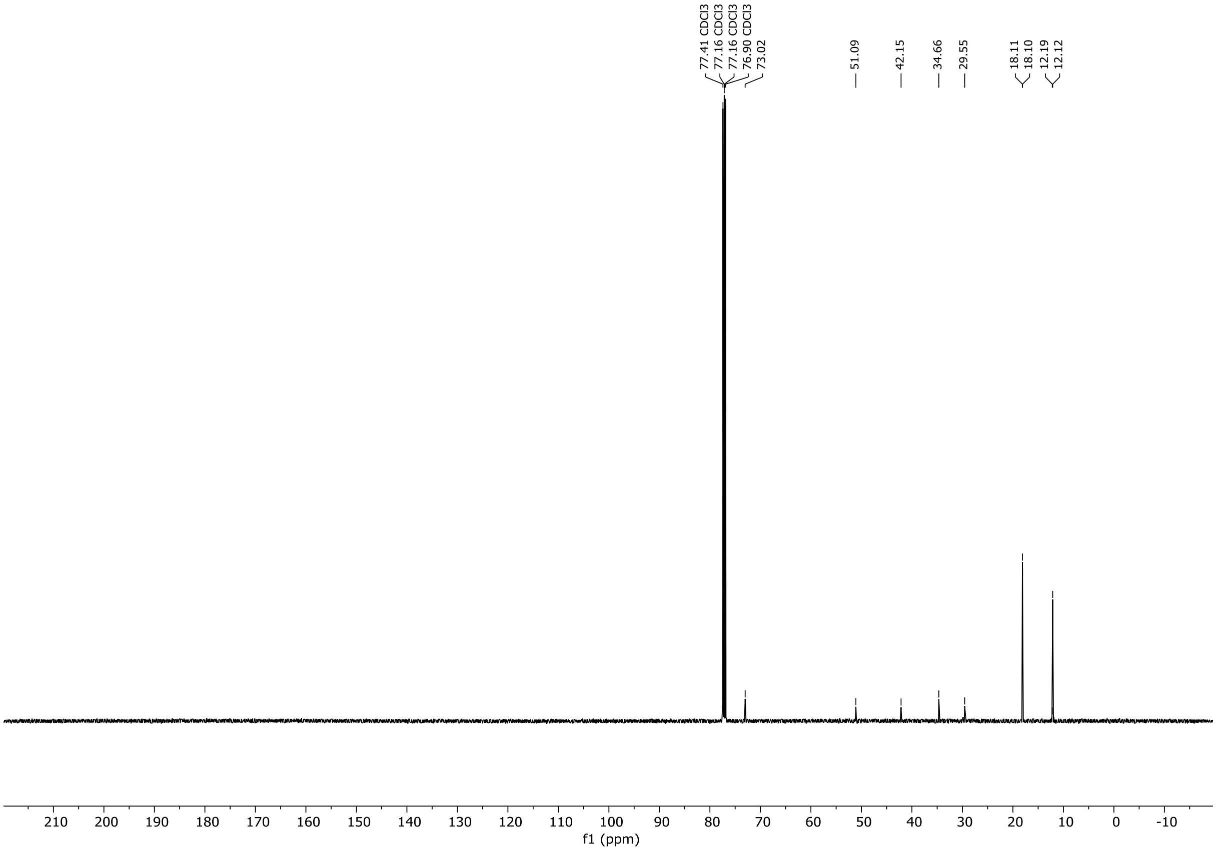

^{1}H-NMR (**21**)

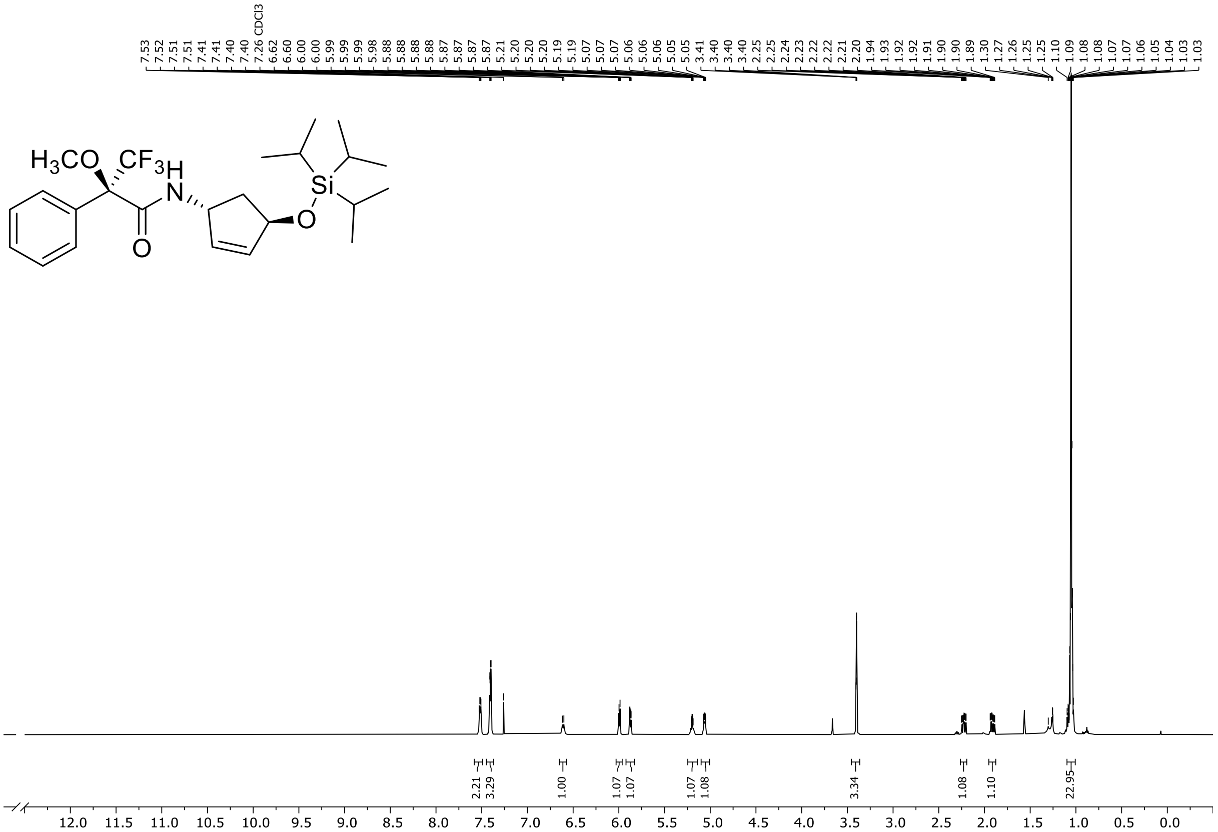

^{13}C-NMR (**21**)

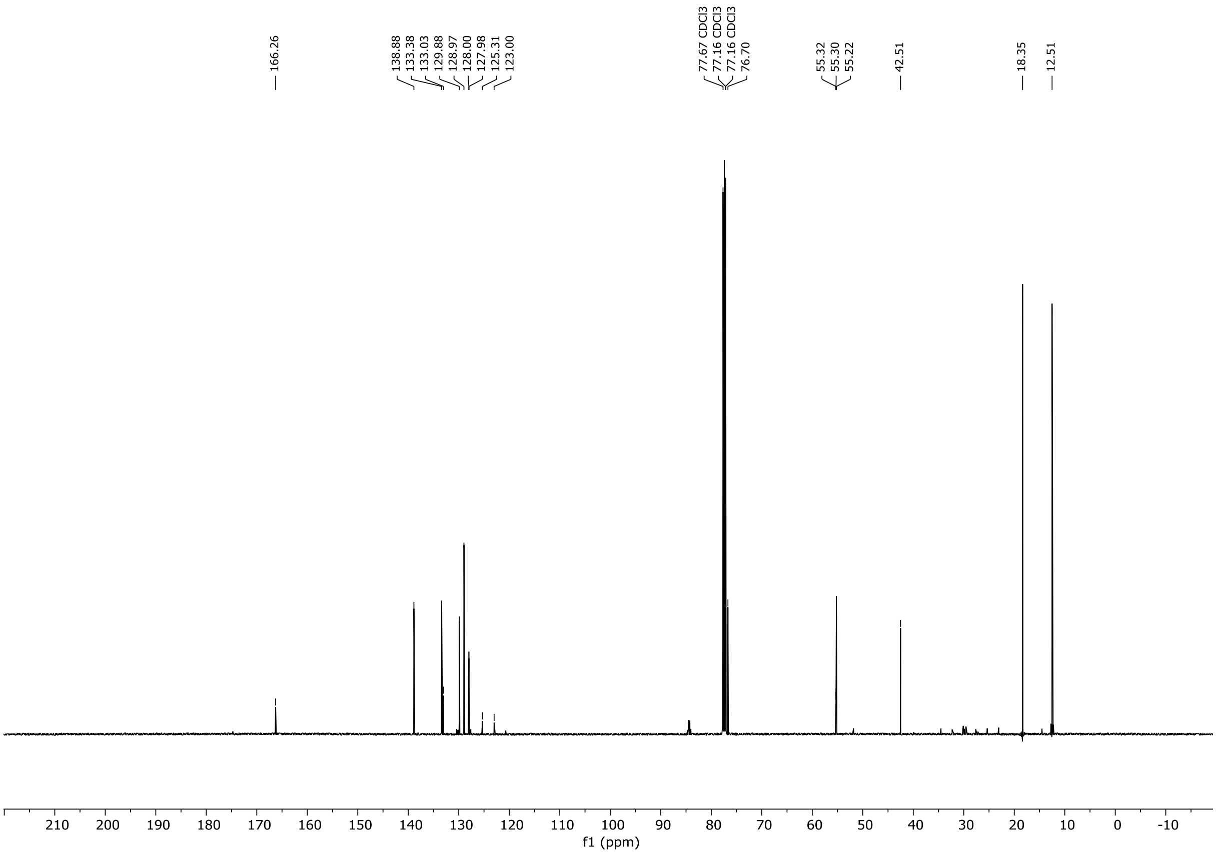

^{19}F-NMR (**21**)

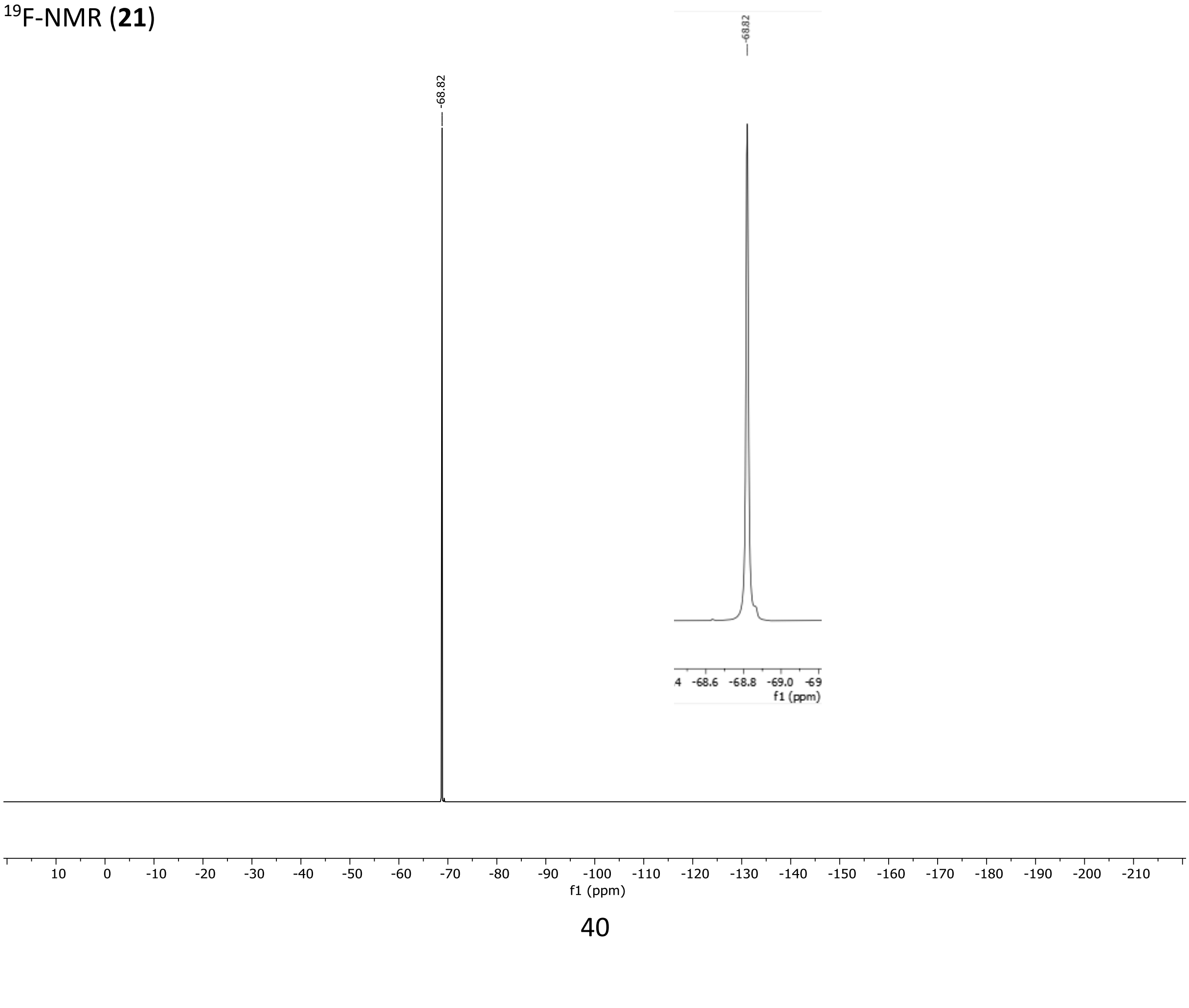

^{1}H-NMR (**11a**)

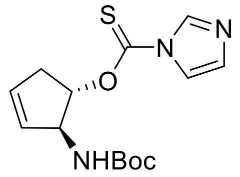

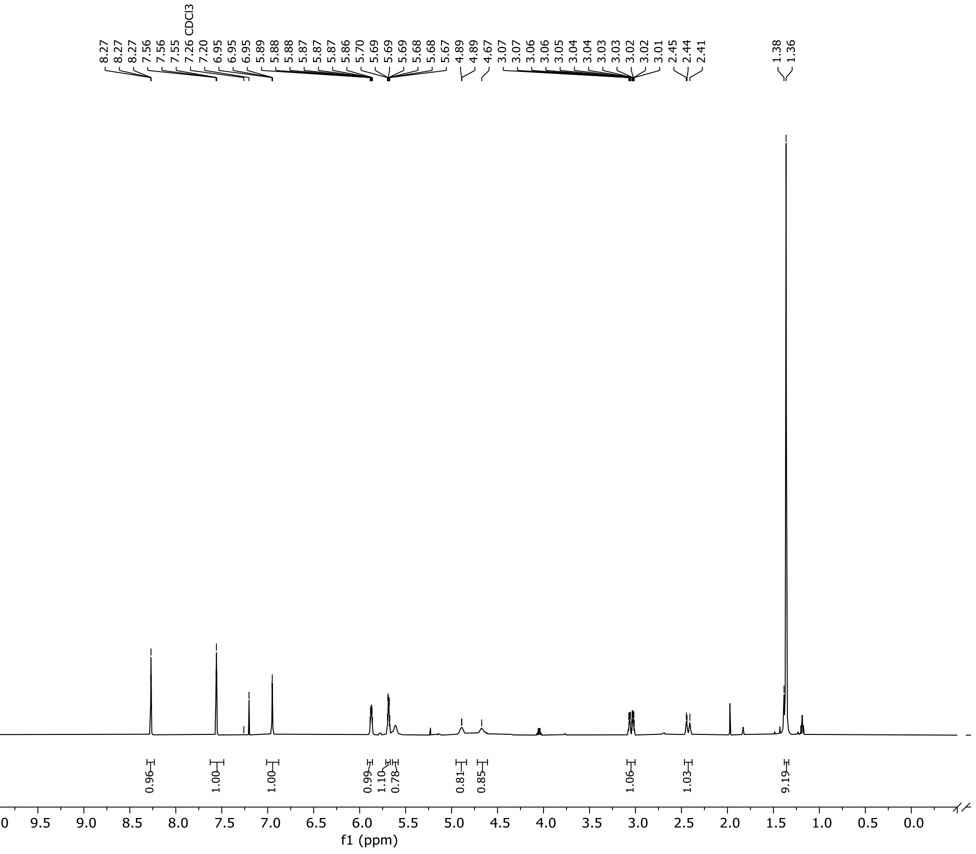

^{13}C-NMR (**11a**)

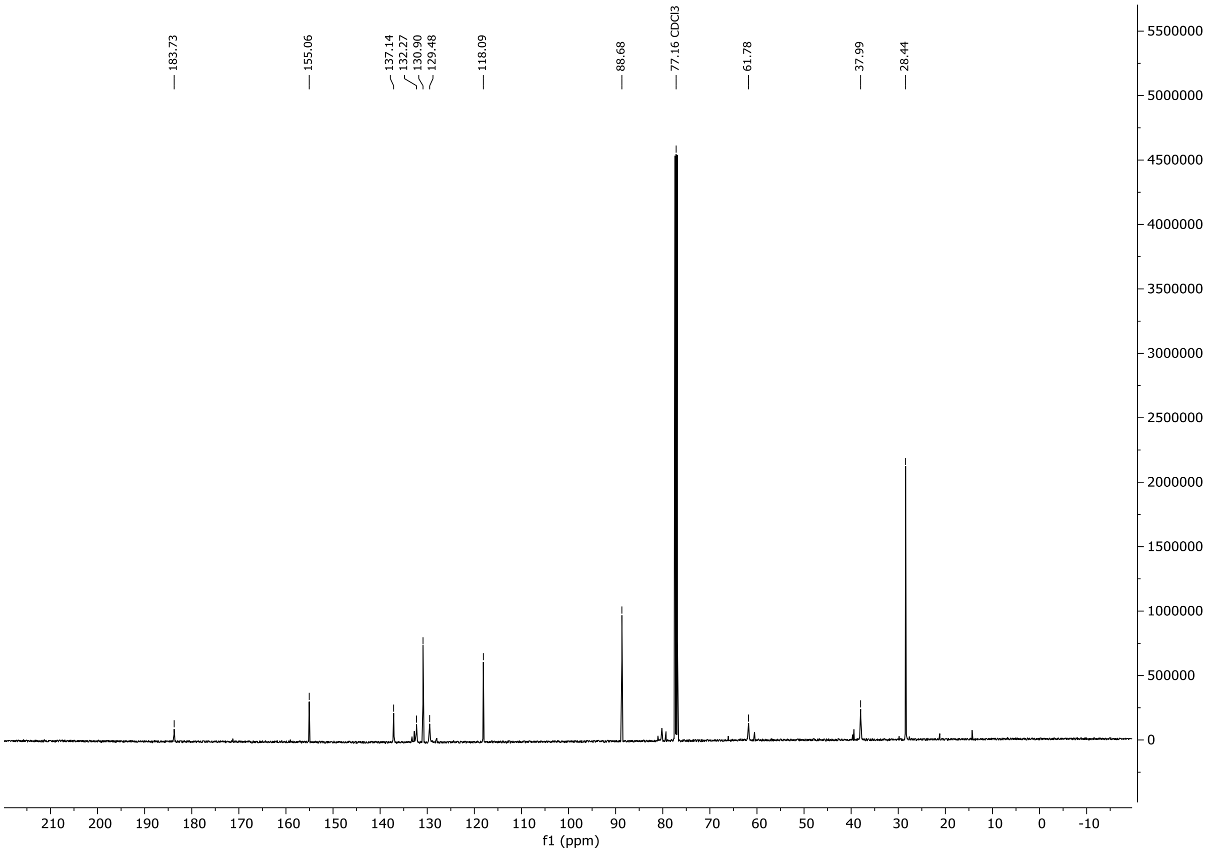

^{1}H-NMR (**11b**)

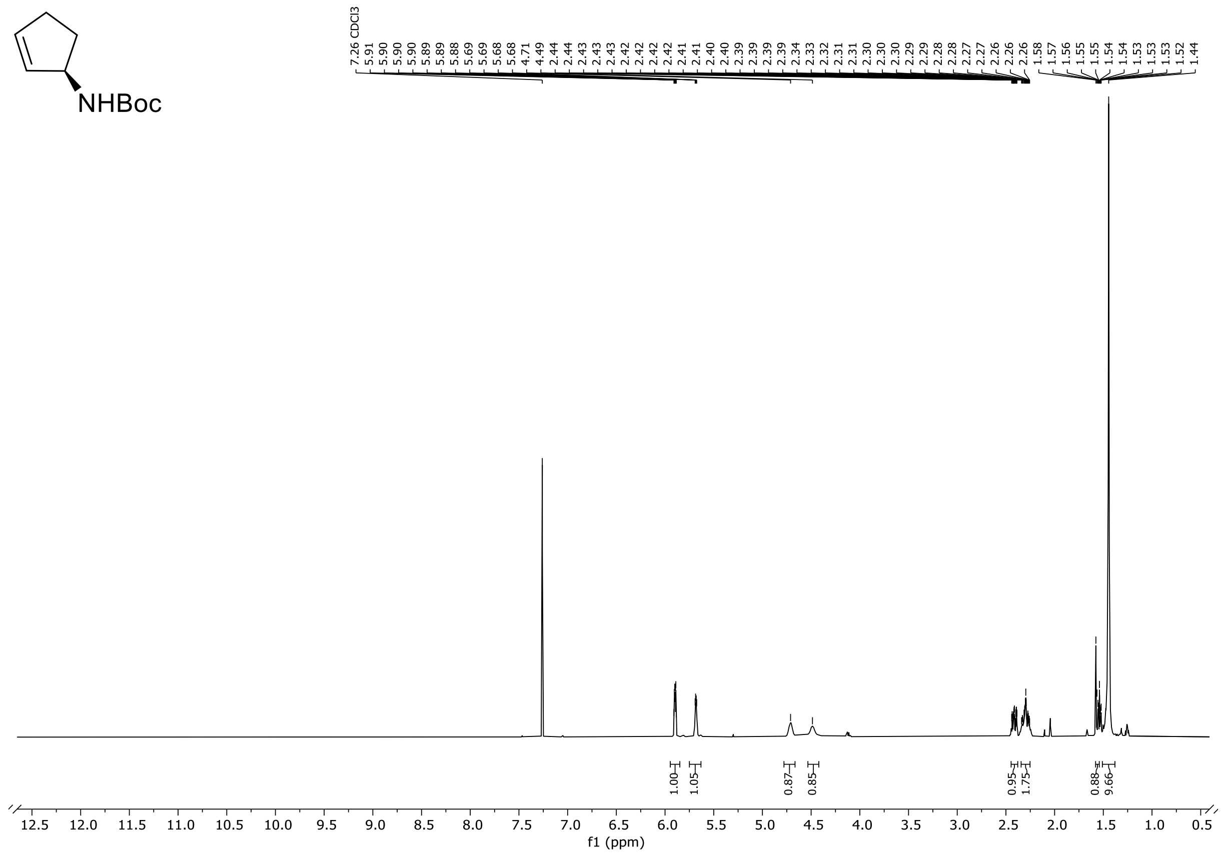

^{13}C-NMR (**11b**)

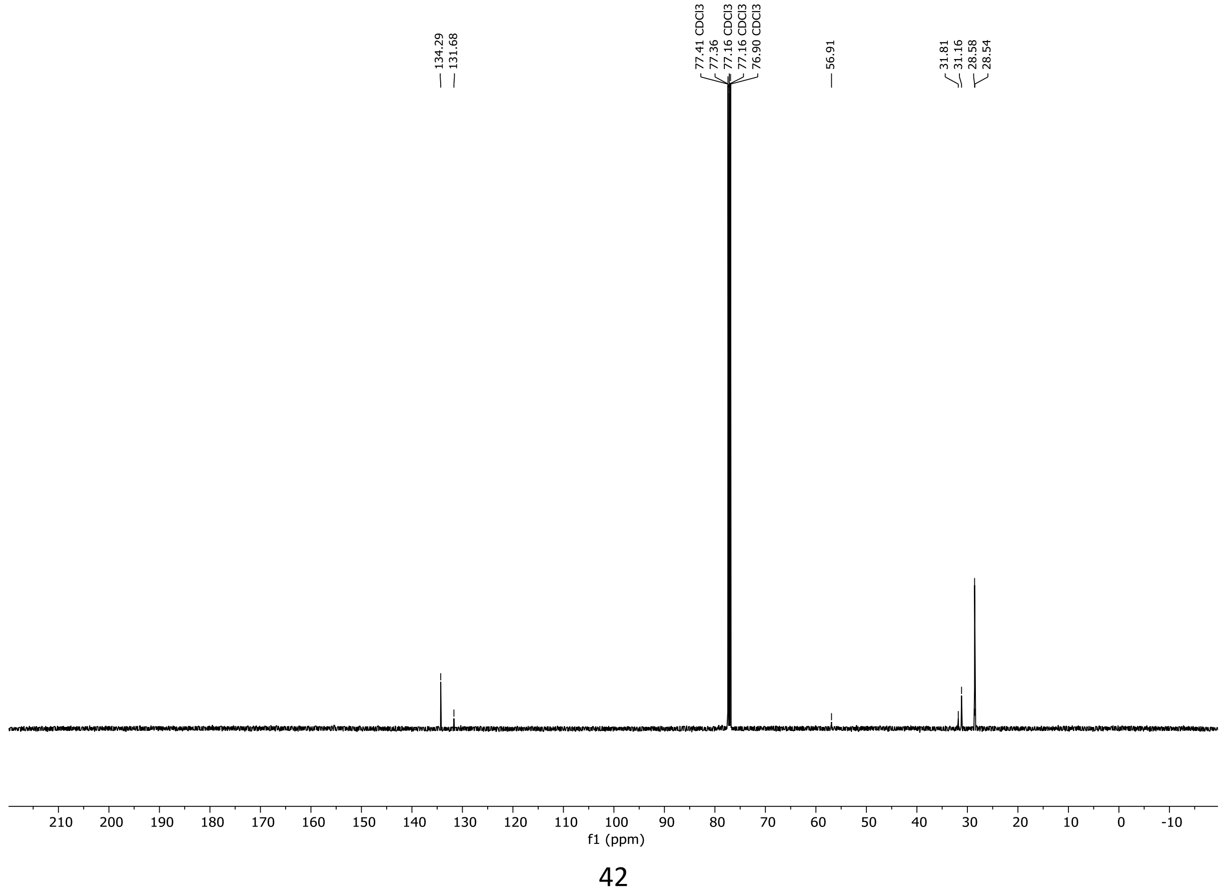

^{1}H-NMR (**22**)

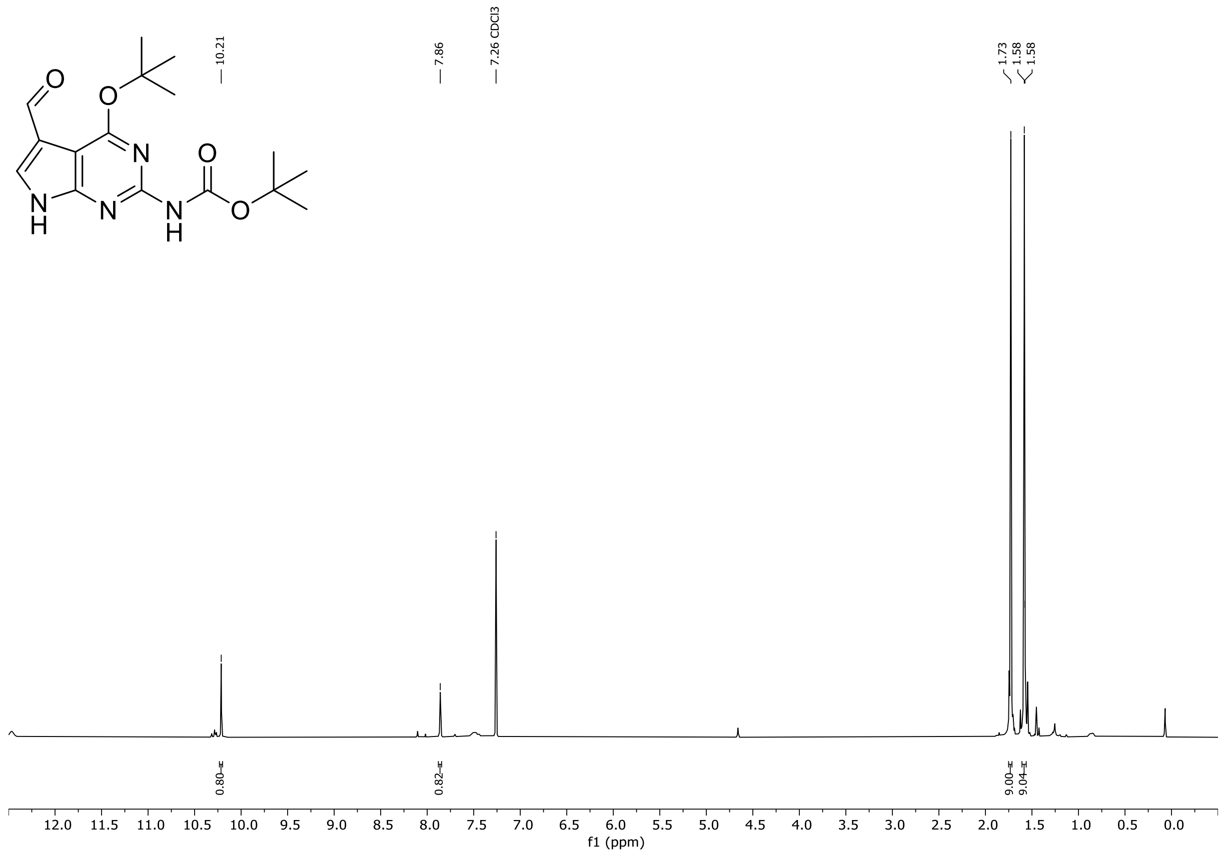

^{13}C-NMR (**22**)

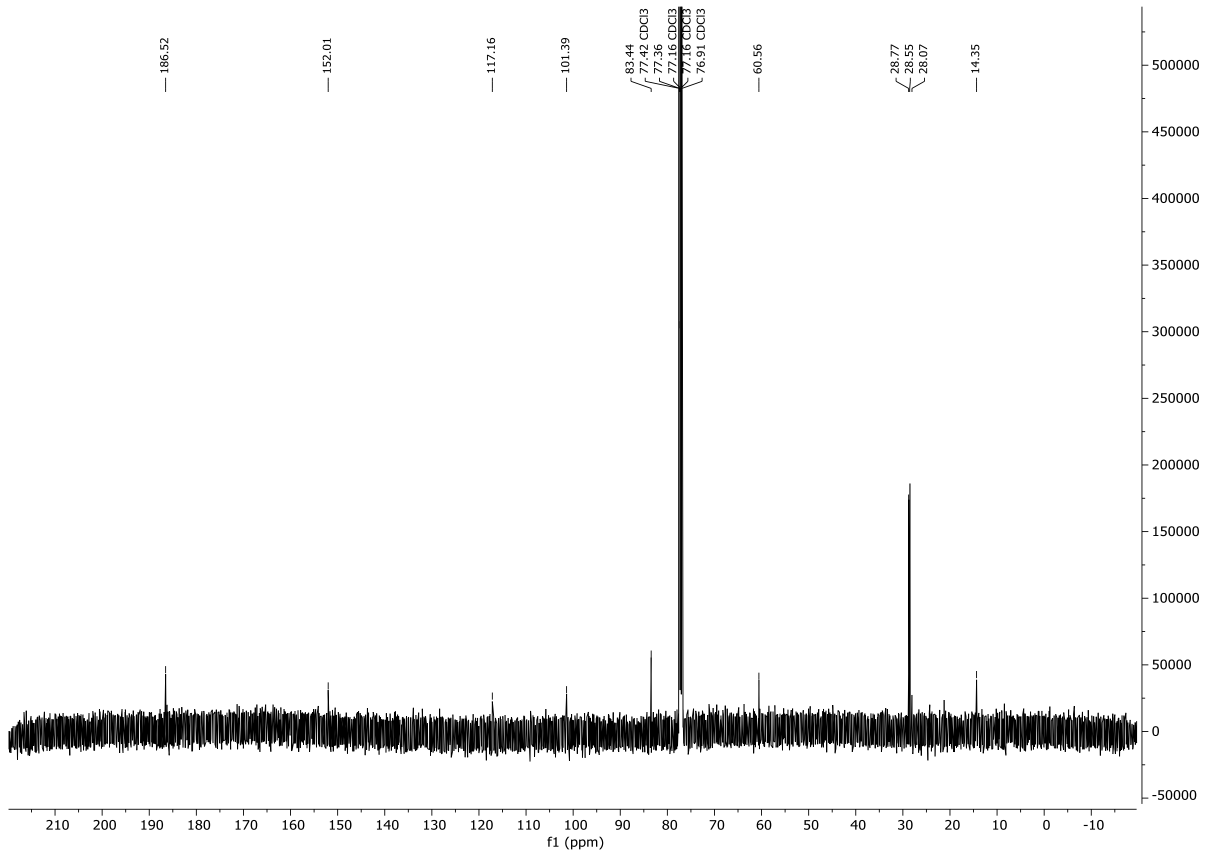

^{1}H-NMR (**23**)

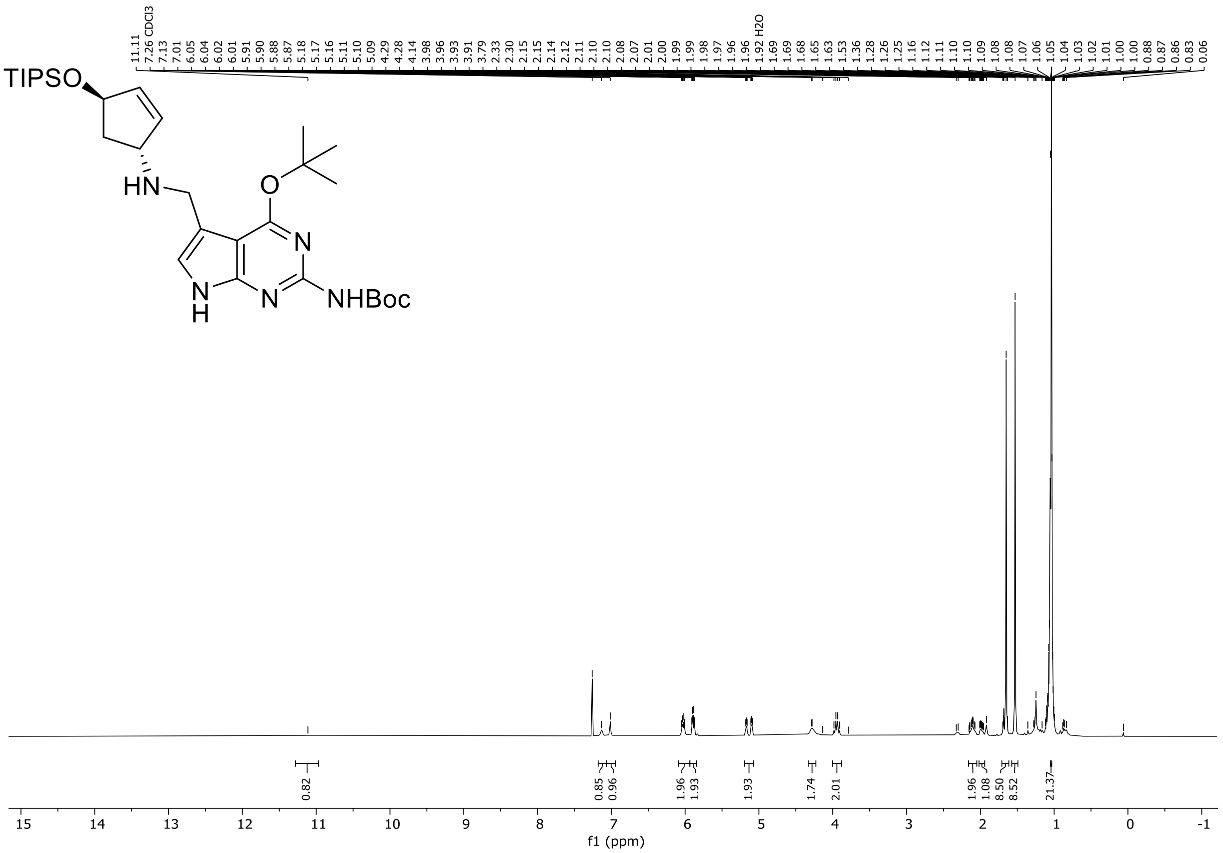

^{13}C-NMR (**23**)

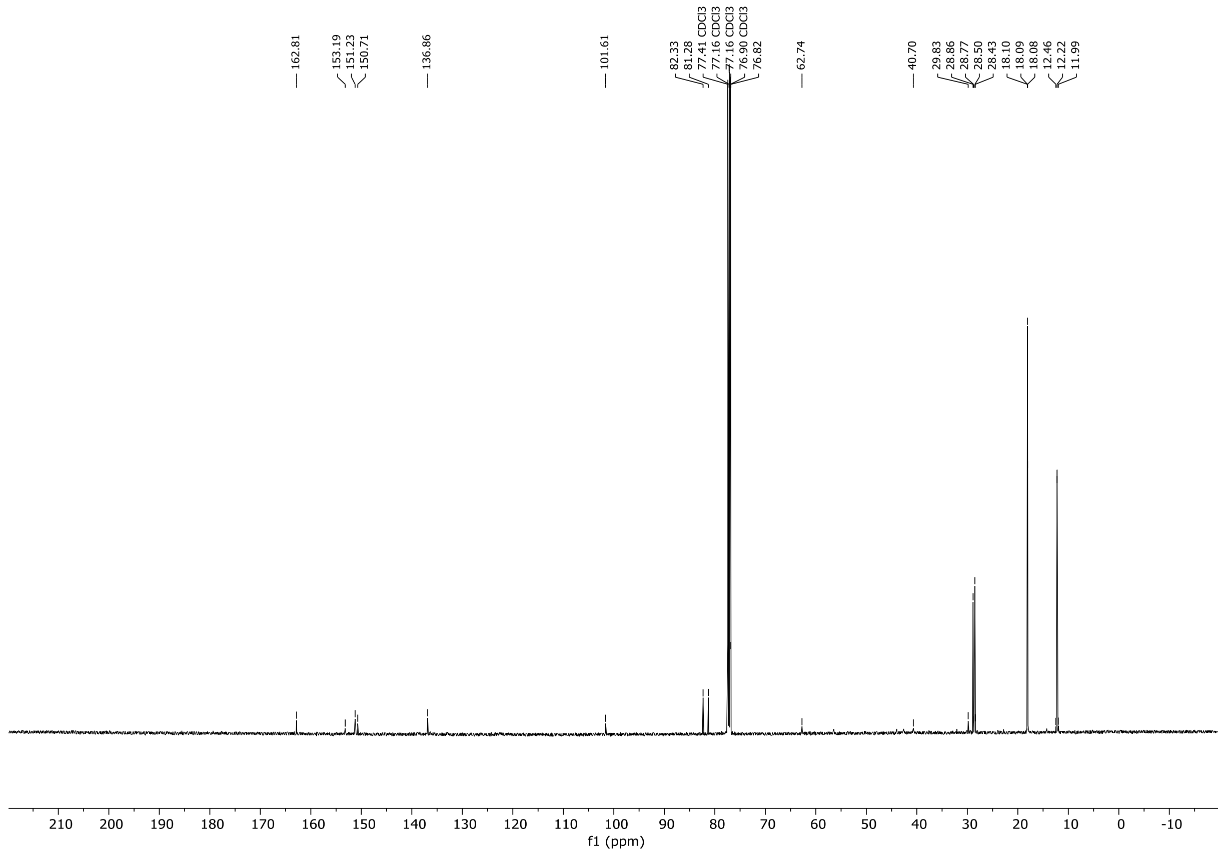

^{1}H-NMR (**24**)

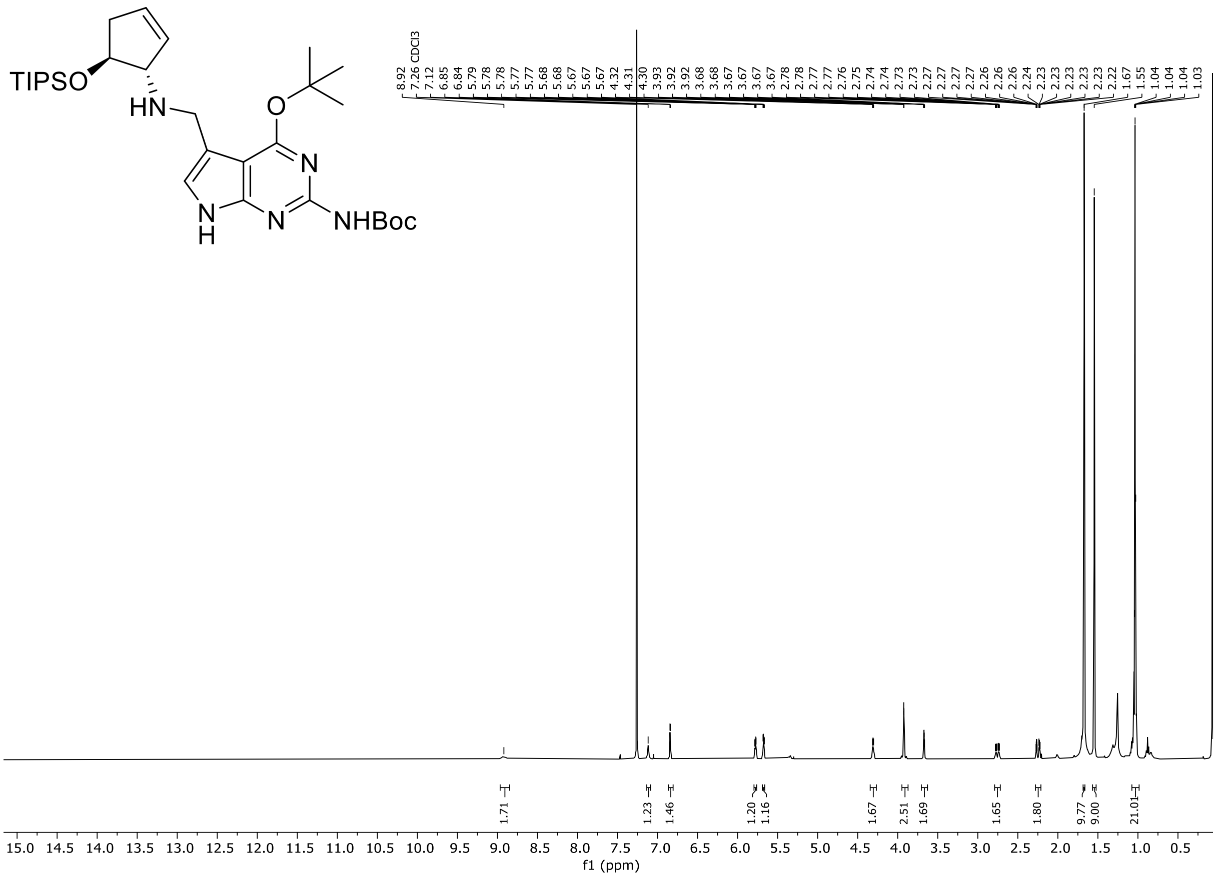

^{13}C-NMR (**24**)

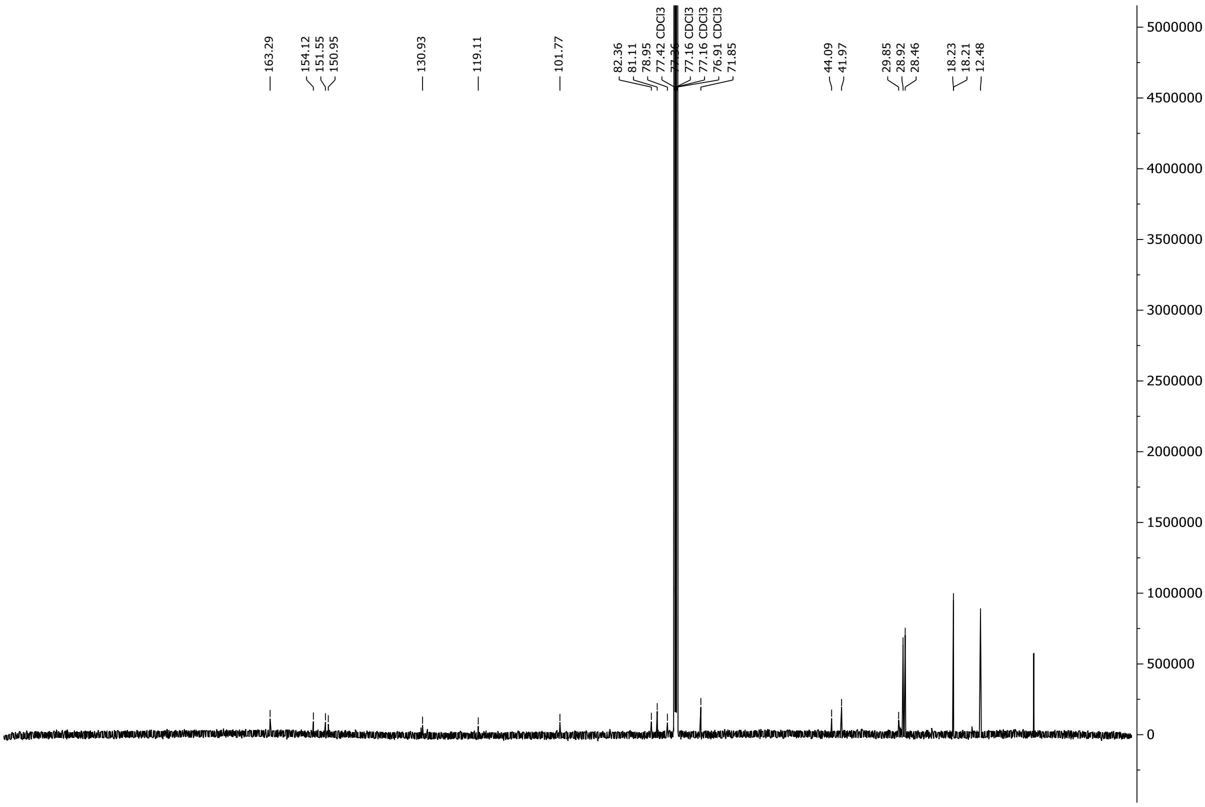

^{1}H-NMR (**1**)

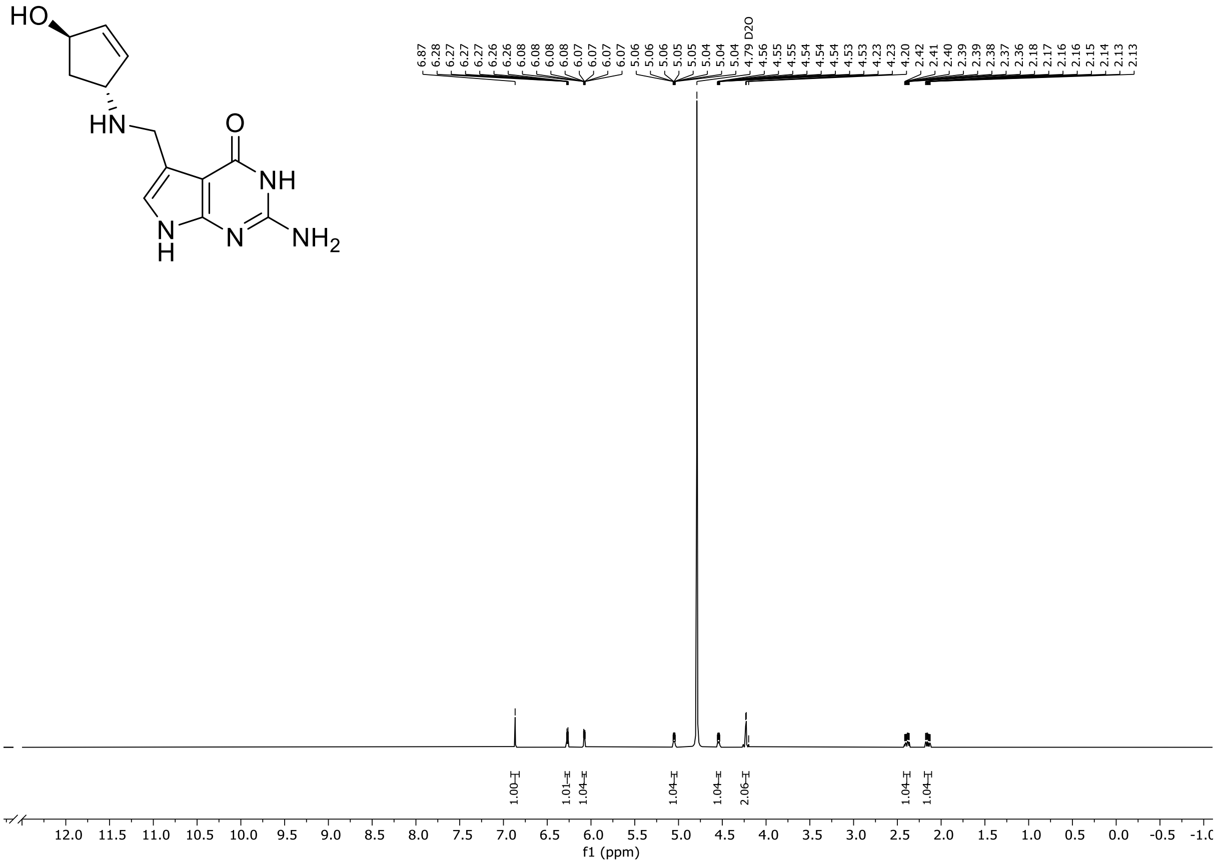

^{13}C-NMR (**1**)

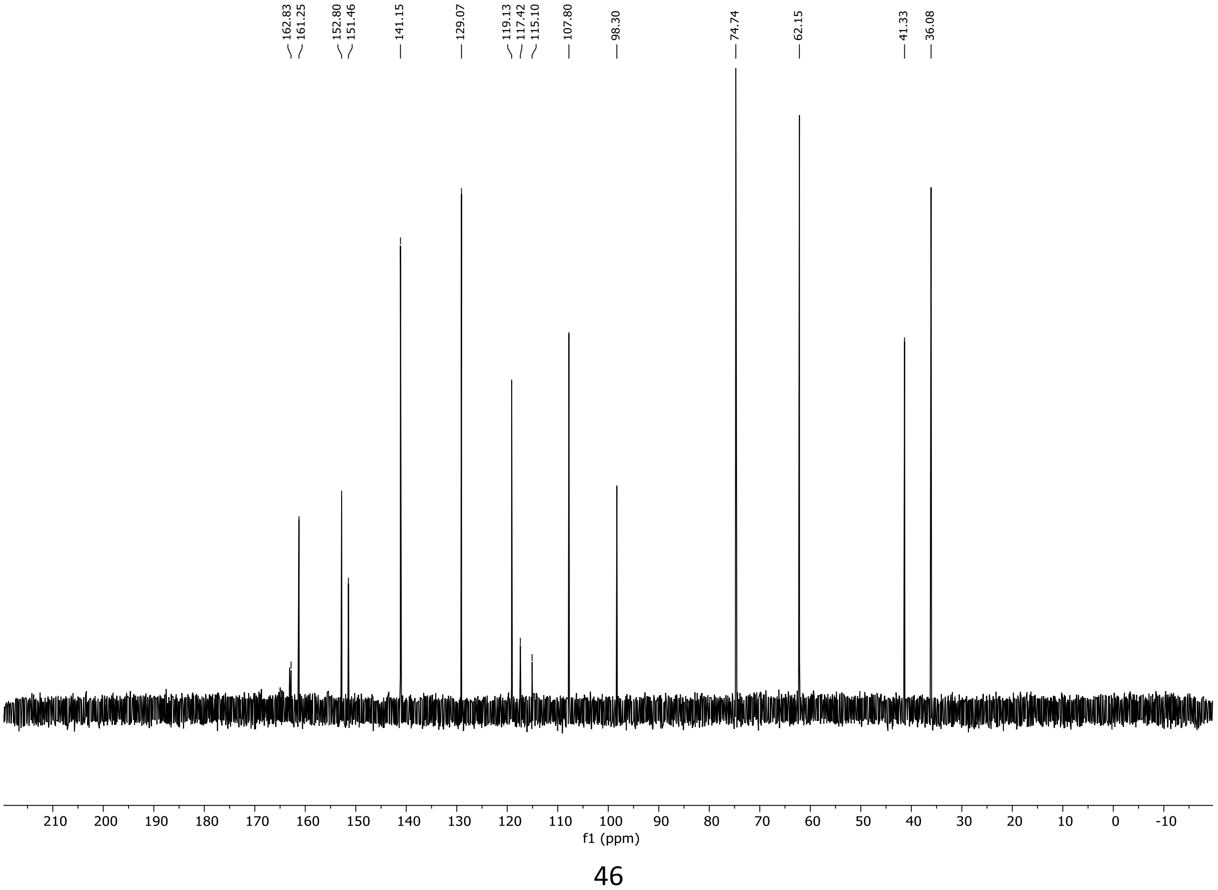

^{1}H-NMR (**2**)

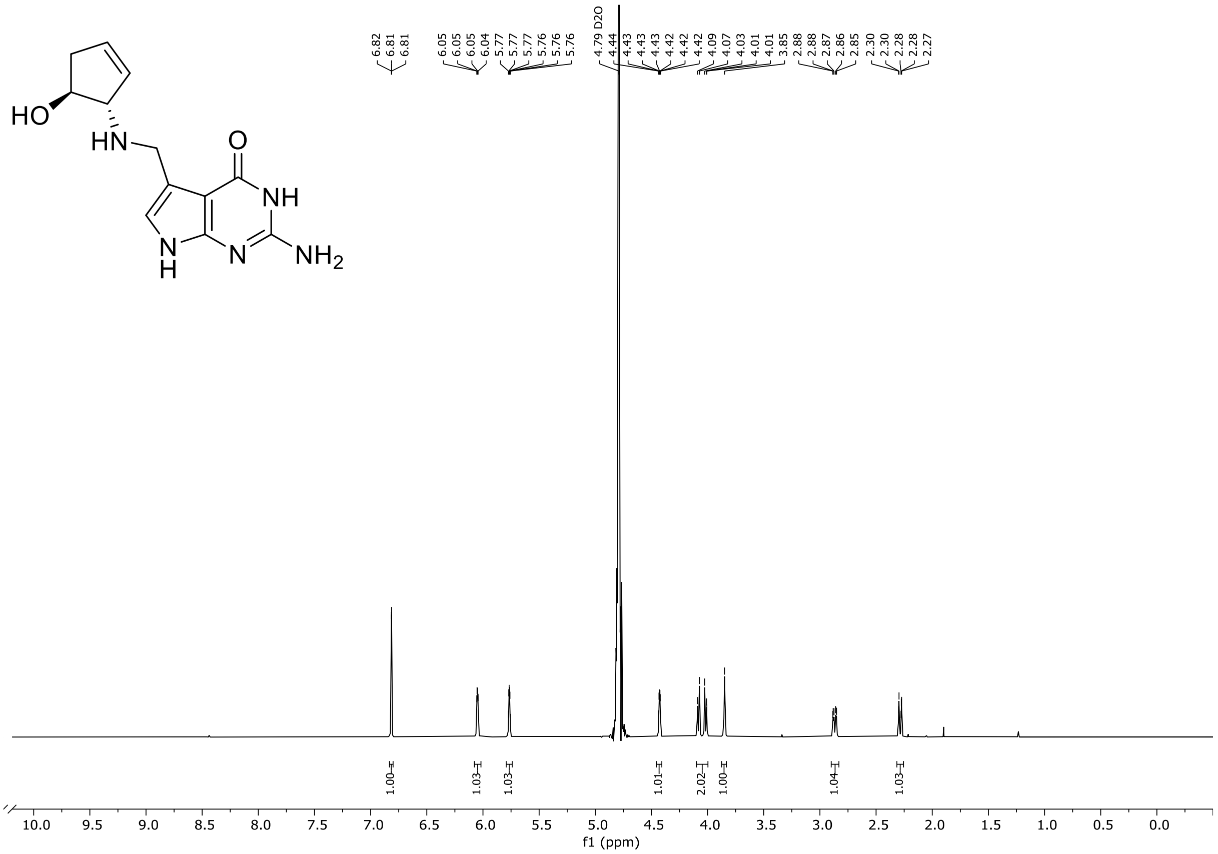

^{13}C-NMR (**2**)

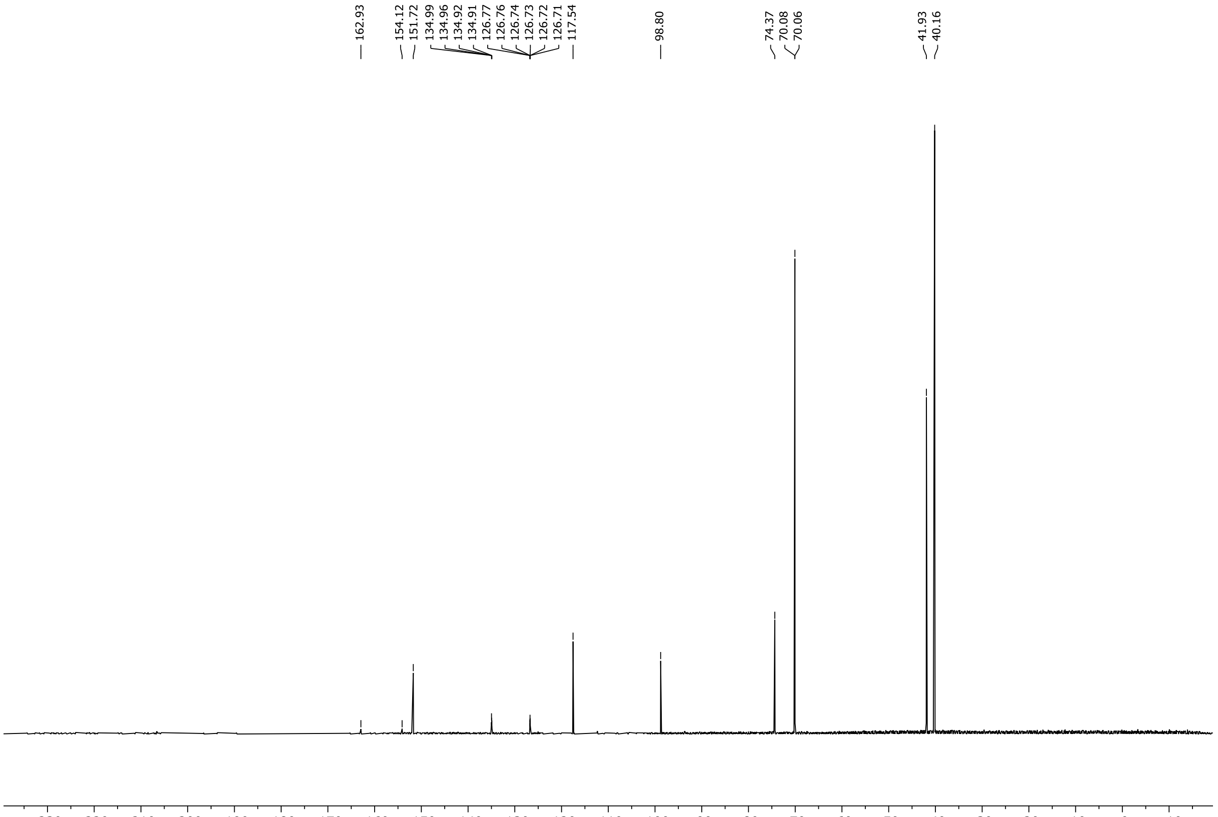

^{1}H-NMR (**3**)

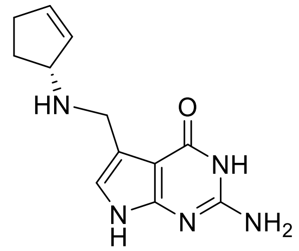

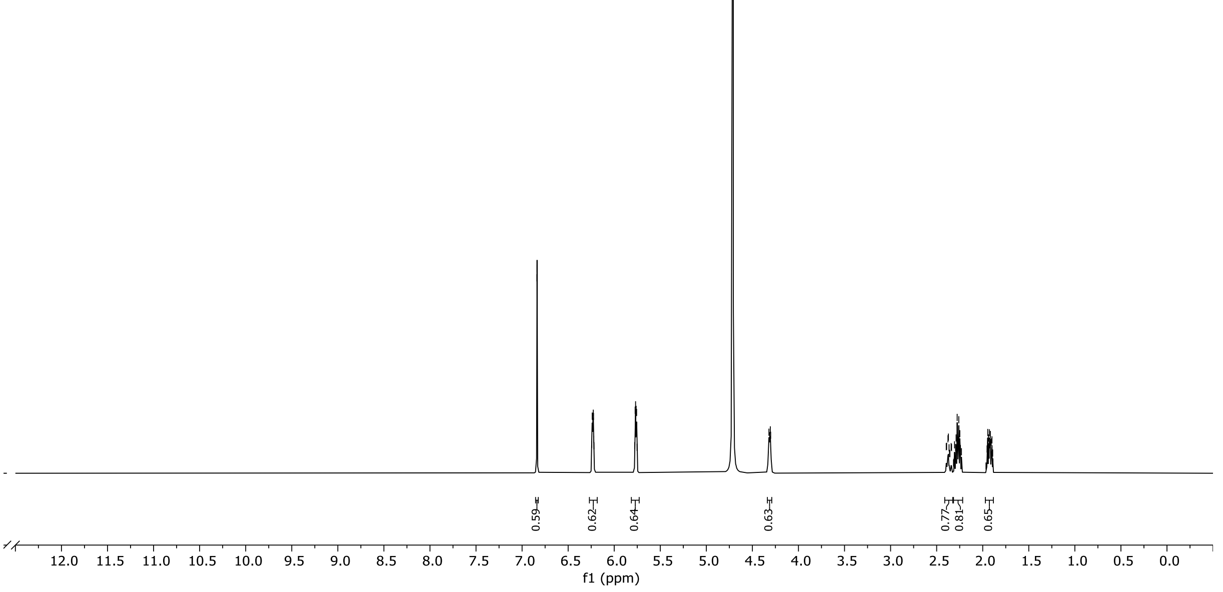

^{13}C-NMR (**3**)

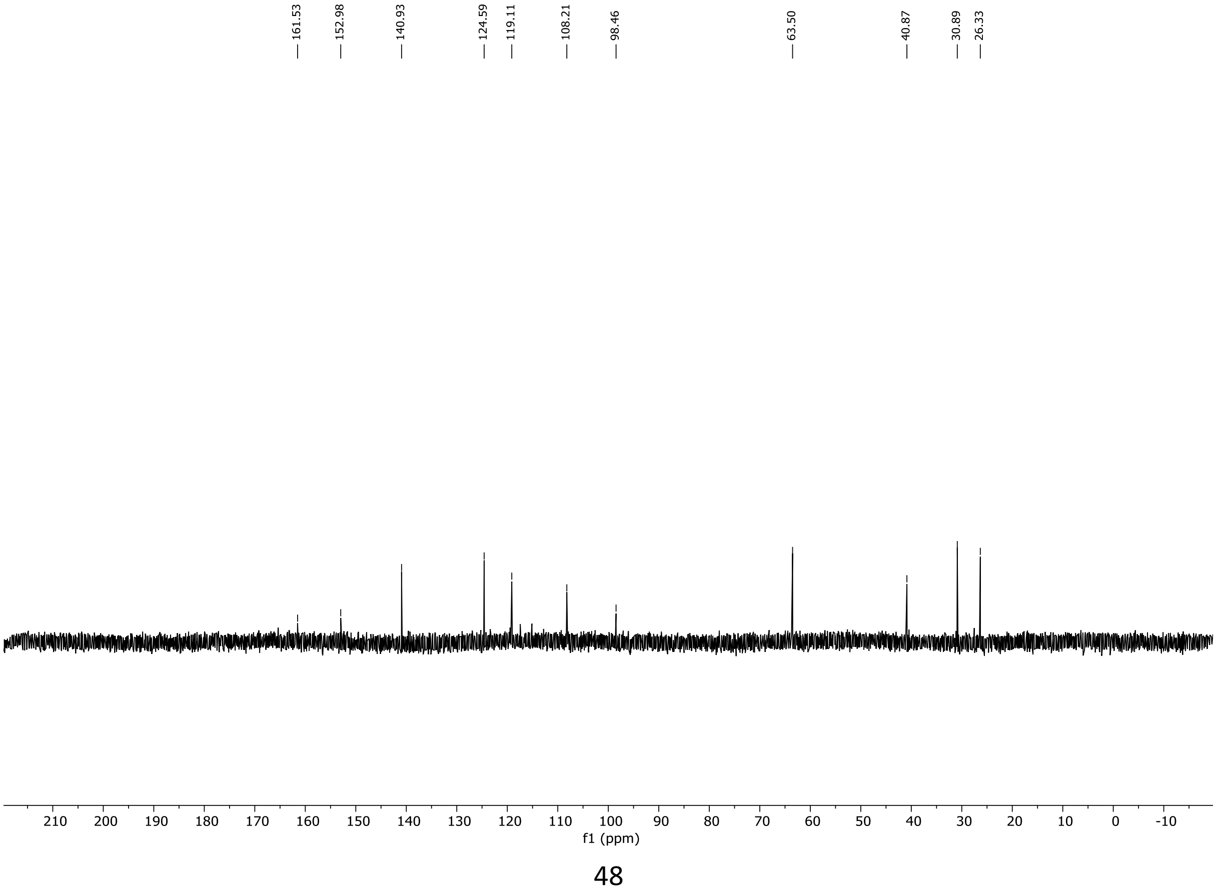

6. References

[1] R. Hauenschild, L. Tserovski, K. Schmid, K. Thuring, M. L. Winz, S. Sharma, K. D. Entian, L. Wacheul, D. L. Lafontaine, J. Anderson, J. Alfonzo, A. Hildebrandt, A. Jaschke, Y. Motorin, M. Helm, *Nucleic Acids Res.* **2015**, *43*, 9950-9964.

[2] M. Heiss, R. V. F., S. and Kellner, *RNA Biol.* **2017**, *14*, 1260-1268.

[3] A. Pichler, M. Hillmeier, M. Heiss, E. Peev, S. Xefteris, B. Steigenberger, I. Thoma, M. Müller, M. Borsò, A. Imhof, T. Carell, *J. Am. Chem. Soc.* **2023**, *145*, 25528-25532.

[4] M. E. Jung, J. A. Berliner, D. Angst, D. Yue, L. Koroniak, A. D. Watson, R. Li, *Org. Lett.* **2005**, *7*, 3933-3935.

[5] R. Kelly, I. Schletter, S. Stein, W. Wierenga, *J. Am. Chem. Soc.* **1979**, *101*, 1054-1056.

[6] S. Hari, G. Adam, R. J. Carl, *Tetrahedron Lett.* **1994**, *35*, 6975-6976.

[7] N. M. Maguire, A. Ford, J. Balzarini, A. R. Maguire, *J. Org. Chem.* **2018**, *83*, 10510-10517.

[8] S. Specklin, A. Dikova, A. Blanc, J.-M. Weibel, P. Pale, *Tetrahedron Lett.* **2014**, *55*, 6987-6991.
